# Long-term efficacy and safety of continued complement C1s inhibition with sutimlimab in cold agglutinin disease: CADENZA study Part B

**DOI:** 10.1016/j.eclinm.2024.102733

**Published:** 2024-07-18

**Authors:** Alexander Röth, Sigbjørn Berentsen, Wilma Barcellini, Shirley D’Sa, Bernd Jilma, Marc Michel, Ilene C. Weitz, Masaki Yamaguchi, Jun-ichi Nishimura, Josephine M.I. Vos, Joan Cid, Michael Storek, Nancy Wong, Ronnie Yoo, Deepthi Jayawardene, Shruti Srivastava, Marek Wardęcki, Frank Shafer, Michelle Lee, Catherine M. Broome

**Affiliations:** a Department of Hematology and Stem Cell Transplantation, West German Cancer Center, University Hospital Essen, University of Duisburg-Essen, Essen, Germany; b Department of Research and Innovation, Haugesund Hospital, Haugesund, Norway; c Fondazione IRCCS Ca’ Granda Ospedale Maggiore Policlinico, Milan, Italy; d UCLH Centre for Waldenström's Macroglobulinemia and Related Conditions, University College London Hospitals NHS Foundation Trust, London, UK; e Department of Clinical Pharmacology, Medical University of Vienna, Vienna, Austria; f Henri-Mondor University Hospital, Assistance Publique-Hôpitaux de Paris, UPEC, Créteil, France; g Keck School of Medicine of USC, Los Angeles, CA, USA; h Ishikawa Prefectural Central Hospital, Japan; i Osaka University Graduate School of Medicine, Japan; j Amsterdam UMC, University of Amsterdam, Amsterdam, Netherlands; k Department of Hemotherapy and Hemostasis, ICMHO, IDIBAPS, Hospital Clinic, Barcelona, Catalonia, Spain; l Sanofi, Cambridge, MA, USA; m Sanofi, Bridgewater, NJ, USA; n Sanofi, Warsaw, Poland; o Division of Hematology, MedStar Georgetown University Hospital, Washington, DC, USA

**Keywords:** Autoimmune haemolytic anaemia, Cold agglutinin disease, Classical complement pathway, Complement C1s, Sutimlimab, Haemolysis

## Abstract

**Background:**

Cold agglutinin disease (CAD) is a rare autoimmune haemolytic anaemia mediated by the classical complement pathway (CP). Sutimlimab selectively targets complement C1s inhibiting classical CP activation. In CADENZA Part A (26-weeks), a placebo-controlled study in patients without recent transfusion history, sutimlimab reduced haemolysis, anaemia, and fatigue, and was generally well tolerated.

**Methods:**

The CADENZA study (NCT03347422) started in March 2018 (Part A) and completed in December 2021 (Part B). All patients in Part B were eligible to receive sutimlimab for up to 1 year after the last patient completed Part A. Efficacy and safety was assessed throughout Part B, until the last on-treatment visit with available assessment (LV), and after a 9-week washout.

**Findings:**

In total, 32/39 patients completed Part B; median treatment duration: 99 weeks. Similar sustained improvements in haemolysis, anaemia, and quality of life were observed in patients switching to sutimlimab and those continuing sutimlimab. Mean LV values for the combined group (ie, placebo-to-sutimlimab group and sutimlimab-to-sutimlimab group) improved from baseline for haemoglobin (≥11.0 g/dL on-treatment *vs* 9.3 g/dL at baseline), bilirubin (≤20.0 μmol/L on-treatment *vs* 35.0 μmol/L at baseline), and FACIT-Fatigue scores. Following a 9-week washout, inhibition of CP activity was reversed, and haemolytic markers approached baseline levels. Overall, sutimlimab was generally well tolerated throughout the study. No patients developed systemic lupus erythematosus or meningococcal infections. During the 9-week washout, most adverse events could be attributed to recurrence of underlying CAD.

**Interpretation:**

The CADENZA Part B results support the sustained efficacy and safety of sutimlimab for treatment of CAD; however, upon discontinuation disease activity reoccurs.

**Funding:**

Sanofi.

**Research in context**

**Evidence before this study**

The literature suggests that treatment approaches and responses to treatment are often inconsistent using the traditional unapproved therapies for cold agglutinin disease (CAD). A PubMed title/abstract search for the terms “cold agglutinin disease” and “sutimlimab” excluding “warm autoimmune haemolytic anaemia” in the last 10 years provides a total of 27 articles. Sutimlimab was first approved in 2022 for the treatment of CAD in the US, EU and Japan and is, as of now, the only approved therapy for the treatment of CAD. The approval was based on the results from Part A of two, 26-week, phase 3 trials, CARDINAL and CADENZA. In both trials it was found that treatment with sutimlimab results in rapid and sustained efficacy in patients with CAD (with and without a history of transfusion), halting haemolysis, markedly increasing haemoglobin levels, and improving quality of life.

**Added value of this study**

CADENZA Part B is the first trial to demonstrate the long-term efficacy and safety of sutimlimab in patients with CAD and no history of transfusion. Patients received sutimlimab for a median duration of 99 weeks. This included patients who received placebo in Part A and switched to sutimlimab during the Part B open-label extension. Part B also included a 9-week washout period, which allowed assessment of disease activity in CAD following cessation of sutimlimab.

**Implications of all the available evidence**

Sutimlimab has been found to be generally well tolerated, across trials, with adverse events consistent with an older, medically complex patient population. This study supports the durability of response to treatment with sutimlimab for a median duration of treatment of 99 weeks and indicates a need in many patients for continuous treatment to maintain the favourable treatment effects. The safety and effectiveness of sutimlimab in patients with CAD in the real world will be further assessed in the CADENCE registry.

## Introduction

Cold agglutinin disease (CAD) is a rare subtype of autoimmune haemolytic anaemia (AIHA) mediated by classical complement pathway (CP) activation.^1^^,^^2^ CAD accounts for 15–30% of all AIHAs.^3^^,^^4^ In CAD, a low-grade lymphoproliferative disorder, cold agglutinin autoantibodies, usually of the immunoglobulin M (IgM)-κ class, are produced by clonal B cells.^1^^,^^2^ In the majority of cases these IgM-κ autoantibodies recognise the “I” antigen, which is present on the surface of red blood cells (RBC), and the recognition of RBC antigens can cause RBC agglutination at or below 37°C.^1^^,^^2^ The cold agglutinin–antigen complexes on RBCs trigger the classical CP, which leads to RBC destruction predominantly by extravascular haemolysis and phagocytosis, occurring mainly in the liver, and to a lesser extent, intravascular haemolysis via the terminal complement cascade.^1^^,^^2^

Clinical manifestations of CAD include complement-mediated chronic haemolysis, anaemia, and profound fatigue, as well as agglutination-mediated circulatory symptoms such as acrocyanosis and Raynaud's phenomenon.^3^^,^^5^^,^^6^ Symptoms of CAD are diverse, often non-specific, and negatively impact quality of life (QoL).^7^^,^^8^ Patients with CAD are also at increased risk of thromboembolism and early death.^4^^,^^9^^,^^10^

In CAD, binding of C1 complex (C1q, C1r, and C1s) to a cold–agglutinin–antigen complex on the surface of RBCs activates the classical complement cascade. Sutimlimab is a humanised monoclonal IgG4 antibody which selectively inhibits the activation of the classical CP at the initial stage by binding to and inhibiting C1s, leaving the lectin and alternative pathways intact.^11^, ^12^, ^13^

Sutimlimab is the first approved therapy for CAD, with the first approvals taking place in the US, EU, and Japan in 2022.^14^, ^15^, ^16^ The phase 3, CARDINAL study (Part A, 26 weeks) showed sutimlimab rapidly halted haemolysis, improved haemoglobin (Hb) levels, and reduced fatigue in patients with CAD with a recent history of transfusion^13^^,^^17^; the 2-year extension (Part B) demonstrated that the effects of sutimlimab were sustained but that disease activity reoccurs following treatment cessation.^18^ The phase 3, CADENZA study (Part A, 26 weeks) demonstrated sutimlimab, but not placebo, led to improvements in mean Hb levels and markers of haemolysis, and clinically meaningful improvements in fatigue in patients with CAD and no recent history of transfusion.^19^ This manuscript reports the results of Part B of the open-label extension of the CADENZA study, including the 9-week “washout” period. CADENZA Part B aims to assess the long-term safety and tolerability of sutimlimab treatment in patients with CAD, as well as durability of response to treatment.

## Methods

### Study design and patient population

CADENZA (NCT03347422) was a phase 3, randomised, double-blind, placebo-controlled trial to assess the efficacy and safety of sutimlimab in patients with CAD. Data were collected from 53 study locations, including sites in the United States, Australia, Austria, Belgium, Canada, France, Germany, Israel, Italy, Japan, Netherlands, Norway, Spain, and the United Kingdom. The results of Part A of the study, which consisted of the 26-week pivotal phase, was completed in September 2020.^20^ In brief, adult patients with a confirmed diagnosis of CAD, Hb ≤10.0 g/dL, bilirubin level above the normal reference range, ferritin level above the lower limit of normal, and no recent history of blood transfusion were randomised 1:1 to receive an intravenous infusion of sutimlimab (6.5 g for body weight <75 kg; 7.5 g for ≥75 kg) or placebo (500 mL) on day 0, 7, and every 14 days thereafter through week 25. Full details of the inclusion and exclusion criteria are provided in the Supplemental appendix.

Here we report the results of Part B of the study, which was completed in December 2021. Part B included an open-label extension for up to 1 year after the last patient completed Part A and a 9-week washout period following the last dose of sutimlimab in Part B (Supplementary Figure S1). For patients who completed Part A and qualified to continue in Part B, a crossover loading dose was administered in a blinded manner, at week 26, allowing patients who had received placebo in Part A to receive the sutimlimab loading dose at the start of the Part B dosing schedule, while patients who had received sutimlimab in Part A were given a placebo dose to maintain blinding. Thereafter, all patients continued to receive sutimlimab every 2 weeks from week 27 for up to 1 year after the last patient completed Part A. While enrolled in this study, patients were not supposed to receive protocol-prohibited CAD medications which included rituximab monotherapy or rituximab combination therapies (eg, with bendamustine, fludarabine, ibrutinib, or cytotoxic drugs). Patients who completed Part B were assessed again at an end-of-study safety follow-up (SFU) visit 9 weeks after the last dose. In addition to serving as an SFU, this visit provided the opportunity to assess the effect of discontinuation (washout) of sutimlimab treatment on clinical endpoints. Patients with early termination (ET) were also invited for a follow-up visit 9 weeks after their last dose of sutimlimab. Data from these patients were included in the 9-week washout results and combined data are reported for the ET/SFU visit.

The study was conducted and the protocol, any amendments, and patient-informed consent were written according to the ethics principles derived from international ethics guidelines, including the Declaration of Helsinki and the International Council on Harmonisation guidelines for Good Clinical Practice, all applicable laws, rules, and regulations; and the study was approved by local independent ethics committees or review boards. All patients provided written informed consent for their participation in the study.

### Key outcomes and statistical analysis

Part B was conducted open label, and no formal statistical hypotheses were tested. Sample size considerations for Part A of the study have been published previously,^19^ whereas for Part B, no sample size analysis was conducted. Analyses of efficacy endpoints were descriptive. In general, continuous variables were summarised by descriptive statistics, including number, mean, standard deviation (SD) and standard error (SE), median, minimum, and maximum. Categorical and response variables were presented for the number and percentage in each category.

The efficacy assessments to evaluate disease activity in Part B included mean Hb levels and haemolytic parameters, including total bilirubin, lactate dehydrogenase (LDH), and haptoglobin; QoL assessments, as assessed by the Functional Assessment of Chronic Illness Therapy–Fatigue (FACIT-Fatigue) score; and transfusion requirements. Please note, the results of QoL assessments are reported in a separate publication. Data up to 1 year in Part B, last on-treatment visit with available assessment (LV), and “washout” data 9 weeks after the last dose of sutimlimab (ET/SFU) are reported here. Data up to week 79 or 87 are shown as they were the closest visit to the 1-year time point (26 weeks [Part A] + 52 weeks [1-year Part B]) where efficacy data were reported.

To assess safety, study investigators recorded incidence of treatment-emergent adverse events (TEAEs) and treatment-emergent serious adverse events (TESAEs); incidence of infections of ≥ grade 3 severity; and incidence of thromboembolic events.

### Role of the funding source

This study was funded by Sanofi. The study sponsor was involved in study design, in the collection, analysis, and interpretation of data, and in the writing of the report. All authors had access to primary clinical trial data, and had full editorial control of the manuscript.

## Results

### Baseline demographics and patient disposition

A total of 39 out of 42 patients enrolled in Part A, completed Part A and entered Part B. For patients enrolled in Part B, the baseline demographic and disease characteristics were generally similar between the patients who had received sutimlimab in Part A and continued sutimlimab treatment (sutimlimab-to-sutimlimab group) and patients who switched to sutimlimab in Part B (placebo-to-sutimlimab group) and were consistent with a patient population with CAD (Table 1). Patients enrolled in Part B were mostly female (79.5%) and had a median age (range) at baseline of 67 (46–88) years. The total mean (SD) baseline Hb and bilirubin levels were 9.3 (1.0) g/dL and 35.0 (11.5) μmol/L, respectively.

**Table 1:** Study population and disease characteristics at baseline.

Baseline^a^	Treatment arm in Part A		All patients (N = 39)
	Sutimlimab (n = 19)^e^	Placebo (n = 20)	
Mean (range) age, years	66 (46–88)	68 (51–83)	67 (46–88)
Female, n (%)	15 (78.9)	16 (80.0)	31 (79.5)
Geographic location, n (%)^b^	19	20	39
Europe	12 (63.2)	13 (65.0)	25 (64.1)
North America	3 (15.8)	3 (15.0)	6 (15.4)
Asia	3 (15.8)	2 (10.0)	5 (12.8)
Other	1 (5.3)	2 (10.0)	3 (7.7)
Hb g/dL, mean (SD)	9.2 (0.9)	9.3 (1.0)	9.3 (1.0)
Bilirubin μmol/L,^c^ mean (SD)	34.3 (11.5)	35.8 (11.8)	35.0 (11.5)
LDH U/L, mean (SD)	420.4 (206.4)	380.8 (243.1)	400.1 (223.9)
Reticulocyte count, 10^9^/L, mean (SD)	139.4 (65.9)	145.4 (44.8)	142.5 (55.4)
Haptoglobin g/L, mean (SD)	0.2 (0.0)	0.2 (0.0)	0.2 (0.0)
IgM, g/L, mean (SD)	4.7 (5.1)	2.7 (2.0)	3.7 (3.9)
Cold agglutinin titre at 4 °C, median (Min, Max)	1995 (158, 2,511,886)	1259 (40, 1,258,925)	1259 (316, 2,511,886)
Patients with transfusions in prior 1 year, n (%)	3.0 (15.8)	0.0 (0.0)	3.0 (7.7)
Patients with disabling circulatory symptoms, n (%)^d^	3.0 (15.8)	0.0 (0.0)	3.0 (7.7)
Acrocyanosis, n (%)	9.0 (47.4)	4.0 (20.0)	13.0 (33.3)
Raynaud's syndrome, n (%)	5 (26.3)	3.0 (15.0)	8.0 (20.5)
Patients with prior CAD therapy in past 5 years, n (%)	11 (57.9)	10 (50.0)	21 (53.9)
Patients with a history of ≥1 thromboembolic event, n (%)	1 (5.3)	1 (5.0)	2 (5.1)

CAD, cold agglutinin disease. Hb, haemoglobin. IgM, immunoglobulin M. LDH, lactate dehydrogenase. SD, standard deviation.

a Part A baseline values, for patients entering Part B.

b Europe includes France, Germany, Italy, Norway, and the United Kingdom. North America includes the United States. Asia includes Japan. Other includes Australia and Israel.

c Excluding patients with positive or unknown Gilbert's syndrome test result (sutimlimab n = 17, placebo n = 18, total N = 35).

d Reporting of disabling circulatory symptoms was based on the medical judgment of the investigators. The presence of disabling circulatory symptoms was collected at each visit in a dedicated questionnaire. If serious adverse event criteria were met (including severity grade 3), the event was additionally reported as a serious adverse event.

e Includes patients who continued into Part B of the study only (three patients withdrew from the study and did not continue into Part B—ie, one patient who had received sutimlimab in Part A and two patients who had received placebo in Part A).

A total of 32 out of 39 (82.1%) patients completed Part B (Supplementary Figure S2). Of these, 31 patients completed the 9-week SFU visit. The data from one participant who completed the full study treatment were not included in the final analysis set, because their SFU visit was conducted prematurely. Seven patients discontinued treatment in Part B and six patients with ET completed the 9-week follow-up visit. One patient with ET did not complete the 9-week SFU visit due to a fatal TESAE of squamous cell carcinoma of the lung, assessed by the investigator as not related to sutimlimab. The median (range) duration of sutimlimab treatment in Part B was 99 (22–177) weeks; 93 weeks (22–139) for the placebo-to-sutimlimab group, and 118 weeks (43–177) for the sutimlimab-to-sutimlimab group.

### Pharmacodynamic parameters (CP activity, CH50, C4, and C1q)

For patients who continued to Part B, the mean (SE) CP activity at baseline was 25.0% (4.5) in the sutimlimab group and 31.9% (6.2) in the placebo group. In Part A, the pre-dose CP activity at week 1 had decreased to 2.6% (0.6) after the first dose of sutimlimab, whereas the pre-dose level remained comparable to baseline in the placebo group (35.6% [5.8]). In Part B, by week 39, the mean (SE) pre-dose CP activity also decreased to 4.8% (1.27) in the placebo-to-sutimlimab group upon switching to sutimlimab, which was comparable with the week 39 mean (SE) pre-dose CP activity of those who received sutimlimab throughout Part A and continued on sutimlimab in Part B (3.8% [0.7]) (Supplementary Figure S3). Near-complete inhibition of CH50 was observed upon initiation of sutimlimab treatment and sustained in both groups throughout the Part B treatment period.

The mean (SE) C4 level at baseline was 0.1 (0.0) g/L, which was quickly restored to the normal range (0.2 to 0.5 g/L) in the sutimlimab group in Part A, with pre-dose levels of 0.2 (0.0) g/L 1 week after the first dose and remained within the normal range throughout Part B. C4 levels remained low (comparable to baseline levels) in the placebo group in Part A and were quickly restored to normal range (pre-dose level 0.3 [0.0] g/L) at week 39 upon switching to sutimlimab in the placebo-to-sutimlimab group during Part B (Supplementary Figure S3). C1q levels remained generally unchanged throughout the treatment period.

### Impact on anaemia

**Fig. 1:**
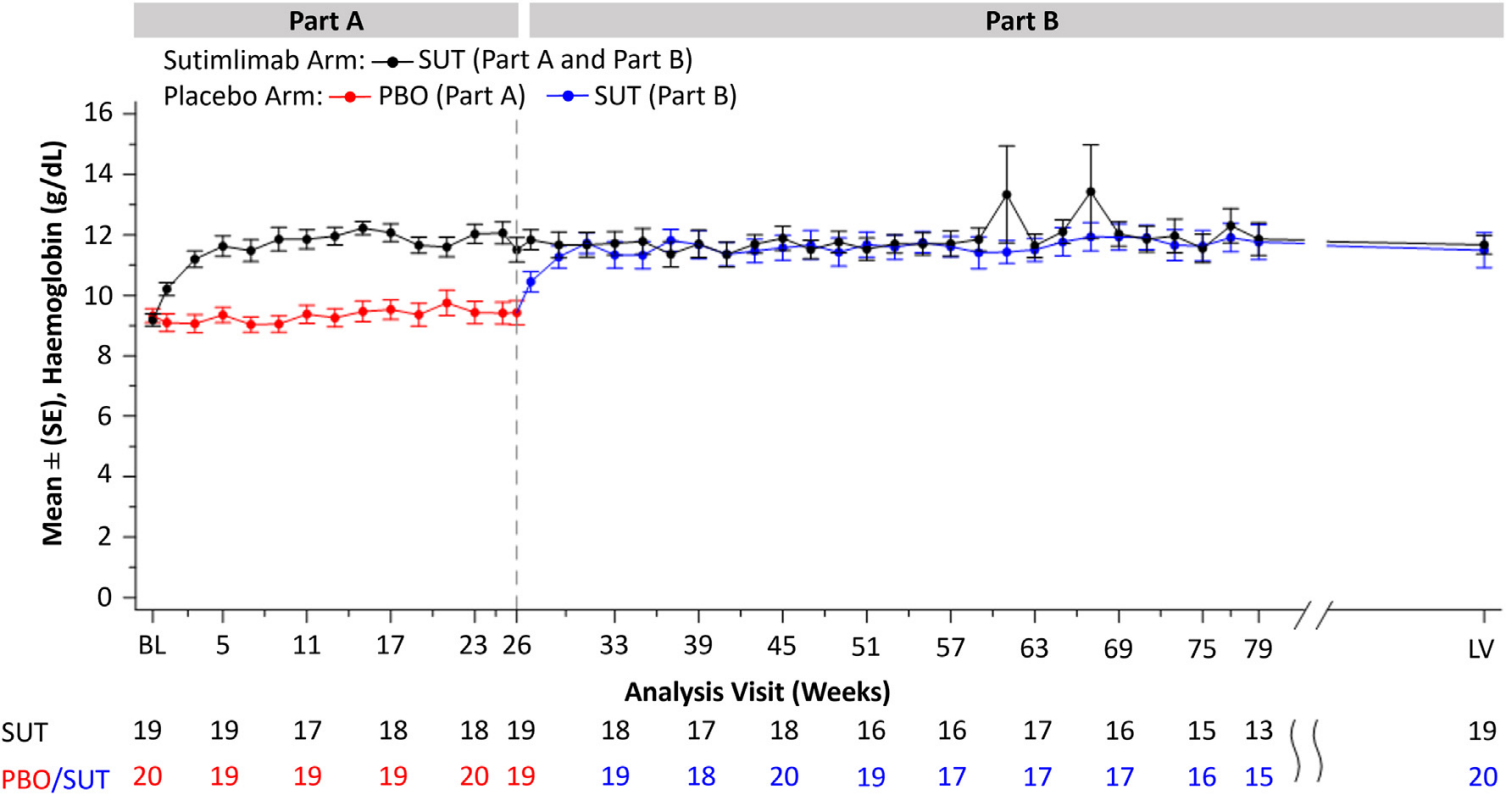
**Mean (SE) haemoglobin (Hb) level from baseline (BL) through to week 79, then last on-treatment visit with available assessment (LV)**. Only non-missing values are considered. LV is the last on-treatment visit with available assessment. Two data points in the sutimlimab-to-sutimlimab group at week 61 and 67 showed a greater mean (SE) level of Hb than at the other time points ie, 13.3 g/dL (1.6) and 13.4 g/dL (1.6). Both data points were attributed to the same patient. PBO, placebo. SE, standard error. SUT, sutimlimab.

Hb levels rapidly improved with sutimlimab treatment in Part A and were sustained through to LV in Part B (Fig. 1). The placebo-to-sutimlimab group saw rapid and comparable increases in Hb levels to those who had received sutimlimab in Part A and continued sutimlimab treatment. At week 26 (end of Part A), the mean (SE) level of Hb was 11.5 g/dL (0.4) in the sutimlimab group and 9.4 g/dL (0.4) in the placebo group. By week 31 the mean level (SE) of Hb in the placebo-to-sutimlimab group (11.7 g/dL [0.3]) had reached the level observed in the sutimlimab-to-sutimlimab group (11.7 g/dL [0.4]) and was mostly maintained at a comparable level ≥11.0 g/dL for both groups in Part B through to the end of treatment. At LV the mean (SE) level of Hb was 11.7 g/dL (0.3) in the sutimlimab-to-sutimlimab group and 11.5 g/dL (0.6) in the placebo-to-sutimlimab group.

### Impact on haemolysis

**Fig. 2:**
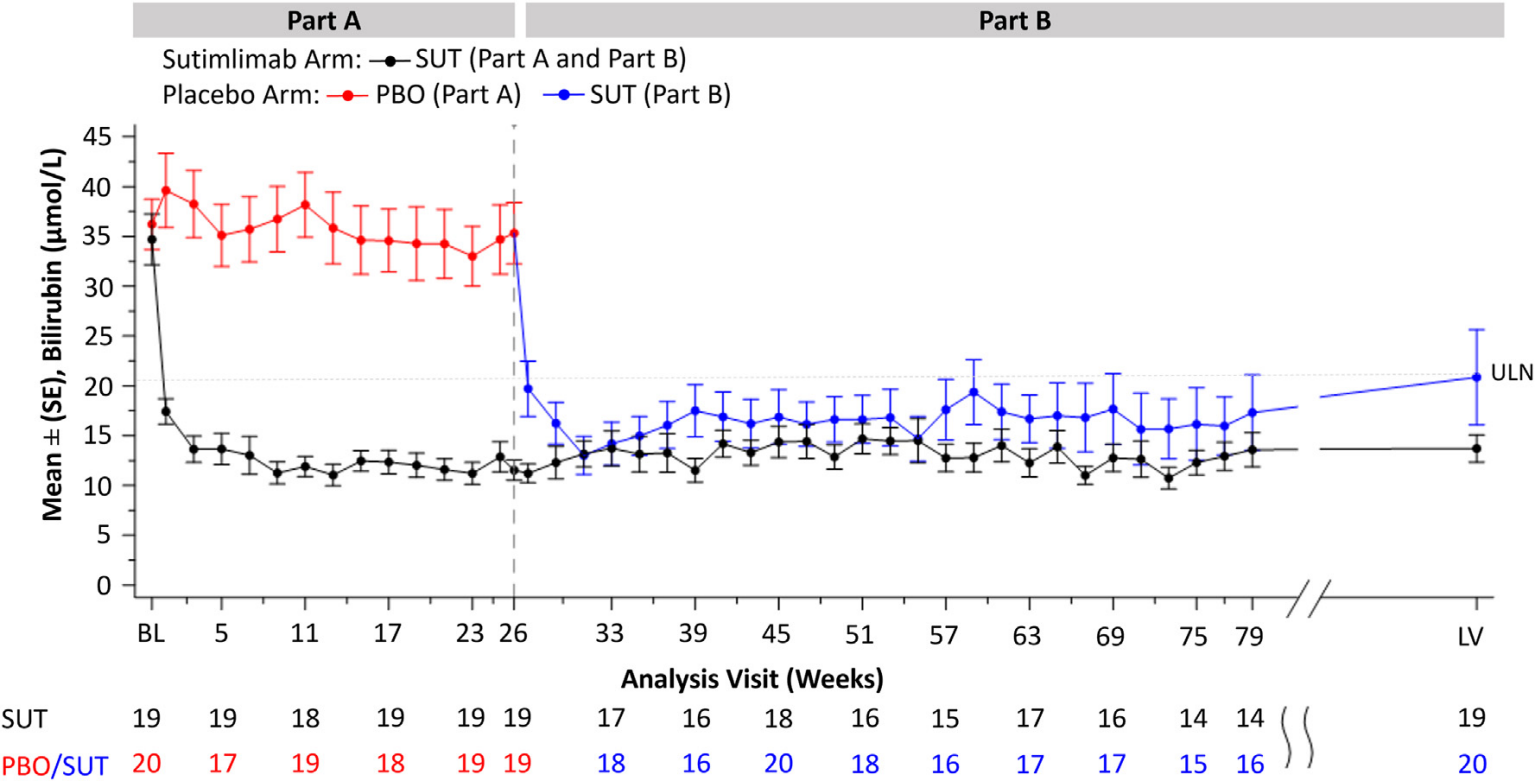
**Mean (SE) bilirubin**^**a**^**level from baseline (BL) through to week 79, then last on-treatment visit with available assessment (LV)**. Only non-missing values are considered. LV is the last on-treatment visit with available assessment. ^a^The central laboratory normal range for bilirubin was 5.1–20.5 μmol/L. PBO, placebo. SE, standard error. SUT, sutimlimab. ULN, upper limit of the normal range (20.5 μmol/L).

Mean total bilirubin was normalised with sutimlimab treatment in Part A and sustained through to LV in Part B (Fig. 2). At week 31, the mean (SE) bilirubin in the placebo-to-sutimlimab group (12.3 μmol/L [2.0]) had decreased to a similar level as in the sutimlimab-to-sutimlimab group (13.2 μmol/L [1.4]). Similar levels of bilirubin were maintained throughout the remainder of Part B until the end of treatment. The mean (SE) levels of bilirubin at LV were 20.5 μmol/L (5.0) and 13.7 μmol/L (1.5) in the placebo-to-sutimlimab group and sutimlimab-to-sutimlimab group, respectively.

**Fig. 3:**
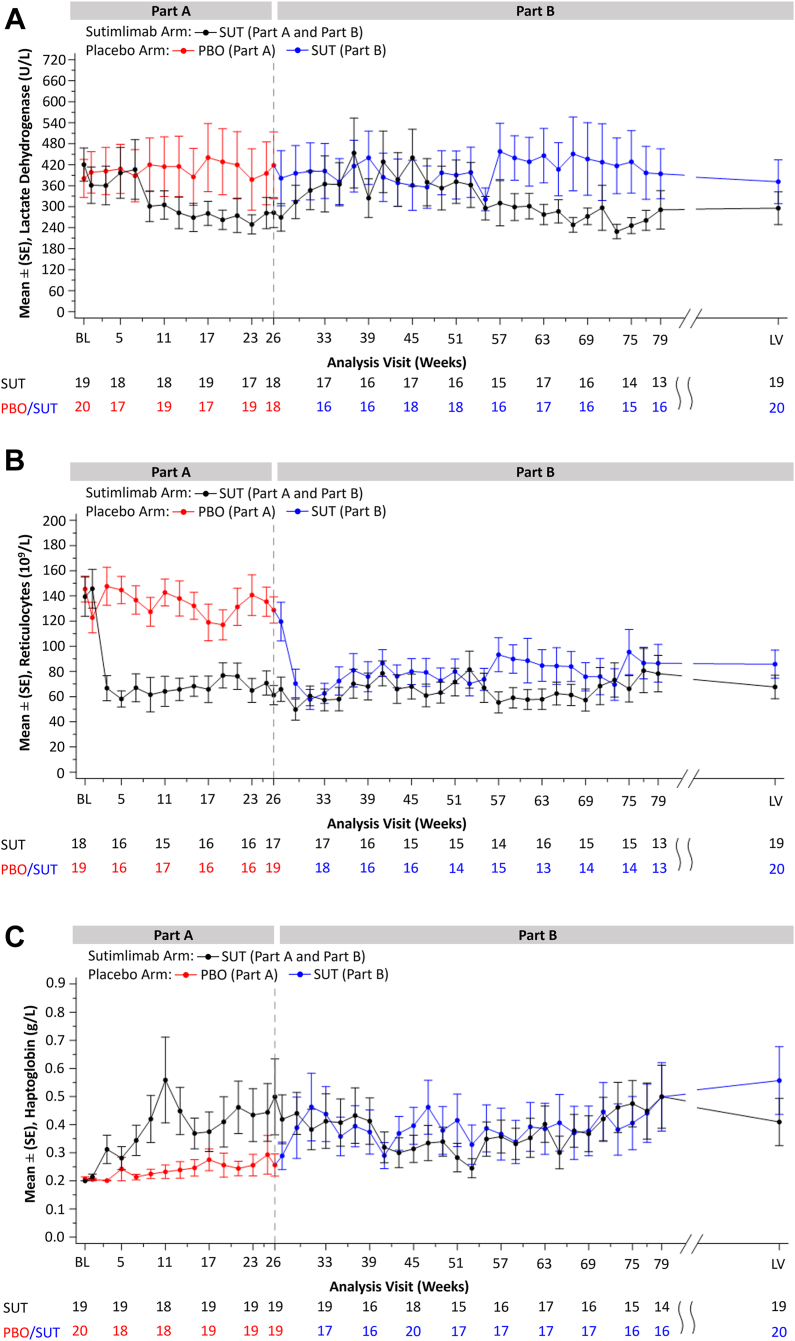
**Mean (SE) haemolytic markers from baseline (BL) to week 79, then last on-treatment visit with available assessment (LV). a) lactate dehydrogenase [LDH], b) reticulocyte count, c) haptoglobin**. Only non-missing values are considered. LV is the last on-treatment visit with available assessment. PBO, placebo. SE, standard error. SUT, sutimlimab.

Modest decreases in LDH, reductions in mean absolute reticulocyte count, and increases in haptoglobin levels observed in Part A with sutimlimab treatment, were maintained through to LV in Part B in the sutimlimab-to-sutimlimab group (Fig. 3). Upon switching to sutimlimab treatment in Part B, the placebo-to-sutimlimab group reached comparable levels of reticulocytes and haptoglobin as those who had previously received sutimlimab in Part A. Mean reticulocyte count and haptoglobin levels were maintained below 119.6 × 10^9^/L and above 0.3 g/L, respectively, in the placebo-to-sutimlimab group through end of treatment in Part B. There was no meaningful change from baseline observed in LDH levels in the placebo-to-sutimlimab group during most visits. Mean (SE) level of LDH at LV was 295.6 U/L (47.0) in the sutimlimab-to-sutimlimab group and 371.6 U/L (62.8) in the placebo-to-sutimlimab group.

### Transfusion data

A total of nine (23.1%) patients received ≥1 transfusion in Part B including six patients who received transfusions during the 9-week period following the last dose of sutimlimab. The three patients who received blood transfusions while on treatment had Hb levels <7.0 g/dL at the time of transfusion (Supplementary Figure S4; Supplementary Table S1). Two of the three patients who received transfusion during the treatment period in Part B did not complete the study; one due to a lack of efficacy (the patient's bilirubin was normalised during most of Part B of the study, indicating pharmacological action; however, the patient's Hb value did not normalise and remained ≤10.6 g/dL during the treatment period) and the other due to an adverse event (AE) of squamous cell carcinoma that resulted in death.

### Impact on quality of life

The QoL assessments for Part B, including FACIT-Fatigue scores, are reported in detail in a separate publication. In short, improvements in mean FACIT-Fatigue scores observed in Part A were sustained in Part B in patients previously treated with sutimlimab. Mean FACIT-Fatigue scores increased in the placebo-to-sutimlimab group to levels comparable to those who received sutimlimab in Part A. The mean levels in FACIT-Fatigue scores were similar between groups and were maintained throughout the remainder of Part B, although the mean FACIT-Fatigue scores of the placebo-to-sutimlimab group never fully reached the level of the sutimlimab-to-sutimlimab group.

### Safety

Throughout Part B of the CADENZA study, 36 of the 39 patients (92.3%) experienced a total of 395 TEAEs, and a total of seven patients experienced TESAEs (11 events) (Table 2). TEAEs reported by >10% of patients included fatigue (12 [30.8%] patients); anaemia (11 [28.2%] patients); arthralgia (8 [20.5%] patients); diarrhoea, hypertension, and nasopharyngitis (7 [17.9%] patients, each); asthenia, headache, and pyrexia (6 [15.4%] patients, each); dizziness, dyspnoea, fall, haemoglobinuria, nausea, and upper respiratory tract infection (5 [12.8%] patients each); and cyanosis (acrocyanosis), cystitis, gastroenteritis, insomnia, and iron deficiency anaemia (4 [10.3%] patients each). Of the 11 patients who reported anaemia during the study, seven patients reported the event during the 9-week follow-up period. TEAEs assessed by the investigator as related to sutimlimab were reported in 16 (41.0%) patients (58 events). The most frequently reported related TEAEs were headache (4 [10.3%] patients) and cyanosis (acrocyanosis), cystitis, fatigue, hypertension, injection site erythema, nausea, and pyrexia (2 [5.1%] patients each).

**Table 2:** Summary of safety results from Part B of the CADENZA study.

	Treatment arm in Part A		All patients^a^ (N = 39)
	Sutimlimab (n = 19)	Placebo (n = 20)	
**Number of TEAEs**	**117**	**90**	**395**
Patients with ≥1 TEAE, n (%)	18 (95.7)	20 (100.0)	36 (92.3)
Patients with ≥1 related TEAE,^b^ n (%)	6 (31.6)	4 (20.0)	16 (41.0)
**Number of TESAEs**	**2**	**3**	**11**
Patients with ≥1 TESAE, n (%)	2 (10.5)	1 (5.0)	7 (17.9)
Patients with ≥1 related TESAE,^c^ n (%)	1 (5.0)	0 (0.0)	1 (2.6)
Patients with ≥1 TESAE infection,^d^ n (%)	0 (0.0)	1 (5.0)	1 (2.6)
**Total number of TEAE thromboembolic events**	**1**	**0**	**2**
**Patients who discontinued treatment and/or study owing to a TEAE, n (%)**	**0 (0.0)**	**0 (0.0)**	**1 (2.6)**
**Deaths, n (%)**	**0 (0.0)**	**0 (0.0)**	**1 (2.6)**

AE, adverse event. TEAE, treatment-emergent adverse event. TESAE, treatment-emergent serious adverse event.

a The on-sutimlimab (treatment period) and off-sutimlimab (9-week washout period) safety data were analysed together according to the study protocol. The “all patients” column corresponds to the time period inclusive of both Part A and B (on-sutimlimab treatment period) plus the 9-week washout period (off-sutimlimab).

b Considered related to sutimlimab by the investigator. AEs with missing causality assessment were included in the related TEAEs/TESAEs; AEs with investigator causality assessment of “possible” or “probable” were considered related.

c TESAE of hypertension assessed as related to sutimlimab by the investigator.

d TEAE of infections are identified as TEAEs within the system organ class of infections and infestations.

Two (5.1%) patients with an underlying risk factor for thromboembolism experienced a thromboembolic event; one patient reported a transient ischaemic attack [TIA], and another patient reported an event of deep vein thrombosis [DVT]. The patient who experienced TIA had a medical history of two episodes of TIA and stroke, and the event occurred 175 days following initiation of sutimlimab. The patient who experienced DVT had a medical history of peripheral vascular disorder and reported the event following below-knee amputation of both lower limbs and amputation of fingers on both hands as part of treatment for extremity necrosis, which occurred in the context of Raynaud's phenomenon aggravation and multiple TEAEs of infection. The event occurred 288 days following initiation of sutimlimab. Both thromboembolic events were assessed as non-serious and not related to sutimlimab by the investigator. During the treatment period in Part B the bilirubin values of the two patients who experienced thromboembolic events were within the normal range of 5.1–20.5 μmol/L.

One (2.6%) patient experienced a TESAE of urinary tract infection. This patient had a history of benign prostatic hyperplasia and benign monoclonal hypergammaglobulinaemia and the event was assessed as not related to sutimlimab by the investigator. One TESAE of hypertension was assessed as related to sutimlimab by the investigator. This event was reported on day 146 during Part B of the study (the patient had previously received placebo during Part A). No meningococcal infections, serious events of hypersensitivity, anaphylaxis, or systemic lupus erythematosus were reported. One patient with a history of tobacco use experienced a TESAE of squamous cell carcinoma of the lung with fatal outcome. Sutimlimab was withdrawn due to this TESAE prior to the patient's death. This event was assessed as not related to sutimlimab by the investigator.

### Effects of discontinuations of sutimlimab treatment

**Fig. 4:**
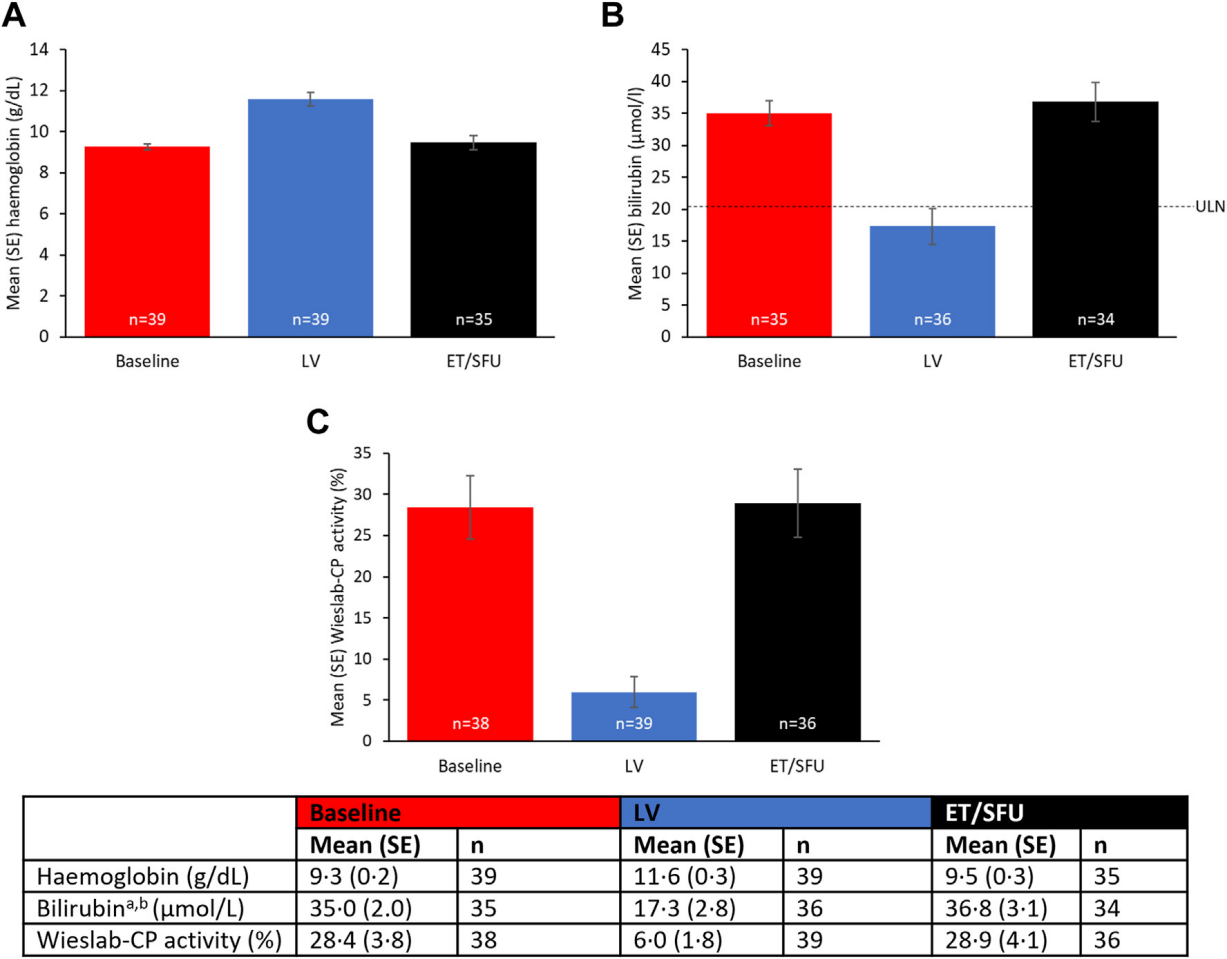
**Mean (SE) at baseline, last on-treatment visit with available assessment (LV [last non-missing value in Part B]), and at the 9-week early termination/safety follow-up (ET/SFU) in patients with CAD. a) haemoglobin; b) bilirubin**^**a,b**^**; and c) Wieslab-CP activity**. ^a^Bilirubin data excludes subjects with positive or unknown Gilbert's syndrome test result; ^b^The central laboratory normal range for bilirubin was 5.1–20.5 μmol/L. CAD, cold agglutinin disease. CP, complement pathway. ET/SFU, study follow-up visit 9 weeks after the last dose of sutimlimab. LV, last on-treatment visit with available assessment. SE, standard error. ULN, upper limit of normal.

At ET/SFU, 9 weeks after the last dose of sutimlimab, the near-complete inhibition of CP activity observed throughout the treatment period was reversed (Fig. 4). Similarly, the increased circulating levels of C4, the first substrate cleaved following activation of C1s, had comparatively decreased after discontinuation of sutimlimab.

After the 9-week washout, mean (SE) Hb decreased to 9.5 g/dL (0.3) from the LV value of 11.6 g/dL (0.3) in all patients and was comparable to the baseline value of 9.3 g/dL (0.2); mean (SE) total bilirubin for all patients increased to 36.8 μmol/L (3.1) from the end of Part B treatment and was comparable to the baseline value of 35.0 μmol/L (2.0); LDH, reticulocyte count, and haptoglobin values returned towards baseline levels in all patients (Fig. 4, see separate publication for FACIT-Fatigue results). Furthermore, six out of nine patients receiving transfusions in Part B received them after the last dose of sutimlimab (Supplementary Figure S4; Supplementary Table S1). Two of the six patients who received transfusions during the 9-week follow-up period following the cessation of sutimlimab had prematurely ended treatment, one due to a lack of efficacy (the patient had inconsistent improvement in Hb and sporadic normalisation of bilirubin) and one due to the sponsor's decision (the patient was not given the last scheduled dose as it would have fallen outside of the visit window). Both patients had Hb below the normal range at the time of treatment discontinuation.

During the 9-week washout period, 16 (41.0%) patients experienced a total of 55 AEs. One event was a TESAE of anaemia that was assessed by the investigator as not related to sutimlimab. Of the 11 patients who reported anaemia during Part B of the study, seven patients reported the event during the 9-week washout period.

Of the 55 AEs in the 9-week washout period, one event of grade 2 upper respiratory tract infection was assessed as related to sutimlimab by the investigator. The event was initially rated as grade 1 and not related on day 856 (21 days after the last dose of sutimlimab in the treatment phase). It then became grade 2 on day 879 (44 days after the last dose of sutimlimab) and was assessed by the investigator as related to sutimlimab.

There were no thromboembolic events during the 9-week washout period. Most AEs in the 9-week washout period could be attributed to worsening of underlying CAD including fatigue (10 [25.6%]) patients); anaemia (7 [17.9%] patients); dyspnoea (4 [10.3%] patients); asthenia and haemoglobinuria (3 [7.7%] patients, each); and Raynaud's phenomenon (1 [2.6%] patients).

## Discussion

Sutimlimab, a first-in-class humanised Ig monoclonal antibody that prevents CP activation has previously been shown to result in rapid and sustained improvements in Hb, haemolytic markers, and QoL in patients with CAD in Part A of the CADENZA and CARDINAL studies.^13^^,^^17^, ^18^, ^19^, ^20^ The final results from Part B of the phase 3 CADENZA study demonstrated that sustained treatment with sutimlimab continued to inhibit the classical CP resulting in inhibition of haemolysis, improved anaemia, and QoL for up to 1 year in patients with CAD, without a recent history of blood transfusion.

Improvements in Hb, normalisation of bilirubin, and reductions in mean absolute reticulocyte count, along with increases in haptoglobin levels were maintained throughout the treatment period, with those who had switched from placebo in Part A to sutimlimab in Part B reaching comparable levels to those who had received sutimlimab in Part A and continued to receive sutimlimab in Part B.

Bilirubin is recognised as a reliable biomarker for extravascular haemolysis, the predominant form of haemolytic activity in CAD.^12^ The bilirubin response (indicating inhibition of extravascular haemolysis) occurred within a week of the first dose of sutimlimab in the placebo-to-sutimlimab group reaching comparable levels to the sutimlimab-to-sutimlimab group by week 27, whereas the Hb levels reached comparable levels to the sutimlimab-to-sutimlimab group by week 31, confirming the bilirubin response to sutimlimab occurred more rapidly than the Hb response. This finding appears to be consistent with previous studies which showed rapid normalisation of bilirubin levels that were maintained below the upper limit of normal during sutimlimab treatment.^11^, ^12^, ^13^^,^^19^^,^^21^^,^^22^

The decrease in reticulocyte count coincided with the increased Hb level, whereas only modest reductions in LDH were observed in the sutimlimab-to-sutimlimab group and not the placebo-to-sutimlimab group in Part B of the study. Normalisation of LDH in responders who had substantially improved Hb levels may not have been possible in some cases due to remaining agglutination and associated RBC disruption, which sutimlimab would not affect.^11^^,^^23^

The mechanism of action of sutimlimab means that it affects the complement-mediated symptoms of CAD, such as haemolysis, anaemia, and fatigue, rather than the cold-induced, agglutination-mediated circulatory symptoms, such as acrocyanosis and Raynaud's phenomenon.^3^^,^^5^^,^^6^ However, there is no reason for circulatory adverse events to be attributed to sutimlimab based on its mechanism of action. Mild circulatory symptoms are frequently found to accompany CAD, although it is uncommon for them to be the driving indication for treatment.^24^ This is due to the fact that the majority of patients with CAD suffer from chronic haemolysis, caused by persistent activation of the classical CP, with mild or absent circulatory symptoms, which can often be adequately managed with thermal protection or cold avoidance alone.^24^^,^^25^ However, for the <10% of patients with severe circulatory symptoms with compensated haemolysis, complement-directed therapy may not be as beneficial.^25^

In Part B of the study, nine patients had an Hb level which met the criteria for a blood transfusion; however, per protocol, the analysis of Part B included the 9-week follow-up period following the cessation of sutimlimab, which meant that the transfusion data included the period during which patients were no longer receiving sutimlimab. Thus, although nine patients received transfusions in Part B, in fact, most of the patients (six out of nine) received them after the last dose of sutimlimab.

Improvements in the FACIT-Fatigue scores coincided with the improvement in Hb levels and were maintained throughout the treatment period in those who received sutimlimab treatment throughout the study. In the placebo-to-sutimlimab group, the improvements in FACIT-Fatigue were maintained from week 39. The QoL assessments for Part B are reported in detail in a separate publication.

In the 9-week follow-up period after cessation of sutimlimab, there was a decline in treatment effect, which was not unexpected. Assessment of pharmacodynamic markers showed the reversal of the near-complete inhibition of CP activity and return of CH50 and C4 towards baseline levels at the 9-week washout timepoint. The haemolytic markers also approached baseline values, indicating a return of haemolytic activity, and emphasising the need for continuous treatment with sutimlimab for continued inhibition of disease activity in CAD.

Continuous treatment with sutimlimab is needed to maintain favourable treatment effects, due to its mechanism of action, to inhibit CP activation.^11^ Treatment with therapies not approved for management of CAD, such as rituximab (a B-cell-directed therapy) monotherapy or rituximab in combination with cytostatic agents (eg, bendamustine or fludarabine) may be more time-limited and can result in remission.^11^^,^^26^ However, these unapproved therapies are often associated with delayed or inadequate responses (particularly with monotherapy) and toxicity issues (especially with combination treatment).^11^^,^^26^ It has been suggested that sutimlimab may be of particular value to patients who are severely affected by CAD and who need a short time to response (eg, during acute exacerbations that do not spontaneously resolve).^27^ Furthermore, it has been proposed that chronic inhibition of complement activity in CAD may offer clinical benefits beyond improving haemolysis and anaemia, such as decreasing risk of thromboembolic events, although more data are needed to support this hypothesis.^10^

In the consensus recommendations for AIHAs, published in 2020 (before the approval of sutimlimab), it was agreed by an expert panel that complement inhibition at various levels of the complement cascade (such as with eculizumab, terminal complement [C5] inhibitor) may be a useful treatment approach for CAD in emergencies or for later lines of treatment.^24^ It is expected that complement inhibitors, like sutimlimab (C1s inhibitor), which inhibit the complement cascade further upstream, may be more effective than eculizumab as they address extravascular haemolysis (the predominant form of haemolysis in CAD).^1^^,^^27^

Overall, sutimlimab was generally well tolerated by patients throughout the study. The type and frequency of TEAEs were generally consistent with the underlying disease indication, reported medical history, and an older, medically complex population; and most of the events experienced by the patients during the 9-week post-treatment follow-up period could be attributed to recurrence of CAD. Further real-world evidence for the long-term safety and efficacy of sutimlimab in CAD will be assessed via the CADENCE registry.^28^

CAD is a rare disease, and accordingly patient numbers in the CADENZA study are limited. The study had a reasonable sample size for a rare disease and was adequately powered to address the primary endpoint and key secondary endpoints, yet no formal statistical hypotheses could be tested in Part B and analyses were descriptive rather than inferential. In addition, as per the study protocol, the safety data and transfusion data analysis included TEAEs or transfusions that occurred with a start date on or after the Part B start date and included transfusions or AEs that occurred during the 9-week post-treatment follow-up period. Transfusions or AEs that occurred in the 9-week post-treatment follow-up period have been highlighted and summarised, and overall, the 9-week washout data were found to be supportive of the efficacy of sutimlimab. In this trial, it is not possible to say how quickly Hb levels decreased following cessation of sutimlimab. This is because assessment of Hb levels was only taken at a single 9-week washout timepoint following cessation of sutimlimab with no interim sampling to assess the kinetics of Hb decrease; however, a previous study indicated that in individual patients receiving lower doses of sutimlimab, a fall in Hb occurred within 2 weeks after sutimlimab discontinuation.^22^ Insufficient data are available to discuss the detailed effect of sutimlimab cessation on the components of the CP (eg, whether the patient's set of complement was complete at termination) or pharmacodynamics of sutimlimab clearance in the 9-week washout period. Patient and external factors may influence these kinetics and more frequent sampling within the 9 weeks' washout period would have been necessary to study these effects.

In conclusion, sustained treatment with sutimlimab continues to inhibit haemolysis and improve anaemia and QoL for up to 1 year. Responses were similar in patients administered sutimlimab throughout the study and those who switched to sutimlimab in Part B. Sutimlimab was generally well tolerated throughout the study. Continued inhibition of the classical CP via treatment with sutimlimab results in meaningful long-term benefits on haemolysis, and disease markers, including fatigue, regardless of transfusion history. Upon discontinuation of sutimlimab, treatment effect declined highlighting the importance of continuous sutimlimab therapy in patients with CAD.

## Contributors

All authors had access to primary clinical trial data, had full editorial control of the manuscript, and provided their final approval of all content.

## Data sharing statement

Qualified researchers may request access to patient-level data and related study documents, such as the clinical study report, study protocol (with amendments), statistical analysis plan, and dataset specifications. Of note, patient-level data will be anonymised, and study documents will be redacted to protect the privacy of trial participants. Further information related to Sanofi's data sharing criteria, eligible studies, and process for requesting access can be found at: https://vivli.org/.

## Declaration of interests

**AR** has received consultancy fees from Alexion Pharmaceuticals, Inc, Apellis Pharmaceuticals, Bioverativ, a Sanofi company, Novartis, Roche, and Sanofi; honoraria from Alexion, Amgen, Apellis, Novartis, Roche, Sanofi and Sobi, and advisory board fees from Alexion, Amgen, Apellis, Bioverativ, Novartis, Roche, Sanofi, and Sobi.

**SB** has received research support from Mundipharma; lecture honoraria from Apellis, Bioverativ (a Sanofi company), Janssen-Cilag, Momenta Pharmaceuticals and True North Therapeutics; and consultancy and advisory board honoraria from Apellis, Bioverativ, and Momenta Pharmaceuticals.

**WB** has received research support from Alexion; honoraria from Agios, Alexion, Apellis, Biocryst, Incyte, Janssen, Momenta, Novartis, Sanofi, and Sobi; and advisory board fees from Alexion, Novartis, Roche, Sanofi, and Sobi.

**SD** reports speaker fees and research funding from Janssen, BeiGene and Sanofi.

**BJ** has received reimbursement for travel costs related to scientific advice and scientific presentations from Sanofi.

**MM** has received research support for clinical studies from Roche; received fees from Amgen and GlaxoSmithKline for their participation in scientific advisory boards.

**ICW** has received consultancy fees from Alexion, Apellis, Novartis, and Biocryst; and honoraria from Alexion.

**MY** has no disclosures.

**JN** is a member of the advisory board for Chugai Pharmaceuticals and Alexion Pharmaceuticals and has received research funding and honorarium from Alexion Pharmaceuticals.

**JMIV** has received honoraria from Sanofi, Amgen, and BMS; research support from Beigene and AbbVie/Genmab, and advisory board fees from Sanofi and Janssen; all of these are institutional.

**JC** received research funding from Cerus, Kawasumi Laboratories and Sanofi; he also received speaker or advisory fees from Cerus, Fresenius Kabi, Grifols, MacoPharma, Pharm-Olam, Sanofi and Terumo Blood and Cell Technologies.

**CMB** has received research support from Alexion, Argenx, Electra, Novartis, and Sanofi; honoraria from Alexion, Argenx, and Sanofi; and advisory board fees from Argenx, Novartis, and Sanofi.

**DJ, FS, ML, MS, MW, NW, RY, SS** are Sanofi employees and may hold stock and/or stock options in the company.

## Appendix A Supplementary data

Supplementary Figures and Table

bivv009-04-16-1-1-cadenza-protocol-v8
