# Supplementary material for: Long-term efficacy and safety of continued complement C1s inhibition with sutimlimab in cold agglutinin disease: CADENZA study Part B
Source: eClinicalMedicine. 2024 Jul 18;74:102733. doi: 10.1016/j.eclinm.2024.102733 (PMC11293518; doi:10.1016/j.eclinm.2024.102733)
Supplement: Supplementary Figures and Table [file mmc1.pdf]

Long-term efficacy and safety of continued complement C1s inhibition with sutimlimab in cold agglutinin disease: CADENZA study Part B

Supplementary Appendix

Table of contents

Inclusion criteria 2
Exclusion criteria 3
Table S1: Narratives for nine patients who received transfusions in Part B and/or during the 9-week washout period 4
Figure S1: CADENZA study design 6
Figure S2. CONSORT flow-chart of patient disposition in CADENZA Part B 7
Figure S3: Mean (SE) pharmacodynamic markers from baseline (BL) through to week 75, then last on-treatment visit with available assessment (LV). (a) complement pathway (CP) activity and (b) C4 level 8
Figure S4: Individual haemoglobin (Hb) levels for nine patients who received transfusions in Part B 9

Inclusion criteria

- Adult male and female patients ≥18 years of age at screening
- Body weight of ≥39 kg at screening
- Confirmed diagnosis of primary cold agglutinin disease (CAD) based on the following criteria:

  a) Chronic haemolysis

  b) Polyspecific direct antiglobulin test (DAT) positive

  c) Monospecific DAT strongly positive for C3d

  d) Cold agglutinin titre ≥64 at 4˚C

  e) Immunoglobulin G DAT ≤1+, and

  f) No overt malignant disease
- Haemoglobin level ≤10·0 g/dL
- Bilirubin level above the normal reference range, including patients with Gilbert's syndrome
- Ferritin levels above the lower limit of normal. Concurrent treatment with iron supplementation was permitted if the patient had been on a stable dose during the previous 4 weeks
- Presence of one or more of the following CAD-related signs or symptoms within 3 months of screening:

  a) Symptomatic anaemia defined as:

    i. Fatigue

    ii. Weakness

    iii. Shortness of breath

    iv. Palpitations, fast heartbeat

    v. Light headedness and/or

    vi. Chest pain

    b) Acrocyanosis

    c) Raynaud's syndrome

  d) Haemoglobinuria

  e) Disabling circulatory symptoms, and/or

  f) Major adverse vascular event (including thrombosis)
- Bone marrow biopsy within 6 months of screening with no evidence of lymphoproliferative disease or haematological malignancy. An additional bone marrow biopsy was required if the prior bone marrow was deemed unsuitable for analysis by the sponsor
- Documented vaccinations against encapsulated bacterial pathogens (*Neisseria meningitidis*, including serogroup *B. meningococcus*, *Haemophilus influenzae*, where available, and *Streptococcus pneumoniae*) within 5 years of enrolment
- Patients had to be willing to receive transfusions if they met the eligibility criteria during the study treatment period. Patients who did not have a recent history of transfusion due to patient refusal or patient decision could not be enrolled if they did not agree to receive blood transfusions as needed
- Adequate intravenous access

- If female, had to be postmenopausal, surgically sterile, or have been established on (≥3 months prior to screening) and agreed to continue to use the same highly effective methods of birth control throughout the study and for 9 weeks following administration of the last dose of study drug
- Males had to be surgically sterile for at least 90 days or when sexually active with female partners of child-bearing potential agreed to use highly effective contraception from day 0 until 9 weeks following administration of the last dose of study drug
- Able to comprehend and give informed consent
- Able to comply with the requirements of the study and to complete the full sequence of protocol-related procedures

Exclusion criteria

Patients who met any of the following criteria were excluded from the study:

- Cold agglutinin syndrome secondary to infection, rheumatologic disease, or active haematologic malignancy
- History of blood transfusion within 6 months of screening, or history of more than one blood transfusion within 12 months of screening
- Clinically relevant infection of any kind within the month preceding enrolment (eg, active hepatitis C, pneumonia)
- Clinical diagnosis of systemic lupus erythematosus; or other autoimmune disorders with anti-nuclear antibodies at screening. Anti-nuclear antibodies of long-standing duration without associated clinical symptoms was to be adjudicated on a case-by-case basis during the confirmatory review of patient eligibility
- Positive hepatitis panel (including hepatitis B surface antigen and/or hepatitis C virus antibody) prior to or at screening
- Positive human immunodeficiency virus antibody at screening
- Treatment with rituximab monotherapy within 3 months or rituximab combination therapies (eg, with bendamustine, fludarabine, ibrutinib, or cytotoxic drugs) within 6 months prior to enrolment
- Concurrent treatment with corticosteroids other than a stable daily dose equivalent to ≤10 mg/day prednisone for previous 3 months
- Erythropoietin deficiency. Concurrent treatment with erythropoietin was permitted if the patient was on a stable dose for the previous 3 months
- Concurrent usage of iron supplementation unless the patient was on a stable dose for at least 4 weeks
- Clinically significant medical history or ongoing chronic illness that would jeopardise the safety of the patient or compromise the quality of the data derived from his/her participation in this study (as determined by the investigator [or designee]) at screening
- Concurrent treatment with other experimental drugs or participation in another clinical trial with any investigational drug within 30 days or 5 half-lives, whichever is greater, prior to treatment start
- Females who were pregnant, lactating, or, if having reproductive potential, were considered potentially unreliable with respect to contraceptive practice
- History of hypersensitivity to sutimlimab or any of its components

Table S1: **Narratives for nine patients who received transfusions in Part B and/or during the 9-week washout period.**

Patient	Study arm in Part A	Previously received transfusion in Part A (Y/N)	Part B patient narrative
**Transfusions in Part B treatment period**			
1	Sutimlimab	Y	In Part B, the study participant received blood transfusions on day 189 (week 27) and day 294 (week 41) as Hb was <7 g/dL and the study participant was asymptomatic.<br><br>The first transfusion was given a week after the patient completed Part A of the study due to bleeding following surgery, which compounded the Hb levels.
2	Placebo	N	In Part B, the study participant received 1 unit of blood transfusion on day 897 (week 127) as Hb was <7 g/dL and the study participant was asymptomatic. In Part B, Hb and bilirubin values normalised and stayed within normal range at most of the visits but beginning approximately at week 89, markers of anaemia and haemolysis began to deteriorate.<br><br>The study participant did not complete the treatment period and did not complete the follow-up period as the drug was withdrawn due to the AE of squamous cell carcinoma of the lung and the study participant died due to the same event.
3	Placebo	N	The study participant received 2 units of blood transfusion on day 561 (week 79) as the Hb was <7 g/dL and the study participant was asymptomatic.<br><br>The study participant did not complete Part B of the study due to lack of efficacy (bilirubin was normalised during most of Part B of the study; however, the patient's Hb value normalised only at a few instances) with the last dose of sutimlimab administered on day 561 (week 79).
**Transfusions in Part B 9-week follow-up period**			
4	Sutimlimab	N	The study treatment was prematurely discontinued due to lack of efficacy (the patient had inconsistent improvement in Hb and sporadic normalisation of haemolysis) with the last dose of sutimlimab administered on week 45 (day 316). There were no underlying clinical features identified to explain the incomplete response.<br><br>The study participant received 2 units of transfusion on day 371 (week 53, 8 weeks after the last dose) of Part B of the study as the Hb value was <9 g/dL and the study participant was symptomatic.
5	Sutimlimab	N	The study treatment was prematurely discontinued as the sponsor recommended the patient may not take the last dose of study drug outside the visit window. Therefore, the last dose of sutimlimab was administered on week 83 (day 575).<br><br>The study participant required 3 transfusions after the end of treatment.<br><br>After the end of treatment, the study participant received transfusion on day 599 (week 85, 2 weeks after the last dose), day 611 (week 87, 4 weeks after the last dose), and day 625 (week 89, 6 weeks after the last dose) as the Hb value was <9 g/dL and the study participant was symptomatic.
6	Sutimlimab	N	The study participant did not require transfusions throughout the 6-month treatment period and during the Part B treatment period.<br><br>At the SFU visit (day 674; week 97, approximately 9 weeks after last dose of sutimlimab), Hb decreased by 2·2 g/dL to 8·0 g/dL, and the study participant received 1 unit of transfusion as the Hb value was <9 g/dL and the study participant was symptomatic.
7	Placebo	Y	The study participant did not receive any transfusion during the treatment period in Part B of the study and did not miss any scheduled dose of sutimlimab.<br><br>The study participant received 2 units of blood transfusion on day 696 (week 99; ~8 weeks after the last dose) as the Hb was <9 g/dL and the study participant was symptomatic.
8	Placebo	N	The study participant did not receive any transfusion during the treatment period in Part B of the study and did not miss any scheduled dose of sutimlimab.

Patient	Study arm in Part A	Previously received transfusion in Part A (Y/N)	Part B patient narrative
			At the SFU visit (day 867; week 123, ~9 weeks after last dose), the study participant received 1 unit of blood transfusion (Hb was 9·1 g/dL, and the study participant was symptomatic with pre-syncope and short breathing).
9	Placebo	Y	The study participant did not require transfusions throughout the treatment period in Part B.<br><br>During the SFU period the study participant received 2 units of blood transfusion on day 1184 (week 169, ~6 weeks after the last dose) as the Hb was <9 g/dL and the study participant was symptomatic.

AE=adverse event. Hb=haemoglobin. N=no. SFU=safety follow-up. Y=yes.

Figure S1: **CADENZA study design**.

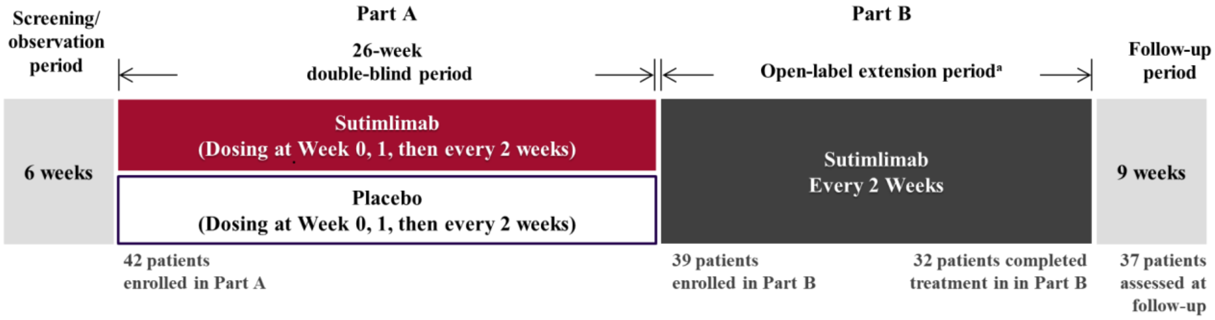

[a]The duration of the open-label extension was variable; unless a patient withdrew, the minimum time was 1 year from the last patient completing Part A, to a maximum time of approximately 2 years.

***Figure S2*. CONSORT flow-chart of patient disposition in CADENZA Part B.**

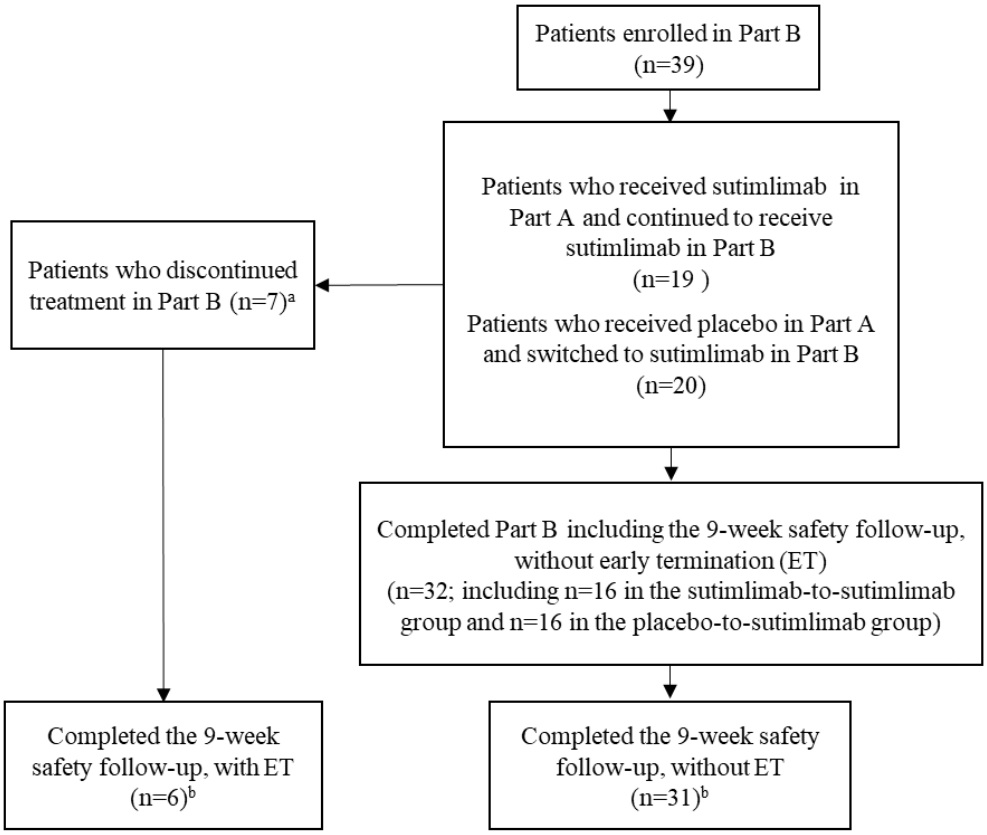

[a]Of the seven (17·9%) patients who discontinued sutimlimab, three (7·7%) patients, including one patient from the sutimlimab-to-sutimlimab group and two patients from the placebo-to-sutimlimab group, discontinued due to lack of efficacy. Two patients discontinued due to withdrawal of consent (one in the sutimlimab-to-sutimlimab group and one in the placebo-to-sutimlimab group). Another patient in the sutimlimab-to-sutimlimab group discontinued due to being unable to attend the end-of-treatment visit within the visit window and therefore did not receive one sutimlimab dose; and one patient in the placebo-to-sutimlimab group discontinued due to a fatal TESAE of squamous cell carcinoma of the lung that was assessed by the investigator as not related to sutimlimab.[b]One patient out of 32 who completed the full study treatment had their SFU visit prematurely (and therefore their data was not included in the final analysis because they did not meet the 9-week criteria). SFU=safety follow-up. TESAE=treatment-emergent serious adverse event.

***Figure S3*: Mean (SE) pharmacodynamic markers from baseline (BL) through to week 75, then last on-treatment visit with available assessment (LV). (a) complement pathway (CP) activity and (b) C4 level.**

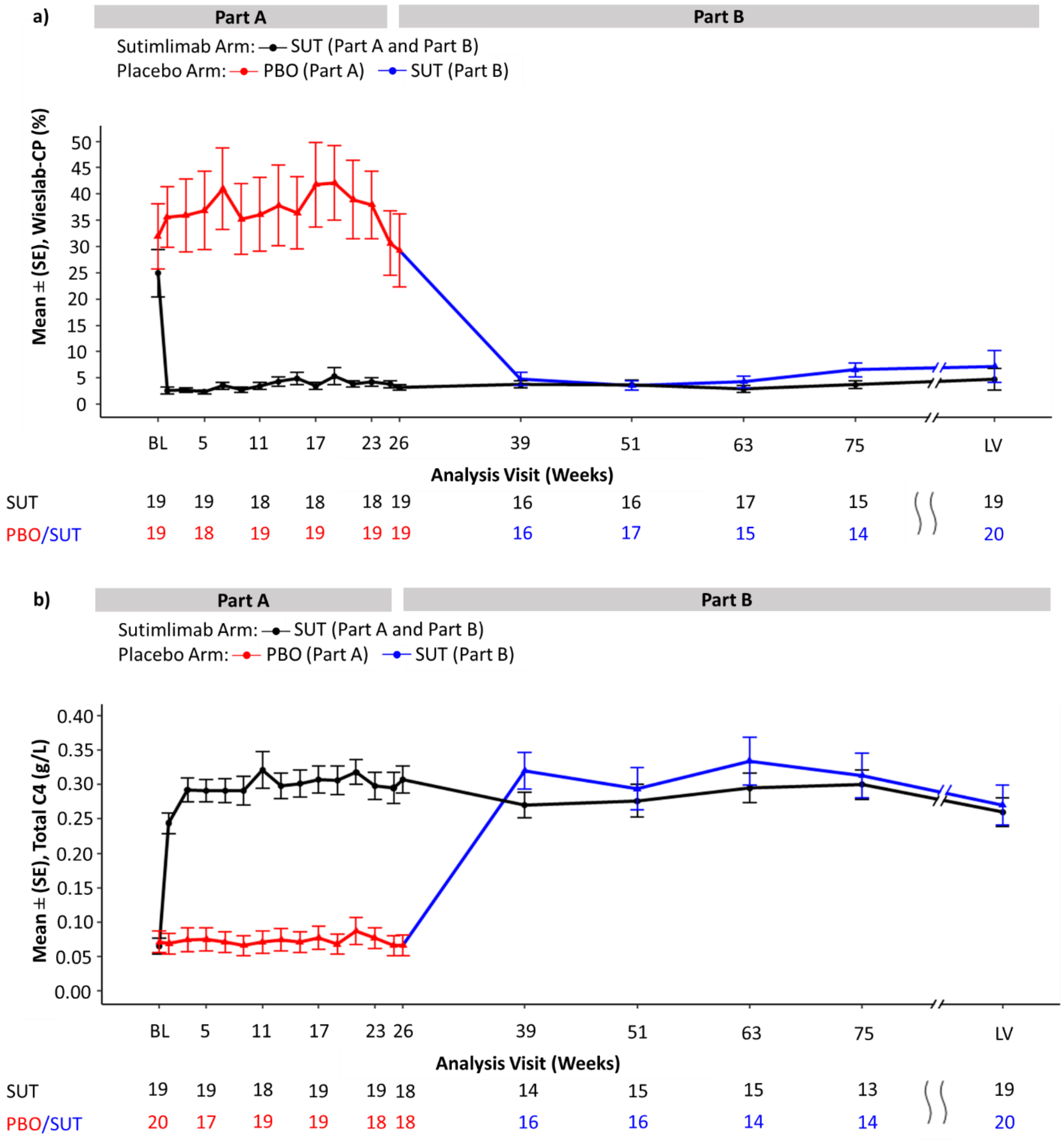

PBO=placebo. SE=standard error. SUT=sutimlimab.

***Figure S4*: Individual haemoglobin (Hb) levels for nine patients who received transfusions in Part B.**

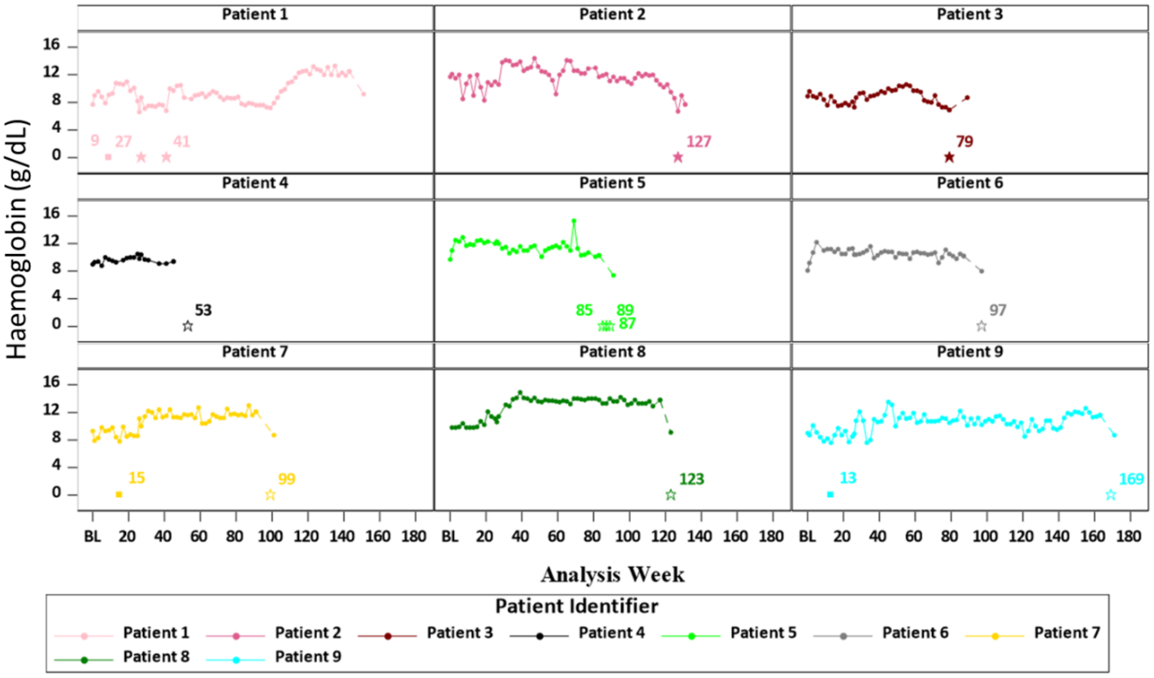

Dashed line represents post-treatment period. Filled square represents transfusions during Part A. Filled star represents transfusions on or before Part B treatment ends. Unfilled star represents transfusions post-treatment end. Only non-missing values are considered. All transfusions occurring after the Part A treatment start are included. For patient 4, transfusion occurs post-last visit of haemoglobin.
