# Supplementary material for: Long-term efficacy and safety of continued complement C1s inhibition with sutimlimab in cold agglutinin disease: CADENZA study Part B
Source: eClinicalMedicine. 2024 Jul 18;74:102733. doi: 10.1016/j.eclinm.2024.102733 (PMC11293518; doi:10.1016/j.eclinm.2024.102733)
Supplement: bivv009-04-16-1-1-cadenza-protocol-v8 [file mmc2.pdf]

Cadenza
STUDY

PROTOCOL BIVV009-04 (EFC16216)

A Phase 3, Randomized, Double-Blind, Placebo-Controlled Study to Assess the Efficacy and Safety of BIVV009 in Patients with Primary Cold Agglutinin Disease Without a Recent History of Blood Transfusion

IND #128190
EudraCT #2017-003539-12

Version 1: 24 August 2017
Version 1.1: 09 September 2017
Version 3: 21 March 2018
Version 4: 29 June 2018
Version 5: 19 July 2018
Version 6: 15 October 2019
Version 7: 07 July 2020
Version 8: 04 November 2020

Bioverativ USA, Inc.
225 Second Avenue
Waltham, MA 02451

This study will be performed in compliance with Good Clinical Practice.

CONFIDENTIAL
The information contained herein may not be used, disclosed, or published without the written consent of Bioverativ USA Inc.

PROTOCOL AMENDMENT SUMMARY OF CHANGES

DOCUMENT HISTORY

Document	Country/countries impacted by amendment	Date
Protocol Version 8	All	04 November 2020
Protocol Version 7	All	07 July 2020
Protocol Version 6	All	15 October 2019
Protocol Version 5	All	19 July 2018
Protocol Version 4	Not implemented	29 June 2018
Protocol Version 3.1	Japan	27 March 2018
Protocol Version 3	All, except Japan	21 March 2018
Protocol Version 2	Not implemented	NA
Protocol Version 1.6	Italy	29 January 2018
Protocol Version 1.5	Belgium	10 January 2018
Protocol Version 1.4	France	15 December 2017
Protocol Version 1.3	Norway	06 December 2017
Protocol Version 1.2	UK	29 November 2017
Protocol Version 1.1	All	09 September 2017
Protocol Version 1 (original)	All	24 August 2017

Present amendment (Version 8) to the protocol 04 November 2020

This is a global amendment to protocol Version 7 and is regarded as a substantial amendment based on the criteria set forth in Article 10(a) of Directive 2001/20/EC of the European Parliament and the Council of the European Union.

OVERALL RATIONALE FOR THE AMENDMENT

The primary reasons for this amendment to Protocol BIVV009-04 (Cadenza), Version 8 are:

- To include post-infusion vital signs assessment for patients who will receive undiluted infusions,
- To clarify that the hematology panel and Direct Antiglobulin Test (DAT) are performed at the site local laboratory and not at the central laboratory,
- To change for Part A the statistical method for the primary efficacy endpoint (responder status) in using the Cochran-Mantel-Haenszel (CMH) test instead of the Fisher's Exact test,
- To remove reference to the study procedural manual and replace it with the specific manuals.

PROTOCOL AMENDMENT SUMMARY OF CHANGES

Section # and name	Description of change	Brief rationale
Title Page, Approval of the Protocol, Protocol Amendment Summary of Changes Table	Document formatting revisions.	The change was made to reflect the update of the amended protocol version 8 and include a previous protocol version.
Section 1 Synopsis: Safety Outcome Measures	"Post-infusion vital signs and" were added to the list of endpoints for the patients receiving undiluted infusions.	The changes were made in relation to the inclusion of post-infusion vital signs assessment for the subset of patients receiving undiluted infusions of the IMP.
Section 1 Synopsis: Statistical Methods	Regarding the primary endpoint (responder status) analysis for Part A, the Fisher's Exact test was replaced by "Cochran-Mantel-Haenszel (CMH) test".	The primary efficacy analysis method was changed and will use the Cochran–Mantel–Haenszel (CMH) test instead of the Fisher's exact test to apply a statistically more powerful method with regard to the small sample size setting, and to ensure consistency in primary analysis and sensitivity analyses for the primary endpoints.
Section 4.3.5 Safety Endpoints	"Post infusion vital signs and" were added to the list of endpoints for the patients receiving undiluted infusions.	The changes were made in relation to the inclusion of post-infusion vital signs assessment for the subset of patients receiving undiluted infusions of the IMP.
Section 6.1 Schedule of Study Procedures: Table 3, footnote "l"	The following text was added:<br>"or for post-infusion vital signs in case of undiluted administration, after each undiluted infusion".	The changes were made in relation to the inclusion of post-infusion vital signs for patients receiving undiluted infusions of the IMP for a subset of patients.
Section 6.1.1.1 Screening assessments (initiating at Day -42)	The following text in the Bone Marrow Biopsy was removed:<br>"in the study procedural manual" and replaced by: "in Appendix A (Bone Marrow Biopsy Report Review Process) to the 'Subject Eligibility Review Process Overview'".	As study procedural manual was not created, references to this document across the protocol were either removed or replaced by references to the applicable study manuals.
Section 6.1.1.3 Confirmatory review of patient eligibility	The following text was removed: "forms and the adjudication process may be found in the study procedural manual" and replaced by "process may be found in the 'Subject Eligibility Process Overview'".	As study procedural manual was not created, references to this document across the protocol were either removed or replaced by references to the applicable study manuals.
Section 6.1.5.6 Infusion of the Undiluted Study Drug	This following text was added: "Post-infusion vital signs will be collected for these patients".	The changes were made in relation to the inclusion of post-infusion vital signs assessment for the subset of patients receiving undiluted infusions of the IMP.
Section 6.2.2 Patient number and identification	The following text was removed: "Details on patient number assignments will be available in the study procedural manual."	As study procedural manual was not created, references to this document across the protocol were either removed or replaced by references to the applicable study manuals.

Section # and name	Description of change	Brief rationale
Section 6.2.3 Randomization	The following text was modified:<br>Details on the randomization process are will be available in the study procedural manual eCRF Completion Guidelines.	As study procedural manual was not created, references to this document across the protocol were either removed or replaced by references to the applicable study manuals.
Section 6.2.5 Blinding	The following text was removed:<br>"Further details on blinding procedures will be provided in the study procedural manual".	As study procedural manual was not created, references to this document across the protocol were either removed or replaced by references to the applicable study manuals.
Section 6.3.7 Clinical Laboratory Evaluations	The following text was removed:<br>"For efficacy evaluations results from the central laboratory (when collected and available) will be recorded in the eCRF and utilized for analyses."	As the hematology panel tests are performed at the site local laboratory, the reference to the central laboratory was removed from this section.
Section 7.1 Description of Objectives and Endpoints	The following text was modified:<br>"For endpoints involving laboratory parameters, except for hematology panel, results from the central laboratory will be used. Local laboratory values were utilized for Direct Antiglobulin Test (DAT) for, among others, eligibility purposes and hematology panel that allows both determination of eligibility and the medical management of the patient, with assessment of the hemoglobin criteria necessitating blood transfusions."	As the hematology panel tests and Direct Antiglobulin Test (DAT) are performed at the site local laboratory, this section was modified to be consistent with what is done in the study.
Section 7.3.2 Methods of analysis	The following text was removed: "Fisher's Exact test" and replaced with: "Cochran-Mantel-Haenszel test".	The primary efficacy analysis method was changed from Fisher's exact test to a stratified Cochran–Mantel–Haenszel test, to apply a statistically more powerful method in the small sample size setting, and to ensure result consistency between primary analysis and sensitivity analyses for the primary endpoints.
Investigator Agreement	Document formatting revisions.	The change was made to reflect the update of the amended protocol version 8.
Appendix A Clinical Laboratory Evaluations	The following text was added to Appendix A:<br>"Detailed information on the allocation of samples for central or local laboratory processing is available in the Laboratory Manual."	As the hematology panel tests are performed at the site local laboratory, this section was modified to be consistent with what is done in the study.
Appendix B CI-CTC Version 4.03 (CTCAE V4.03)	Hyperlink replacement.	The change was made to replace obsolete hyperlink with active hyperlink to CTCAE web page.
Appendix J Protocol Amendment History	Document formatting revisions.	The change was made to include the summary of changes introduced with the previous amendment.

TABLE OF CONTENTS

PROTOCOL BIVV009-04 (EFC16216) 1

PROTOCOL AMENDMENT SUMMARY OF CHANGES 2

TABLE OF CONTENTS 5

LIST OF TABLES 9

LIST OF ABBREVIATIONS 10

1 SYNOPSIS 12

2 INTRODUCTION 20

2.1 PHARMACOLOGY OF BIVV009 20

2.2 BACKGROUND AND STUDY RATIONALE 20

2.2.1 Non-clinical experience 22

2.2.2 Clinical experience 23

2.2.3 Pharmacokinetic experience and dose justification 24

2.2.4 Potential risks and benefits 24

3 STUDY OBJECTIVES 27

3.1 PRIMARY OBJECTIVE (PART A) 27

3.2 SECONDARY OBJECTIVES (PART A) 27

3.3 PRIMARY OBJECTIVE (PART B) 27

3.4 SECONDARY OBJECTIVE (PART B) 27

3.5 EXPLORATORY OBJECTIVE (PART B) 28

4 INVESTIGATIONAL PLAN 29

4.1 STUDY DESIGN 29

4.2 DISCUSSION OF STUDY DESIGN 31

4.3 STUDY ENDPOINTS 32

4.3.1 Primary endpoint (Part A) 32

4.3.2 Secondary efficacy endpoints (Part A) 32

4.3.3 Exploratory efficacy endpoints (Part A) 32

4.3.4 Efficacy endpoints (Part B) 33

4.3.5 Safety endpoints 33

4.3.6 Pharmacokinetic endpoints 34

4.3.7 Pharmacodynamic endpoints 34
4.3.8 Immunogenicity endpoints 35

4.4 DURATION OF THE STUDY 35

4.5 END OF STUDY 35

5 PATIENT SELECTION 36

5.1 INCLUSION CRITERIA 36

5.2 EXCLUSION CRITERIA 37

5.3 REMOVAL OF PATIENTS FROM STUDY PARTICIPATION, AND STUDY SUSPENSION AND STOPPING RULES 38

6 STUDY PROCEDURES 40

6.1 SCHEDULE OF STUDY PROCEDURES 40

6.1.1 Screening/observation period (Days -42 to Day -1) 45
6.1.1.1 Screening assessments (initiating at Day -42) 45
6.1.1.2 Interim visits during screening/observation period 47
6.1.1.3 Confirmatory review of patient eligibility 47

6.1.2 Day 0 visit (first dose) 47

6.1.3 Weeks 1-25 Part-A 49

6.1.4 End-of-treatment visit in Part A (Week 26) 50

6.1.5 Part B extension phase 50
6.1.5.1 Procedures to be performed every 2 weeks 50
6.1.5.2 Procedure to be performed every 4 weeks 51
6.1.5.3 Procedures to be performed every 3 months 51
6.1.5.4 Additional PK, PD, and ADA sampling 52
6.1.5.5 Infusion of the study drug at patient’s home 52
6.1.5.6 Infusion of the undiluted study drug 52
6.1.5.7 Early termination/end of study/safety follow-up visit 52

6.2 STUDY TREATMENT 53

6.2.1 Drug supplies and accountability 53

6.2.2 Patient number and identification 54

6.2.3 Randomization 54

6.2.4 Dose preparation and administration 54

6.2.5 Blinding 55

6.2.6 Concomitant medications 55

6.2.7 Contraception 56

6.3 STUDY ASSESSMENTS 57

6.3.1 Demographic data 57

6.3.2 Medical history 57
6.3.3 Height, body weight, and vital signs 57
6.3.4 Electrocardiograms 57
6.3.5 Physical examinations 58
6.3.6 Immunization review 58
6.3.7 Clinical laboratory evaluations 58
6.3.8 Additional testing in case of hypersensitivity/allergic reaction 58

6.4 PHARMACOKINETIC PROCEDURES 59
6.4.1 Pharmacokinetic blood sample collection and processing 59
6.4.2 Analytical methodology 59

6.5 PHARMACODYNAMIC PROCEDURES 59
6.5.1 Pharmacodynamic and ADA blood sample collection and processing 59
6.5.2 Analytical methodology 59

6.6 SAFETY PROCEDURES 59

6.7 ADVERSE EVENTS AND LABORATORY ABNORMALITIES 60
6.7.1 Definition of adverse events 60
6.7.1.1 Definition of adverse drug reactions 60
6.7.1.2 Definition of serious adverse events 61
6.7.1.3 Definition of unexpected adverse event/SUSAR 62
6.7.2 Clinical adverse events 62
6.7.3 Laboratory test abnormalities 64
6.7.4 Serious adverse event reporting - procedures for investigators 64
6.7.5 Follow-up of adverse events 64
6.7.6 Follow-up of abnormal laboratory results 65
6.7.7 Pregnancy 65
6.7.8 Overdose 65

7 DATA ANALYSES AND SAMPLE SIZE 66

7.1 DESCRIPTION OF OBJECTIVES AND ENDPOINTS 66

7.2 DEMOGRAPHICS AND BASELINE DISEASE CHARACTERISTICS 66

7.3 EFFICACY - PART A 66
7.3.1 Analysis populations 66
7.3.2 Methods of analysis 67

7.4 EFFICACY - PART B 68
7.4.1 Analysis populations 68

7.4.2 Methods of analysis ... 68

7.5 PHARMACOKINETICS ... 68

7.5.1 Analysis population ... 68

7.5.2 Methods of analysis ... 68

7.6 PHARMACODYNAMICS ... 69

7.6.1 Analysis population ... 69

7.6.2 Methods of analysis ... 69

7.7 IMMUNOGENICITY ... 69

7.7.1 Analysis population ... 69

7.7.2 Methods of analysis ... 69

7.8 SAFETY ... 69

7.8.1 Analysis population ... 69

7.8.2 Methods of analysis ... 70

7.9 INTERIM ANALYSES ... 70

7.10 SAMPLE SIZE CONSIDERATIONS ... 70

7.11 DATA HANDLING AND RECORD KEEPING ... 70

7.12 QUALITY CONTROL AND QUALITY ASSURANCE ... 71

8 ADMINISTRATIVE ASPECTS ... 72

8.1 CHANGE IN PROTOCOL ... 72

8.2 SITE INITIATION VISIT/INVESTIGATOR MEETING ... 72

8.3 DISCLOSURE ... 72

8.4 MONITORING ... 72

8.5 ETHICAL ASPECTS ... 73

8.5.1 Declaration of helsinki/good clinical practice ... 73

8.5.2 Patient information and informed consent ... 73

8.6 INDEPENDENT ETHICS COMMITTEE/INSTITUTIONAL REVIEW BOARD AND REGULATORY AUTHORITIES ... 74

8.7 RECORDS ... 74

9 REFERENCES ... 76

INVESTIGATOR AGREEMENT ... 78

10 APPENDICES ... 79

APPENDIX A CLINICAL LABORATORY EVALUATIONS 80

APPENDIX B CI-CTC VERSION 4.03 (CTCAE V4.03) 81

APPENDIX C FUNCTIONAL ASSESSMENT OF CHRONIC ILLNESS THERAPY (FACIT)-FATIGUE SCALE 82

APPENDIX D EQ-5D-5L 83

APPENDIX E 12-ITEM SHORT FORM SURVEY (SF-12) 86

APPENDIX F PATIENT’S GLOBAL IMPRESSION OF CHANGE (PGIC) 90

APPENDIX G HEALTHCARE RESOURCE UTILIZATION 91

APPENDIX H PATIENT’S GLOBAL IMPRESSION OF [FATIGUE] SEVERITY 92

APPENDIX I VACCINE SCHEDULE FOR PATIENTS IN JAPAN ONLY 93

APPENDIX J PROTOCOL AMENDMENT HISTORY 94

APPENDIX K COUNTRY SPECIFIC REQUIREMENT – HOME INFUSIONS WITH BIVV009 103

APPENDIX L HOME INFUSION PATIENT SATISFACTION SURVEY 110

LIST OF TABLES

Table 1 - Transfusion criteria 29

Table 2 - Responder definition 30

Table 3 - Study schedule of events 41

Table 4 - Study schedule of events (Part B) for patients with home infusions after Day 273 104

LIST OF ABBREVIATIONS

ADA	antidrug antibody
ADL	activities of daily living
AE	adverse event
AMR	antibody-mediated rejection
ANCOVA	analysis of covariance
AUC	area under the concentration-time curve
BLA	Biologics License Application
BP	bullous pemphigoid
CAgD	cold agglutinin disease
CFR	Code of Federal Regulations
CI	confidence interval
CIC	circulating immune complex
C_{max}	maximum observed concentration
CP	complement classical pathway
CRO	Clinical Research Organization
CTCAE	Common Terminology Criteria for Adverse Events
DAT	direct antiglobulin test
DNA	deoxyribonucleic acid
dsDNA	double-stranded DNA
EC	Ethics Committee
ECG	electrocardiogram
eCRF	electronic Case Report Form
EDC	electronic data capture
EOS	end of study
EOT	end of treatment
EQ-5D-5L	five level EuroQol – five dimensions questionnaire
ET	early termination
FACIT	Functional Assessment of Chronic Illness Therapy
FAS	Full Analysis Set
FAX	facsimile
FDA	Food and Drug Administration
FIH	first in human
GCP	Good Clinical Practice
Hgb	Hemoglobin
HIV	human immunodeficiency virus
IB	Investigator’s Brochure
ICF	informed consent form
ICH	International Council for Harmonisation
IM	Intramuscularly
IRB	Institutional Review Board
ITT	Intent-to-Treat
IUD	intrauterine device
IV	intravenous
IWRS	Interactive Web-based System

LDH	lactate dehydrogenase
LPO	last patient out
MAA	Market Authorization Application
mAb	monoclonal antibody
MedDRA	Medical Dictionary for Regulatory Activities
NCI	National Cancer Institute
NHP	non-human primate
NHV	normal healthy volunteer
NOAEL	no observed adverse effect level
PD	pharmacodynamic(s)
PGIC	Patient's Global Impression of Change
PGIS	Patient's Global Impression of [Fatigue] Severity
PI	Principal Investigator
PK	pharmacokinetic(s)
PP	per-protocol
QOL	quality of life
SAE	serious adverse event
SAP	Statistical Analysis Plan
SD	standard deviation
SF-12	12-Item Short Form Survey
SLE	systemic lupus erythematosus
SOP	Standard Operating Procedure
SUSAR	suspected unexpected serious adverse reaction
TEAE	treatment-emergent adverse event
USA	United States of America
WAIHA	warm autoimmune hemolytic anemia

1 SYNOPSIS

**Title of Study:**	Cadenza Study: A Phase 3, Randomized, Double-Blind, Placebo-Controlled Study to Assess the Efficacy and Safety of BIVV009 in Patients with Primary Cold Agglutinin Disease Without a Recent History of Blood Transfusion
**Overview of Study Design:**	This is a randomized, double-blind, placebo-controlled, multicenter study in patients with primary cold agglutinin disease (CAgD) without a recent history of blood transfusion. Eligible patients will receive study drug (BIVV009 or placebo) and undergo safety and efficacy assessments for 6 months (26 weeks) during Part A.<br>Following completion of the initial 6-month treatment period (Part A), patients will roll into the open-label long-term safety and durability of response extension phase (Part B) during which they will receive BIVV009.<br>For the purpose of marketing authorization application, an interim analysis of safety and efficacy data will be performed after all patients have completed the double-blind treatment period (Part A). The Part B open-label extension study will run for 1 year following last patient out (LPO) under Part A.
**Objectives:**	**Part A**<br>Primary Objective:<br>• The primary objective of Part A is to determine whether BIVV009 administration results in a ≥1.5 g/dL increase in hemoglobin (Hgb) level and avoidance of transfusion in patients with primary CAgD without a recent history of blood transfusion<br>Secondary Objectives:<br>Efficacy:<br>• To assess the effect of BIVV009 on clinical events and laboratory parameters related to hemolysis and anemia in patients with primary CAgD<br>• To assess the effect of BIVV009 on specific complications of CAgD (acrocyanosis, Raynaud's syndrome, hemoglobinuria, and thromboembolism)<br>• To assess the effect of BIVV009 on quality of life (QOL) in patients with primary CAgD<br>Safety:<br>• To evaluate the overall safety and tolerability of BIVV009 in patients with primary CAgD<br>Exploratory:<br>• To evaluate the effect of BIVV009 on certain disease-related biomarkers in patients with primary CAgD<br>• To evaluate the pharmacokinetics of BIVV009<br>• To evaluate the immunogenicity of BIVV009<br>**Part B**<br>Primary Objective:<br>• The primary objective of Part B is to evaluate the long-term safety and tolerability of BIVV009 in patients with primary CAgD.<br>Secondary Objective:<br>• The secondary objective of Part B is to investigate the durability of response during long-term treatment with BIVV009 in patients with primary CAgD.<br>Exploratory Objective:<br>• To describe the safety and patient satisfaction with the convenience of home infusions with BIVV009 in a subset of patients<br>• To describe the safety of undiluted infusions with BIVV009<br>• To evaluate the immunogenicity of BIVV009

Methodology/Study Design:

A

This randomized, double-blind, placebo-controlled study is designed to evaluate the efficacy, safety, and tolerability of BIVV009 in symptomatic patients with the complement-mediated disorder primary CAgD who do not have a recent history of blood transfusion.

During the 6-week Screening/Observation Period, prospective patients will have a detailed medical history documented (including all available transfusion history), physical evaluations, and blood samples collected for characterization of CAgD biomarkers, including Hgb levels on 3 occasions approximately every 2 weeks.

Patients under screening for Cadenza who require a transfusion(s) during the Screening/ Observation Period prior to the first study drug infusion (if medically indicated per the Investigator's discretion and within the parameters of the protocol specified transfusion criteria, see Table 1) will remain eligible. If a patient receives a transfusion(s) during the Screening/Observation Period prior to the first study drug infusion, the baseline visit (and first infusion of study drug) must occur at least 7 days following the transfusion.

Part A

The study will enroll approximately 40 primary CAgD patients who do not have a recent history of blood transfusion (ie, ≤1 transfusion during the last year and no transfusion during the last 6 months prior to enrollment). Eligible patients should have been diagnosed with primary CAgD at least 6 months prior to enrollment and should have had no history of transfusion during this period.

Eligible patients will be randomized 1:1 to receive an intravenous (IV) infusion of BIVV009 or placebo over approximately 60 minutes on Day 0, Day 7, and every 14 days thereafter through Week 25 (ie, Days 21, 35, 49, 63, 77, 91, 105, 119, 133, 147, 161, and 175). Patients who miss a dose (ie, outside the dosing window or >17 days since last dose) should return to the site for an unscheduled visit to receive another loading dose prior to their next scheduled visit. Patients will have an End-of-Treatment (EOT) visit in Part A on Day 182 (Week 26).

Patients who meet the transfusion criteria in Table 1 during the 6-month double-blind treatment period will receive a transfusion. Patients who receive a transfusion during Part A will not be withdrawn from the study and will be eligible to participate in Part B.

Table 1: Transfusion criteria

A patient will receive a transfusion during Part A or Part B if his or her Hgb level meets either of the following criteria:<br>• Hgb is <9 g/dL and the patient is symptomatic, *or*<br>• Hgb is <7 g/dL and the patient is asymptomatic

A responder analysis will be conducted following completion of the EOT visit at Week 26. The responder definition is provided in Table 2.

Table 2: Responder definition

A patient will be considered a responder in Part A if:<br>• Hgb increases ≥1.5 g/dL from baseline (defined as the last Hgb value before administration of the first dose of study drug) at treatment assessment endpoint (defined as mean value from Weeks 23, 25, and 26) and<br>• The patient did not receive a blood transfusion from Week 5 through Week 26 (EOT), and<br>• The patient did not receive treatment for CAgD beyond what is permitted per protocol from Week 5 through Week 26 (EOT)

B	A list of excluded concomitant medications, as well as allowed concomitant medications with restrictions, is provided in the protocol. Beyond the permitted concomitant medications, study drug, and transfusions, patients may receive no other therapies for the treatment of CAgD while enrolled in Part A of this study; patients requiring other treatment for their CAgD in Part A will be withdrawn from the study and counted as non-responders. These patients will not be eligible to participate in Part B.<br>Part B<br>Following completion of dosing in the initial 6-month treatment period, patients will roll into the long-term safety and durability of response extension phase and receive BIVV009 in an open-label manner. Part B will run for 1 year following LPO under Part A. Patients requiring treatment with permitted concomitant medications and/or transfusions will not be discontinued from the study. Patients in Part B will be transfused per the transfusion criteria in Table 1. Patients who receive a transfusion in Part B will not be withdrawn from the study.<br>Blinding will be maintained when rolling patients into the extension period by providing a crossover dose at Week 26 to allow placebo patients to receive the BIVV009 loading dose at start of BIVV009 dosing. Patients who were randomized to BIVV009 during the 6-month treatment period will receive a placebo dose at Week 26 to maintain blinding.<br>All patients will then continue to receive BIVV009 dosing every 2 weeks starting at Week 27. Should patients deviate from their scheduled dosing, a repeat loading dose may be required. On-site visits will be completed ~ every 3 months (at a minimum) for collection of pharmacodynamic (PD) and pharmacokinetic (PK) samples, ADA samples and additional safety and efficacy measures. PK, PD and anti-drug antibodies (ADAs) samples will be collected 9 weeks after administration of the last dose of study drug in patients who discontinue early, as well as in patients who experience a hematological breakthrough event.<br>The study will be complete 12 months following LPO for Part A at which time all patients receiving on-going treatment will proceed to an End-of-Study (EOS) visit.
**Number of Patients:**	Approximately 40 male and/or female patients ≥18 years of age who have a confirmed diagnosis of primary CAgD and who do not have a recent history of blood transfusion will be randomized.
**Number of Study Sites:**	Approximately 55 sites worldwide will be targeted for participation to identify approximately 40 eligible primary CAgD patients.
**Main Criteria for Inclusion:**	All patients must meet all the following inclusion criteria to be enrolled:<br>I 01. Adult male and female patients ≥18 years of age at Screening<br>I 02. Body weight of ≥39 kg at Screening<br>I 03. Confirmed diagnosis of primary CAgD based on the following criteria:<br>a) Chronic hemolysis<br>b) Polyspecific direct antiglobulin test (DAT) positive<br>c) Monospecific DAT strongly positive for C3d<br>d) Cold agglutinin titer ≥64 at 4°C<br>e) IgG DAT ≤1+, and<br>f) No overt malignant disease<br>I 04. Hemoglobin level ≤10.0 g/dL<br>I 05. Bilirubin level above the normal reference range, including patients with Gilbert's Syndrome<br>I 06. Ferritin levels above the lower limit of normal. Concurrent treatment with iron supplementation is permitted if the patient has been on a stable dose during the previous 4 weeks.<br>I 07. Presence of one or more of the following CAgD-related signs or symptoms within 3 months of Screening:<br>a) Symptomatic anemia defined as:

	i. Fatigue<br>ii. Weakness<br>iii. Shortness of breath<br>iv. Palpitations, fast heart beat<br>v. Light headedness, and/or<br>vi. Chest pain<br>b) Acrocyanosis<br>c) Raynaud's syndrome<br>d) Hemoglobinuria<br>e) Disabling circulatory symptoms, and/or<br>f) Major adverse vascular event (including thrombosis)<br>I 08. Bone marrow biopsy within 6 months of Screening with no overt evidence of lymphoproliferative disease or other hematological malignancy. An additional bone marrow biopsy will be required if the prior bone marrow is deemed unsuitable for analysis by the Sponsor.<br>I 09. Documented vaccinations against encapsulated bacterial pathogens (*Neisseria meningitis*, including serogroup B *meningococcus*, where available, *Haemophilus influenzae*, and *Streptococcus pneumoniae*) within 5 years of enrollment or as specified in Section 6.1.1.1.<br>I 10. Patients must be willing to receive transfusions if they meet the eligibility criteria during the study treatment period. Patients who do not have a recent history of transfusion due to patient refusal or patient decision should not be enrolled if they do not agree to receive blood transfusions as needed.<br>I 11. Adequate IV access<br>I 12. If female, must be post-menopausal, surgically sterile, or be established on (≥3 months prior to Screening) and agree to continue to use the same highly effective methods of birth control throughout the study and for 9 weeks following administration of the last dose of study drug<br>I 13. Males must be surgically sterile for at least 90 days or when sexually active with female partners of child-bearing potential will agree to use highly effective contraception from Day 0 until 9 weeks following administration of the last dose of study drug.<br>I 14. Able to comprehend and give informed consent<br>I 15. Able to comply with the requirements of the study and to complete the full sequence of protocol-related procedures
**Exclusion Criteria:**	Patients who meet any of the following criteria will be excluded from the study:<br>E 01. Cold agglutinin syndrome secondary to infection, rheumatologic disease, or active hematologic malignancy<br>E 02. History of blood transfusion within 6 months of screening or history of more than one blood transfusion within 12 months of screening<br>E 03. Clinically relevant infection of any kind within the month preceding enrollment (eg, active hepatitis C, pneumonia)<br>E 04. Clinical diagnosis of systemic lupus erythematosus (SLE); or other autoimmune disorders with anti-nuclear antibodies at Screening. Anti-nuclear antibodies of long-standing duration without associated clinical symptoms will be adjudicated on a case-by-case basis during the Confirmatory Review of Patient Eligibility (Section 6.1.1.3).<br>E 05. Positive hepatitis panel (including hepatitis B surface antigen and/or hepatitis C virus antibody) prior to or at Screening<br>E 06. Positive human immunodeficiency virus (HIV) antibody at Screening

<table>
<tr><td></td><td>E 07. Treatment with rituximab monotherapy within 3 months or rituximab combination therapies (eg, with bendamustine, fludarabine, ibrutinib, or cytotoxic drugs) within 6 months prior to enrollment
E 08. Concurrent treatment with corticosteroids other than a stable daily dose equivalent to ≤10 mg/day prednisone for previous 3 months
E 09. Erythropoietin deficiency. Concurrent treatment with erythropoietin is permitted if the patient has been on a stable dose for the previous 3 months.
E 10. Concurrent usage of iron supplementation unless the patient has been on a stable dose for at least 4 weeks.
E 11. Clinically significant medical history or ongoing chronic illness that would jeopardize the safety of the patient or compromise the quality of the data derived from his/her participation in this study (as determined by the Investigator [or designee]) at Screening
E 12. Concurrent treatment with other experimental drugs or participation in another clinical trial with any investigational drug within 30 days or 5 half-lives, whichever is greater, prior to treatment start
E 13. Females who are pregnant, lactating, or, if having reproductive potential, are considered potentially unreliable with respect to contraceptive practice
E 14. History of hypersensitivity to BIVV009 or any of its components.</td></tr>
<tr><td>Test Product(s), Dose, and Mode of Administration:</td><td>Study drug will be administered over approximately 60 minutes by IV infusion in accordance with the Pharmacy Manual. Patients with underlying cardiopulmonary disease may receive a 2-hour infusion with Sponsor approval. At pre-selected countries/sites and at certain visits during Part B administration of the study drug may be provided in the form of home infusions (Appendix K). In Part B, administration of the study drug may be provided in the form of undiluted solution to a subset of patients.
Patients will be randomized to receive 1 of the following treatments:
Test Product: Patients who weigh less than 75 kg will receive fixed doses of 6.5 g of BIVV009. Patients who weigh 75 kg or more will receive fixed doses of 7.5 g of BIVV009.
Placebo Control: Sterile saline solution for IV infusion-based formulation buffer.</td></tr>
<tr><td>Duration of Treatment:</td><td>
<table>
<tr><td>Part A</td><td>Screening/Observation Period:</td><td>6 weeks (Days -42 through Day -1)</td></tr>
<tr><td></td><td>Treatment Period:</td><td>25 weeks (Day 0 through Day 175)</td></tr>
<tr><td></td><td>End-of-Treatment (EOT) visit:</td><td>Week 26: 1 week after administration of the last dose of study drug in Part A (Day 182)</td></tr>
<tr><td>Part B</td><td>Safety and Durability of Response Extension Phase:</td><td>Crossover loading dose at Week 26; bi-weekly dosing starting at Week 27 and continuing for 1 year after LPO in Part A</td></tr>
<tr><td>Part A/B</td><td>Early Termination (ET) visit/ Safety Follow-up visit/EOS visit:</td><td>9 weeks after last dose of study drug administration</td></tr>
</table>
Patients who complete Part A per protocol through the EOT visit will participate in Part B, the long-term safety and durability of response extension phase of the study.</td></tr>
<tr><td>Efficacy Endpoints and Outcome Measures:</td><td><u>Part A</u>
Primary efficacy endpoint:
The primary efficacy endpoint is the responder rate as defined in Table 2.
Secondary efficacy endpoints:
• Mean change from baseline in Hgb at treatment assessment endpoint (mean of values at Week 23, 25, and 26)</td></tr>
</table>

	• Mean change from baseline in bilirubin (excluding patients with Gilbert's Syndrome) at treatment assessment endpoint<br>• Mean change from baseline in QOL, as assessed by the change in Functional Assessment of Chronic Illness Therapy (FACIT)-Fatigue scale scores at treatment assessment endpoint<br>• Mean change from baseline in lactate dehydrogenase (LDH) at treatment assessment endpoint<br>• Incidence of solicited symptomatic anemia at EOT<br>**Exploratory efficacy endpoints:**<br>• Mean change from baseline in QOL, as assessed by the change in the five level EuroQol – five dimensions questionnaire (EQ-5D-5L) scores at treatment assessment endpoint<br>• Mean change from baseline in QOL, as assessed by the change in the 12-Item Short Form Survey (SF-12®) at the end of treatment assessment endpoint<br>• Proportion of patients with ≥12 g/dL Hgb at treatment assessment endpoint<br>• Incidence of thromboembolic events after the first 5 weeks of study drug administration<br>• Median time to normalization of bilirubin<br>• Median time to normalization of LDH<br>• Median time to normalization of haptoglobin<br>• Median time to Hgb of ≥12 g/dL<br>• Proportion of patients normalizing haptoglobin at treatment assessment endpoint<br>• Proportion of patients normalizing bilirubin at treatment assessment endpoint<br>• Proportion of patients with abnormal LDH at baseline who normalize LDH at treatment assessment endpoint<br>• Patient's Global Impression of Change (PGIC) to assess patient's perception of changes in CAgD disease burden at EOT<br>• Patient's Global Impression of [Fatigue] Severity (PGIS) to assess patient's perception of changes in fatigue at EOT<br>• Incidence of disabling circulatory symptoms at treatment assessment endpoint<br>• Total healthcare resource utilization at EOT (Part A)<br>Part B<br>The following parameters of disease activity will be assessed:<br>• Hemoglobin<br>• Bilirubin (total)<br>• QOL assessments (FACIT-fatigue, EQ-5D-5L, SF-12, PGIS, and PGIC)<br>• LDH<br>• Transfusion requirements<br>• Haptoglobin<br>• Total healthcare resource utilization at EOT<br>• Satisfaction with home infusion after first home infusion and after fourth home infusion will be assessed in patients with home infusions (Appendix K, Appendix L)

**Safety Outcome Measures:**	Safety assessments for this study include adverse events (AEs), serious AEs (SAEs), clinical laboratory evaluations, SLE panel, vital sign measurements, electrocardiograms (ECGs), physical examination findings, and serum disease-related biomarkers.<br>In addition, the following will be assessed for safety evaluation:<br>• Hemolytic breakthrough (rapid fall in Hgb ≥2 g/dL associated with an increase in LDH/bilirubin and/or decrease in haptoglobin since the last scheduled visit) through the EOT at Week 26<br>• Infections of ≥ Grade 3 severity (ie, requiring IV antibiotics)<br>• Thromboembolic events<br>• For patients with home infusions, safety assessments will additionally include AEs with onset within 24 hours of the infusion<br>• For patients receiving undiluted infusions, safety assessments will additionally include post-infusion vital signs and AEs with onset within 24 hours of the infusion
**Pharmacokinetic Outcome Measures:**	Pharmacokinetic endpoints will include:<br>• Plasma concentrations of BIVV009<br>• PK parameters. Appropriate exposure parameters (Cmax, AUC) will be derived using a population PK approach.<br>During Part A, PK blood samples will be collected at predose and 1 hour postdose (ie, 1 hour after completion of study drug infusion) from all patients on Days 0, 7, 21, 35, 49, 63, 77, 91, 105, 119, 133, 147, 161, and 175. An additional blood sample for PK analysis will be collected during the EOT visit on Day 182 or at ET if a patient withdraws early.<br>During Part B, PK samples will continue to be collected at predose and 1 hour (±15 minutes) postdose on Days 189, 217, and 245, then routinely at 3-month intervals starting at Day 273 through the remainder of the study. Samples will also be collected if a patient experiences a hematologic breakthrough event or withdraws from the study.
**Pharmacodynamic Outcome Measures:**	PD Primary Outcome Measure:<br>• Wieslab-CP<br>Exploratory Complement System Measures:<br>• CH50<br>• Total C4<br>• C1q<br>• C1s<br>During Part A, PD blood samples will be collected at predose and 1 hour postdose (ie, 1 hour after completion of study drug infusion) from all patients on Days 0, 7, 21, 35, 49, 63, 77, 91, 105, 119, 133, 147, 161, and 175. An additional blood sample for PD analysis will be collected during the EOT visit on Day 182 or at ET if a patient withdraws early.<br>During Part B, PD samples will continue to be collected at predose and 1 hour (±15 minutes) postdose on Days 189, 217, and 245, then routinely at 3-month intervals starting at Day 273 through the remainder of the study. Samples will also be collected if a patient experiences a hematologic breakthrough event or withdraws from the study.
**Immunogenicity Outcome Measures:**	Immunogenicity Outcome Measures will include pre-existing ADA and treatment-emergent ADA.<br>During Part A, samples will be collected at predose, on Day 0, 7, 35, 77, 133, 175 and on Day 182 (EOT) or at ET if a patient withdraws early. During Part B, ADA samples will continue to be collected at predose, on Days 189, 217, and 245, then routinely at 3-month intervals starting at Day 273 through the remainder of the study and at Safety Follow up visit 9 weeks after last dose. Samples will also be collected if a patient experiences a hematologic breakthrough or withdraws from the study.

**Sample Size:**	With 40 patients (20 per group), there is 87% power to detect a statistically significant difference of 50% between the BIVV009 group and placebo group if the true response rates are 85% and 35% for the BIVV009 and placebo groups, respectively. This calculation assumes a 2-sided 5%-level test comparing the response rates between the BIVV009 and placebo groups. A 50% improvement over placebo is considered clinically relevant.
**Statistical Methods:**	Interim analysis:<br>For the purpose of regulatory submission, an interim analysis of safety and efficacy data will be performed after all patients have completed Part A.<br>Analyses:<br>The efficacy endpoints will be presented for both the ITT and PP populations. All the per-protocol analyses will be considered supportive analyses. All hypothesis tests will be performed at a two-sided 0.05 significance level.<br>For Part A, the primary efficacy endpoint (responder status) will be compared between treatment groups using Cochran-Mantel-Haenszel (CMH) test for the ITT population, consisting of all patients who received at least 1 dose of study drug.<br>Secondary endpoints, defined as follows, will be tested only when the primary endpoint is statistically significant:<br>1. Mean change from baseline in Hgb at the treatment assessment endpoint (mean of values at Week 23, 25, and 26)<br>2. Mean change from baseline in bilirubin (excluding patients with Gilbert's Syndrome) at the treatment assessment endpoint<br>3. Mean change from baseline in QOL, as assessed by the change in FACIT-fatigue scale scores at the treatment assessment endpoint<br>4. Mean change from baseline in LDH at the treatment assessment endpoint<br>5. Incidence of solicited symptomatic anemia at EOT<br>The change from baseline variables will be analyzed using analysis of covariance (ANCOVA) with baseline as covariate. A multiple testing procedure will be specified in the statistical analysis plan to control the overall Type I error rate among the secondary endpoints.<br>For Part B, the analyses will be performed for all patients who received at least 1 dose of study drug in the extension period. All efficacy endpoints will be summarized by descriptive statistics and graphically (where appropriate) by prior treatment group in Part A. All safety endpoints will be summarized for all patients.<br>Further details regarding other endpoints and proposed analyses will be described in full in the Statistical Analysis Plans (SAP) for Part A and Part B.

2 INTRODUCTION

2.1 PHARMACOLOGY OF BIVV009

BIVV009 is a humanized monoclonal antibody (mAb) directed against human complement factor C1s, which along with C1r and C1q is a part of the C1 complex that sits at the apex of the complement classical pathway (CP). By binding C1s BIVV009 prevents the enzymatic action of the C1 complex on its substrates, complement factors C4 and C2, and thereby blocks formation of the C3 convertase. Note that this site of action of BIVV009 lies above the level of C3, which is the junction of all three pathways of complement activation. This is important to the specificity of the mechanism of action of BIVV009 because it means that the two other complement pathways, the alternative pathway and the lectin pathway, remain functionally intact for the purpose of host defense in the presence of BIVV009. Note, too, that the non-enzymatic role of C1q is left intact by BIVV009; this may be particularly relevant because of the importance of the pro-phagocytic “housekeeping” functions of the complement system including removal of apoptotic cells.

BIVV009 binds with high affinity and specificity to C1s of humans and non-human primates (NHPs). It has no affinity for the related proteases of the lectin pathway, MASP-1, and MASP-2. As discussed above, BIVV009 has disease-relevant inhibitory activity against CP in a variety of human in vitro models of human disease (including cold agglutinin disease [CAgD], bullous pemphigoid [BP], and warm autoimmune hemolytic anemia [WAIHA]). BIVV009 exerts its effect on the CP in a characteristic two-state behavior: complete inhibition at concentrations ≥20 μg/mL, and no inhibition at concentrations <20 μg/mL, with an abrupt transition between the two. This behavior means that graded degrees of inhibition are not readily measurable and that increasing BIVV009 concentration to ≥20 μg /mL results not in greater degrees of inhibition, but only in longer duration of inhibition (ie, longer dwell time in the “off” state of the CP). In turn, this means that the dose-effect relationship is demonstrated in the duration of action, such that higher doses lead to longer possible inter-dose intervals while maintaining full pathway inhibition.

2.2 BACKGROUND AND STUDY RATIONALE

The CP has been implicated in many diseases that are driven by the presence of a pathogenic antibody; CAgD is one such example. Complement inhibition has proven to be a safe and effective treatment for another form of hemolytic anemia, paroxysmal nocturnal hemoglobinuria. Currently, there are approved complement inhibitors being used therapeutically for various indications, including Soliris® (eculizumab), a mAb targeting C5; Berinert® and Cinryze®, both C1 esterase inhibitors purified from human plasma; and Ruconest®, a recombinant form of human C1 esterase inhibitor. Unlike Soliris and the C1 esterase inhibitors, by specifically targeting C1s, BIVV009 inhibits only the CP, leaving the alternative complement pathway and the lectin complement pathway available for immune surveillance. Furthermore, by blocking at the level of the C1 complex, BIVV009 is expected to prevent generation of all anaphylatoxins and opsonins (eg, C3 fragments) that produce pathologic lesions in CP-mediated disorders.

CAgD is an autoimmune hemolytic anemia caused by IgM-induced CP activation. The IgM autoantibodies in CAgD are referred to as "cold agglutinins" given their inherent property of increased binding as a function of decreased temperature. The cold agglutinin thermal amplitude, which is the highest temperature at which the cold agglutinin can be detected to react with the red blood cell antigen approaches core body temperature in many patients (1). CAgD symptoms are typically triggered by exposure to cold environmental temperatures (ie, temperatures at or below core body temperature), viral infections, or inflammation (2, 3, 4, 5, 6, 7). All patients with CAgD have active disease with varying levels of chronic, ongoing hemolysis resulting in anemia (8). CAgD patients without recent transfusion remain significantly compromised with symptoms associated with hemolytic anemia including shortness of breath, fatigue, lightheadedness, and general weakness, in addition to vascular symptoms such as Raynaud's and acrocyanosis. The symptomatic impact of anemia is increased in CAgD patients due to the advanced age of the population (1, 9) who tolerate anemia symptoms poorly. CAgD patients who have not recently been transfused often have similar Hgb levels as recently-transfused patients and can have significant symptoms (9). These complications have a significant adverse impact on quality of life. Patients with CAgD are also at increased risk of arterial and venous thromboembolic events, including potentially life-threatening events such as stroke, myocardial infarction, and pulmonary embolism (10, 11, 12). This increased risk of thromboembolic events occurs in CAgD patients with even mild anemia and is related to the ongoing hemolysis (10, 12). Thus, CAgD patients who are not recently transfused remain at risk for severe complications of the disease.

There are no currently approved therapies for CAgD. CAgD is typically not responsive to treatment with steroids or splenectomy and can only be managed by supportive measures (avoidance of cold, blood transfusions as needed), and/or immunosuppressive, cytotoxic therapies (eg, rituximab with or without fludarabine or bendamustine) (8). Because all red blood cells express the antigen targets of the IgM autoantibody (13), transfused blood in CAgD patients is subject to the same IgM-induced CP-mediated destruction as the patients' own red blood cells and is of limited efficacy beyond short-term alleviation of acute anemia symptoms. Chronic transfusion support can lead to iron overload and the need for additional chelation therapy. Immunomodulatory and cytotoxic therapies are used based on the association of CAgD with a clonal lymphoproliferative disorder found in most patients (8, 14). Targeting an underlying clonal disorder with immuno- and/or cytotoxic therapy can have significant toxicity risks with variable treatment responses that are often delayed and/or non-durable (8).

Patients with CAgD have disease activity that fluctuates over time. In population-based natural history studies on CAgD, approximately 35-49% of patients did not receive transfusion support despite being anemic (9, 15). However, approximately 21% of patients convert from transfusion independence to transfusion dependence (15) and almost all patients undergo recurrent episodes of more severe anemia exacerbations over time that require periodic transfusion support (9). Thus, the BIVV009-04 study patient population is a distinct population from CAgD patients who have been recently transfused, representing patients in a different phase of their disease with respect to transfusion usage.

CAgD patients without recent transfusion suffer from significant symptoms due both to anemia and hemolysis as described above and are at risk for thromboembolic events as well as severe

exacerbations of their disease. Patients without recent transfusion remain symptomatic and at risk of disease complications, particularly those patients with significant anemia (Hgb ≤10 g/dL).

The Phase 1b clinical trial of BIVV009 in patients with CAgD showed that it can rapidly block hemolytic activity, induce complete remission of anemia, and prevent the need for blood transfusion (16, 17). In this Phase 1b study, as well as in the completed healthy volunteer Phase 1 studies, BIVV009 was well tolerated. Based on the lack of available therapies, the eligibility criteria requiring symptomatic CAgD with anemia, the known complications including thromboembolic event risk faced by the patient population, and that most patients experience episodic worsening of their disease requiring initiation of transfusion, there is a high unmet need for this patient population. The ultimate goal in the treatment of CAgD is to control the underlying hemolysis, thus alleviating anemia and addressing the symptomatology that can have a significant impact on quality of life, as well as other complications such as hemolytic breakthrough and thromboembolic events. The absence of approved therapy and the clinical data available from prior study of BIVV009 in CAgD supports the benefit:risk in this population.

Patients with CAgD are often elderly and/or have numerous co-morbidities affecting their mobility. Moreover, clinical centers specialized in the management of CAgD are infrequent and may be located far from patients' home. Consequently, some patients may find the option of home infusions with drug against CAgD beneficial. To this end a group of patients at preselected sites/countries will be offered the possibility of home infusions during Part B, assisted by trained health care professional. For the US, the Netherlands, Norway, France, Italy, Austria, Germany, and Spain specific amendments (Appendix K).

In addition, in Part B, a subset of at least 15 patients will be administered the study drug undiluted with saline solution. An undiluted infusion gives the opportunity to administer study drug without diluting with normal saline. The volume of injection can be reduced from 500 mL to 150 mL or 130 mL, depending on body weight. Consequently, administration of undiluted study drug may be found beneficial by patients and pharmacy staff.

2.2.1 Non-clinical experience

The non-clinical safety foundation for this study includes a completed 6-month toxicology study in which NHPs were treated with weekly doses of BIVV009 as high as 180 mg/kg for 26 weeks. Non-clinical safety studies of BIVV009 have demonstrated no toxicologically adverse findings related to BIVV009 in cynomolgus monkey studies of up to 6-months treatment duration; furthermore, no adverse effects from exaggerated pharmacology (ie, autoimmune diseases, bacterial infections) were observed in those studies. As a result, the no observed adverse effect (dose) level (NOAEL) established for BIVV009 is 180 mg/kg by weekly intravenous (IV) administration for 26 weeks; this translates to a safety margin that more than adequately covers the BIVV009 dose regimen for use in Study BIVV009 -04. Hence the NOAEL in cynomolgus monkeys is more than twice the intended fixed doses of 6.5 grams or 7.5 grams, based on adults weighing approximately 75 kg.

2.2.2 Clinical experience

The clinical safety foundation for this study includes an ongoing prospective, double-blind, randomized, placebo-controlled, First In Human (FIH) Phase 1a/b study (BIVV009-01). Phase 1a included Part A, a single-ascending dose study in normal healthy volunteers (NHV), and Part B, a multiple-ascending dose study in NHVs. Phase 1b included Part C, a multi-dose study in patients with complement-mediated disorders including CAgD, WAIHA, BP, and antibody-mediated rejection (AMR) in kidney transplant recipients, and Part E, which was added to both allow continued access to study drug in a subset of study patients with CAgD, and to further characterize the safety and efficacy to BIVV009. Part D was not implemented.

Part A was conducted according to an ascending dose cohort paradigm in which a unique cohort of NHVs was treated at each single dose level. There were 7 cohorts of NHVs. The first 2 cohorts consisted of 4 healthy volunteers each, 3 given BIVV009 (0.3 or 1 mg/kg) by IV infusion and 1 given placebo. The remaining 5 cohorts consisted of 8 healthy volunteers each, 6 given BIVV009 by IV infusion (3, 10, 30, 60, or 100 mg/kg) and 2 given placebo.

Part B was conducted according to an ascending dose cohort paradigm in which a unique cohort of NHVs was treated at each dose level. There were 2 cohorts of NHVs, each consisting of 8 healthy volunteers, in this part of the study. These cohorts were given 4 weekly IV doses of BIVV009 or placebo (6:2 for active:placebo) at a dose level previously administered to NHVs in Part A of the study (30 or 60 mg/kg).

Part C was conducted in a single cohort of patients enrolled in 4 strata, representing four complement-mediated disorders (CAgD, WAIHA, BP, and AMR). Patients in Part C received a single IV test dose of 10 mg/kg followed by 4 weekly doses of 60 mg/kg.

Clinical proof of concept for BIVV009 was achieved in Phase 1b based upon the demonstration of immediate cessation of hemolysis and rapid correction of anemia during short-term treatment of patients with CAgD (a complement-mediated hemolytic anemia). These results confirm that continuous C1s inhibition is sufficient to observe a treatment effect, while the observation of relapse of hemolytic anemia upon washout of BIVV009 and restoration of C1s activity confirms that continuous C1s inhibition is necessary for treatment of CAgD.

The clinical safety profile of single- or multiple-dose administration of BIVV009 to healthy volunteers was similar to placebo with respect to type, frequency, or severity of adverse events (AEs) and there have been no clinically meaningful AEs seen in healthy volunteers exposed to BIVV009 at doses up to 60 mg/kg weekly for 4 doses. The clinical safety profile of multiple-dose administration of BIVV009 to patients with a variety of complement-mediated disorders has not revealed any new safety concerns in this older-aged, medically complex population. With respect to the hypothetical, mechanism-related risks from C1s inhibition (ie, autoimmune diseases, bacterial infections), prophylactic vaccination against encapsulated bacterial pathogens per regional guidelines and routine surveillance with systemic lupus erythematosus (SLE) serologic testing are available to mitigate these risks.

A second prospective, double-blind, randomized, placebo-controlled study of multi-dose BIVV009 in healthy volunteers was recently completed under Protocol TNT009-02. A single

cohort of 24 NHVs was randomized to BIVV009 (75 mg/kg) or placebo at a ratio of 3:1. Volunteers were dosed on Days 1, 8, 22, and 36. Intensive PK/PD/exploratory complement sampling was performed during the study. This study added an additional dose level, which was combined with data from the BIVV009-01 study to augment PK modeling and simulations. The results of this study, in conjunction with existing PK data, were used to propose dose regimens for the current protocol.

2.2.3 Pharmacokinetic experience and dose justification

The pharmacokinetic/pharmacodynamic (PK/PD) profile of BIVV009 in healthy volunteers and in patients has been established based on the data collected from the first-in-human clinical trial. A human PK model was constructed from the complete and final Phase 1a data. This model was then augmented with the available PK data emerging from the Phase 1b component of this trial, and the combined model was used to simulate a variety of possible dose regimens for use in patients. The dose regimen proposed for use in current and future clinical trials with BIVV009, including Study BIVV009-04, differs from that used in the Phase 1a and 1b program because weekly IV administration was deemed to be logistically challenging for patients when the period of treatment is increased from weeks to months. A regimen based upon a single priming dose on Day 0, followed by bi-weekly dosing on Days 7, 21, 35, 49, etc, can provide continuous, complete C1s inhibition while better accommodating to the needs of patients. Human PK modeling suggests that a dose level of 6.5 grams or 7.5 grams (based on body weight of <75 kg or ≥75 kg, respectively) is necessary to protect patients better from potential restoration of CP activity at the end of the inter-dose interval. The weight cut-off of 75 kg was chosen based on the expected weight distribution in CAgD patients with a median weight of 74.8 kg.

The in vitro and in vivo pharmacologic profile of BIVV009 has two especially important features that help to define the optimal dose regimen for clinical use. First, BIVV009 has very high affinity and selectivity for C1s, with negligible off-target activity. Second, it is a potent CP inhibitor with such a steep concentration-effect relationship that it behaves as a switch-like inhibitor of C1s: at concentrations of BIVV009 ≥20 µg/mL, CP activity is virtually undetectable; at BIVV009 concentrations below this threshold the CP is fully active. This property results in the requirement to maintain a blood concentration of BIVV009 that is always at least ≥20 µg/mL, even at trough, lest CP activity be fully restored.

2.2.4 Potential risks and benefits

As previously noted, clinical proof of concept for BIVV009 was achieved in a Phase 1b study, which demonstrated immediate cessation of hemolysis and rapid correction of anemia during short-term treatment of patients with CAgD.

The human safety risk from off-target effects of mAb therapeutics is generally considered to be low, and in this regard BIVV009 is no exception. The human safety risk from short-term inhibition of the complement system also appears to be low, based upon the experience with five approved products in this therapeutic class. Long-term, complement inhibition may increase the risk of infection with encapsulated bacteria, as reflected in the product label for eculizumab (Soliris), an inhibitor of the terminal portion of the complement system. However, to provide

optimal protection against infections with encapsulated bacteria, the design of this study includes an appropriate program of prophylactic vaccinations.

The risks associated with long-term inhibition of the proximal portion of the CP are presently unknown. Theoretically, it could increase the risk of SLE or circulating immune complexes (CIC) disease due to the role of the C1 complex in immune complex clearance, as observed in patients with congenital deficiencies of C1 complex components (C1q, C1s, and C1r). However, pharmacologic inhibition of C1s differs from congenital deficiency of the C1 complex because: 1) congenital C1 complex component deficiency are commonly not single gene mutations but typically are associated with second mutations in other immune system genes; 2) pharmacologic inhibition of C1s enzymatic function in the C1 complex leaves intact the non-enzymatic function of C1q, which is important for the opsonization and phagocytic removal of apoptotic cells which protects against autoimmunity; and 3) the phenotype associated with life-long, often total absence of C1 complex structure and function is unlikely to be reproduced by pharmacologic antagonism of C1 enzymatic function in fully developed adults. Nevertheless, standard clinical biomarkers related to SLE (eg, antibodies to double-stranded DNA [dsDNA]) have been incorporated into the study design as safety surveillance measures.

Home infusions with the study drug will be proposed to a number of patients in countries pre-selected to participate in home infusion. Home infusions will be assisted by a trained healthcare professional, and will concern patients who express such wish, after having been qualified by the Investigator and no sooner than after Week 39 (Day 273) and without evidence of intolerance of the study drug as determined by the Investigator based on the criteria outlined in Appendix K. The potential risks associated with home BIVV009 administrations are the same as the risks with BIVV009 administration in a clinical setting. Only patients treated with BIVV009 for a minimum of 3 months and who do not have a history of hypersensitivity to BIVV009 are eligible for the home infusion. Additionally, patients who are participating in or have ever participated in undiluted infusions of BIVV009 are not eligible for home infusions. Rescue medications to treat hypersensitivity/allergic reactions as per the PI's guidance will be available during home infusion visits. The home infusion personnel will be trained in basic life support (cardiopulmonary resuscitation [CPR]) but will not be required to have equipment to perform advanced life support (eg, defibrillators).

At least 15 patients in Part B will be proposed to receive infusions of the study drug undiluted with saline solution. An undiluted infusion gives the opportunity to administer study drug without diluting with normal saline. Undiluted infusions may begin no sooner than after Week 39 (Day 273) in patients who provide their consent and are without evidence of intolerance to the study drug as determined by the Investigator based on the criteria outlined in Section 4.1, Part B. Patients who are participating in or have ever participated in home infusions are not eligible for undiluted infusion. Safety concerns associated with undiluted BIVV009 are not anticipated; however, an increase in infusion-related reactions may be possible due to the altered concentration of the protein/active compound. Therefore, patients treated with BIVV009 for a minimum of 3 months and who do not have a history of hypersensitivity to BIVV009 may be eligible for undiluted infusions with BIVV009. Decreased fluid load associated with BIVV009 administration may be found clinically advantageous, particularly in this population of CAgD patients who often have numerous co-morbidities which may include cardio-pulmonary conditions. Moreover, the preparation of BIVV009 infusion without the need for dilution in

normal saline is associated with reduced complexity. There is no impact anticipated on PK between undiluted solution and diluted solution.

The overall risk/benefit balance for participants in Study BIVV009-04 is favorable based on available data to date.

3 STUDY OBJECTIVES

3.1 PRIMARY OBJECTIVE (PART A)

The primary objective of Part A is to determine whether BIVV009 administration results in a ≥1.5 g/dL increase in hemoglobin (Hgb) level and avoidance of transfusion in patients with primary CAgD without a recent history of blood transfusion.

3.2 SECONDARY OBJECTIVES (PART A)

The secondary efficacy objectives of Part A are:

- To assess the effect of BIVV009 on clinical events and laboratory parameters related to hemolysis and anemia in patients with primary CAgD
- To assess the effect of BIVV009 on specific complications of CAgD (acrocyanosis, Raynaud's syndrome, hemoglobinuria, and thromboembolism)
- To assess the effect of BIVV009 on quality of life (QOL) in patients with primary CAgD.

The safety objective of Part A is:

- To evaluate the overall safety and tolerability of BIVV009 in patients with primary CAgD

The exploratory objectives of Part A are:

- To evaluate the effect of BIVV009 on certain disease-related biomarkers in patients with primary CAgD
- To evaluate the pharmacokinetics of BIVV009
- To evaluate the immunogenicity of BIVV009

3.3 PRIMARY OBJECTIVE (PART B)

The primary objective of Part B is to evaluate the long-term safety and tolerability of BIVV009 in patients with primary CAgD.

3.4 SECONDARY OBJECTIVE (PART B)

The secondary objective of Part B is

- To investigate the durability of response during long-term treatment with BIVV009 in patients with primary CAgD
- To evaluate the immunogenicity of BIVV009

3.5 EXPLORATORY OBJECTIVE (PART B)

- To describe the safety and patient satisfaction with the convenience of home infusions with BIVV009 in a subset of patients (for the US, the Netherlands, Norway, France, Italy, Austria, Germany, and Spain specific amendments (Appendix K)
- To describe the safety of undiluted infusions with BIVV009
- To evaluate the immunogenicity of BIVV009

4 INVESTIGATIONAL PLAN

4.1 STUDY DESIGN

This randomized, double-blind, placebo-controlled study is designed to evaluate the efficacy and safety of BIVV009 in symptomatic patients with the complement-mediated disorder primary CAgD who do not have a recent history of blood transfusion.

During the 6-week Screening/Observation Period, prospective patients will have a detailed medical history documented (including all available transfusion history), physical evaluations, and blood samples collected on 3 occasions approximately every 2 weeks.

Patients may receive a transfusion(s) during the Screening/Observation Period prior to the first study drug infusion if medically indicated per the Investigator's discretion. However, the baseline visit (and first infusion of study drug) must occur at least 7 days following the transfusion.

Part A

The study will enroll approximately 40 primary CAgD patients who do not have a recent history of blood transfusion (ie, ≤1 transfusion during the last year and no transfusion during the last 6 months prior to enrollment). Eligible patients should have been diagnosed with primary CAgD at least 6 months prior to enrollment and should have had no history of transfusion during this period.

Eligible patients will be randomized 1:1 to receive an IV infusion of BIVV009 or placebo over approximately 60 minutes on Day 0, Day 7, and every 14 days thereafter through Week 25 (ie, Days 21, 35, 49, 63, 77, 91, 105, 119, 133, 147, 161, and 175). Patients who miss a dose (ie, outside the dosing window or >17 days since last dose) should return to the site for an unscheduled visit 1 week prior to the next scheduled dose in order to receive an additional loading dose. Patients will have an End-of-Treatment (EOT) visit in Part A on Day 182 (Week 26).

A

Patients who meet the transfusion criteria in Table 1 during the 6-month double-blind treatment period will receive a transfusion. Patients who receive a transfusion during Part A will not be withdrawn from the study and will be eligible to participate in Part B.

Table 1 - Transfusion criteria

B

A patient will receive a transfusion during Part A or Part B if his or her Hgb level meets either of the following criteria:<br>• Hgb is <9 g/dL and the patient is symptomatic, *or*<br>• Hgb is <7 g/dL and the patient is asymptomatic

A responder analysis will be conducted following completion of the EOT visit at Week 26. The responder definition is provided in Table 2.

Table 2 - Responder definition

A

A patient will be considered a responder in Part A if:

- Hgb increases ≥1.5 g/dL from baseline (defined as the last Hgb value before administration of the first dose of study drug) at treatment assessment endpoint (defined as mean value from Weeks 23, 25, and 26) and
- The patient did not receive a blood transfusion from Week 5 through Week 26 (EOT), and
- The patient did not receive treatment for CAgD beyond what is permitted per protocol from Week 5 through Week 26 (EOT)

A list of excluded concomitant medications, as well as allowed concomitant medications with restrictions, is provided in Section 6.2.6. Beyond the permitted concomitant medications, study drug, and transfusions, patients may receive no other therapies for the treatment of CAgD while enrolled in this study; patients requiring other treatment for their CAgD in Part A will be withdrawn from the study and counted as non-responders. These patients will not be eligible to participate in Part B.

B

Part B

Following completion of dosing in the initial 6-month treatment period, patients will roll into the long-term safety and durability of response extension phase and receive BIVV009 in an open-label manner. Part B will run for 1 year following last patient out (LPO) under Part A. Patients requiring treatment with permitted concomitant medications and/or transfusions will not be discontinued from the study. Patients in Part B will be transfused per the transfusion criteria in Table 1. Patients who receive a transfusion in Part B will not be withdrawn from the study.

Blinding will be maintained when rolling patients into the extension period by providing a crossover dose at Week 26 to allow placebo patients to receive the BIVV009 loading dose at start of BIVV009 dosing. Patients who were randomized to BIVV009 during the 6-month treatment period will receive a placebo dose at Week 26 to maintain blinding. All patients will then continue to receive bi-weekly BIVV009 dosing starting at Week 27.

Patients will be dosed with BIVV009 every 2 weeks, as in Part A. Should patients deviate from their scheduled dosing, a repeat loading dose may be required. On-site visits will be completed ~ every 3 months (at a minimum) for collection of PK, PD and ADA samples, and additional safety and efficacy measures.

Home infusion will be performed by a healthcare professional caregiver contingent upon completion of training delivered by the Investigator or delegated site staff member only in patients willing to be administered study drug in home, who have been treated with at least 3 months with BIVV009 in Part B and who satisfy the criteria mention in the appendix K (Appendix K).

A subset of patients from countries pre-selected to participate in home infusion, who have been treated for a minimum 3 months (completed visit Day 273) in Part B and who were determined

to have tolerated BIVV009 well, will be invited to have infusions with BIVV009 performed at their homes, after having been qualified by the Investigator. Home infusion will be performed by a healthcare professional caregiver contingent upon completion of training delivered by the Investigator or delegated site staff member. Patients will follow the alternate home infusion scheme, ie, home infusion at the patient's home will be alternating with office visits, so that patients will attend office visit every 4 weeks alternating with home infusions every 4 weeks (±2 days) (Appendix K).

At least 15 patients in Part B will be proposed to receive infusions with study drug undiluted with saline solution. Undiluted infusions may begin in patients who consent to and without evidence of intolerance of the study drug and who satisfy the following criteria:

1. Able to comprehend and give informed consent for receiving undiluted infusions of BIVV009 and willing to be infused for at least 1 dose
2. Treated for at least 3 months in Part B (completed visit Day 273)
3. No history of hypersensitivity reaction to BIVV009
4. Not selected to participate in home infusion administration
5. Considered by Investigator as suitable for undiluted infusion of BIVV009

Each qualified patient is supposed to receive at least one undiluted infusion of BIVV009, however, patients are free to continue with undiluted infusion of BIVV009 until the end of study. Undiluted infusions with BIVV009 versus infusions of BIVV009 diluted in saline will be captured through the case report form (CRF) for drug administration.

A safety follow-up visit for collection of AE data, PK, PD, and anti-drug antibody (ADA) samples will be performed 9 weeks after administration of the last dose of study drug in patients who discontinue early or following the end of dosing in the long-term extension period. Samples for PK, PD, and ADA will also be collected from patients who experience a hematological breakthrough event. The study will be complete 12 months following LPO for Part A at which time all patients receiving on-going treatment will proceed to an End-of-Study (EOS) visit.

4.2 DISCUSSION OF STUDY DESIGN

The route of administration, dose, and dosing interval for BIVV009 planned for use in this study are based on the initial Phase 1a/1b clinical experience (BIVV009-01) and corresponding non-clinical data for safety, PK, and PD observations. BIVV009 has thus far been administered to healthy human volunteers and patients with complement-mediated disease entities including CAgD, BP, WAIHA, and AMR.

During Part A, randomized patients will receive fixed doses via IV infusion of either 6.5 grams (if <75 kg) or 7.5 grams (if ≥75 kg) of BIVV009 or placebo based on their baseline body weight. During Part B, all patients will receive BIVV009 in an open-label manner based on their body weight as described above.

The repeated dose regimen is predicted to provide continuous C1s inhibition throughout the dosing interval, with an adequate safety margin based on comparative drug exposures in NHPs

(Section 2.2.1) and in healthy volunteers given a single dose of 100 mg/kg in Phase 1a (Section 2.2.2).

Safety, tolerability, PK, PD and immunogenicity assessments will be evaluated at the time points indicated in the study schedule of events (Table 3). In patients having consented to the use of their blood samples for future research, ADA may be tested using available predose PD back-up samples.

4.3 STUDY ENDPOINTS

4.3.1 Primary endpoint (Part A)

The primary efficacy endpoint is the responder rate as defined in Table 2.

4.3.2 Secondary efficacy endpoints (Part A)

- Mean change from baseline in Hgb at treatment assessment endpoint (mean of values at Week 23, 25, and 26)
- Mean change from baseline in bilirubin (excluding patients with Gilbert's Syndrome) at treatment assessment endpoint
- Mean change from baseline in QOL, as assessed by the change in Functional Assessment of Chronic Illness Therapy (FACIT)-Fatigue scale (Appendix C) scores at the treatment assessment endpoint
- Mean change from baseline in lactate dehydrogenase (LDH) at the treatment assessment endpoint
- Incidence of solicited symptomatic anemia at EOT

A

4.3.3 Exploratory efficacy endpoints (Part A)

- Mean change from baseline in QOL, as assessed by the change in the five level EuroQol – five dimensions questionnaire (EQ-5D-5L) scores at the treatment assessment endpoint
- Mean change from baseline in QOL, as assessed by the change in the 12-Item Short Form Survey (SF-12®) at the end of treatment assessment endpoint
- Proportion of patients with ≥12 g/dL Hgb at treatment assessment endpoint
- Incidence of thromboembolic events after the first 5 weeks of study drug administration
- Median time to normalization of bilirubin
- Median time to normalization of LDH
- Median time to normalization of haptoglobin
- Median time to Hgb of ≥12 g/dL
- Proportion of patients normalizing haptoglobin at treatment assessment endpoint

B

- Proportion of patients normalizing bilirubin at treatment assessment endpoint
- Proportion of patients with abnormal LDH at baseline who normalize LDH at treatment assessment endpoint
- Patient's Global Impression of Change (PGIC) to assess the patient's perception of changes in CAgD disease burden at EOT
- Patient's Global Impression of [Fatigue] Severity (PGIS) to assess the patient's perception of changes in fatigue at EOT
- Incidence of disabling circulatory symptoms at treatment assessment endpoint
- Total healthcare resource utilization at EOT

4.3.4 Efficacy endpoints (Part B)

The following parameters of disease activity will be assessed:

- Hemoglobin
- Bilirubin (total)
- QOL assessments (FACIT-fatigue, EQ-5D-5L, SF-12, PGIS, and PGIC)
- LDH
- Transfusion requirements
- Haptoglobin
- Total healthcare resource utilization at EOT
- Satisfaction with home infusion after first home infusion and after fourth home infusion will be assessed in patients with home infusions (Appendix K, Appendix L)

4.3.5 Safety endpoints

A

- Incidence of treatment-emergent AEs (TEAEs) and serious AEs (SAEs)
- Change from baseline in clinical laboratory evaluations
- Change from baseline in SLE panel
- Change from baseline in vital signs
- Change from baseline in electrocardiogram (ECG) data
- Physical examination findings
- Serum disease-related biomarkers
- Incidence of hemolytic breakthrough (rapid fall in Hgb ≥2 g/dL associated with an increase in LDH/bilirubin and/or decrease in haptoglobin since the last scheduled visit) through the EOT at Week 26
- Incidence of infections of ≥ Grade 3 severity (ie, requiring IV antibiotics)

- Incidence of thromboembolic events
- For patients with home infusions, safety assessments will additionally include AEs with onset within 24 hours of the of infusion
- For patients receiving undiluted infusions, safety assessments will additionally include post-infusion vital signs and AEs with onset within 24 hours of the infusion

4.3.6 Pharmacokinetic endpoints

- Plasma concentrations of BIVV009
- PK parameters. Appropriate exposure parameters (C_{max}, AUC) will be derived using a population PK approach.

During Part A, PK blood samples will be collected at predose and 1 hour postdose (ie, 1 hour after completion of study drug infusion) from all patients on Days 0, 7, 21, 35, 49, 63, 77, 91, 105, 119, 133, 147, 161, and 175. An additional blood sample for PK analysis will be collected during the EOT visit on Day 182 or at early termination (ET) if a patient withdraws early.

During Part B, PK samples will continue to be collected at predose and 1 hour (±15 minutes) postdose on Days 189, 217, and 245, then routinely at 3-month intervals starting at Day 273 through the remainder of the study. Samples will also be collected if a patient experiences a hematologic breakthrough event or withdraws from the study.

A

4.3.7 Pharmacodynamic endpoints

PD Primary Outcome Measure:

- Wieslab-CP

Exploratory Complement System Measures:

- CH50
- Total C4
- C1q
- C1s

During Part A, PD blood samples will be collected at predose and 1 hour postdose (ie, 1 hour after completion of study drug infusion) from all patients on Days 0, 7, 21, 35, 49, 63, 77, 91, 105, 119, 133, 147, 161, and 175. An additional blood sample for PD analysis will be collected during the EOT visit on Day 182 or at ET if a patient withdraws early.

During Part B, PD samples will continue to be collected at predose and 1 hour postdose (±15 minutes) on Days 189, 217, and 245, then routinely at 3-month intervals starting at Day 273 through the remainder of the study. Samples will also be collected if a patient experiences a hematologic breakthrough event or withdraws from the study.

4.3.8 Immunogenicity endpoints

During Part A, ADA samples will be collected at predose on Day 0, 7, 35, 77, 133, 175 and on Day 182 (EOT) or at ET if a patient withdraws early. During Part B, ADA samples will continue to be collected at predose, on Days 189, 217, and 245, then routinely at 3-month intervals starting at Day 273 through the remainder of the study and at Safety Follow up visit 9 weeks after last dose. Samples will also be collected if a patient experiences a hematologic breakthrough or withdraws from the study.

In patients enrolled prior protocol v6.0 and having consented to the use of their blood samples for future research, ADA may be tested using available predose PD back-up samples.

4.4 DURATION OF THE STUDY

The planned total study duration per patient is approximately 1.5 to 2.5 years:

- Screening/Observation Period: 6 weeks (Day -42 through Day -1)
- Part A treatment period: 25 weeks (Day 0 through Day 175)
- Part A EOT visit: 1 week after administration of the last dose of study drug during Part A (Week 26/Day 182)

Patients who complete Part A per protocol through the EOT visit will participate in Part B, the long-term safety and durability of response extension phase of the study.

- Part B safety and durability of response extension phase: blinding will be maintained when rolling patients into the extension period by providing a crossover dose at Week 26 to allow placebo patients to receive the BIVV009 loading dose at start of BIVV009 dosing. Patients who were randomized to BIVV009 during the 6-month treatment period will receive a placebo dose at Week 26 to maintain blinding. All patients will then continue to receive bi-weekly BIVV009 dosing starting at Week 27. For individual patients, dosing in Part B may last from 1 to 2 years, depending on when the patient enters Part B. Part B will to run for 1 year following completion of LPO in Part A.
- Part A/B ET/Safety Follow-up visit: 9 weeks after administration of the last dose of study drug.

4.5 END OF STUDY

The study will be considered complete 12 months following LPO from Part A. When this occurs, all ongoing patients in Part B will return to the clinic for EOS assessments (see Section 6.1.5.7 and Table 3). The EOS will occur when the last patient has had his or her last visit (Last Patient Last Visit).

5 PATIENT SELECTION

This study will enroll approximately 40 adult patients with primary CAgD who do not have a recent history of blood transfusion. Patients who meet all the inclusion criteria and for whom none of the exclusion criteria apply will be eligible for enrollment.

5.1 INCLUSION CRITERIA

All patients must meet all the following inclusion criteria to be enrolled:

A

I 01. Adult male and female patients ≥18 years of age at Screening.

I 02. Body weight of ≥39 kg at Screening.

I 03. Confirmed diagnosis of primary CAgD based on the following criteria:

a) Chronic hemolysis
b) Polyspecific direct antiglobulin test (DAT) positive
c) Monospecific DAT strongly positive for C3d
d) Cold agglutinin titer ≥64 at 4˚C
e) IgG DAT ≤1+, and
f) No overt malignant disease

I 04. Hemoglobin level ≤10.0 g/dL.

I 05. Bilirubin level above the normal reference range, including patients with Gilbert's Syndrome.

I 06. Ferritin levels above the lower limit of normal. Concurrent treatment with iron supplementation is permitted if the patient has been on a stable dose during the previous 4 weeks.

B

I 07. Presence of one or more of the following CAgD-related signs or symptoms within 3 months of Screening:

a) Symptomatic anemia defined as:
   i. Fatigue
   ii. Weakness
   iii. Shortness of breath
   iv. Palpitations, fast heart beat
   v. Light headedness and/or
   vi. Chest pain
b) Acrocyanosis
c) Raynaud's syndrome

A

d) Hemoglobinuria

e) Disabling circulatory symptoms, and/or

f) Major adverse vascular event (including thrombosis)

I 08. Bone marrow biopsy within 6 months of Screening with no overt evidence of lymphoproliferative disease or other hematological malignancy. An additional bone marrow biopsy will be required if the prior bone marrow is deemed unsuitable for analysis by the Sponsor.

B

I 09. Documented vaccinations against encapsulated bacterial pathogens (*Neisseria meningitis*, including serogroup B *meningococcus*, *Haemophilus influenzae*, where available, and *Streptococcus pneumoniae*) within 5 years of enrollment or as specified in Section 6.1.1.1.

I 10. Patients must be willing to receive transfusions if they meet the eligibility criteria during the study treatment period. Patients who do not have a recent history of transfusion due to patient refusal or patient decision should not be enrolled if they do not agree to receive blood transfusions as needed.

I 11. Adequate IV access.

I 12. If female, must be post-menopausal, surgically sterile, or be established on (≥3 months prior to Screening) and agree to continue to use the same highly effective methods of birth control throughout the study and for 9 weeks following administration of the last dose of study drug.

I 13. Males must be surgically sterile for at least 90 days or when sexually-active with female partners of child-bearing potential will agree to use highly effective contraception from Day 0 until 9 weeks following administration of the last dose of study drug.

I 14. Able to comprehend and give informed consent.

I 15. Able to comply with the requirements of the study and to complete the full sequence of protocol-related procedures.

5.2 EXCLUSION CRITERIA

Patients who meet any of the following criteria will be excluded from the study:

C

E 01. Cold agglutinin syndrome secondary to infection, rheumatologic disease, or active hematologic malignancy.

E 02. History of blood transfusion within 6 months of screening, or history of more than one blood transfusion within 12 months of screening.

E 03. Clinically relevant infection of any kind within the month preceding enrollment (eg, active hepatitis C, pneumonia).

A

E 04. Clinical diagnosis of SLE; or other autoimmune disorders with anti-nuclear antibodies at Screening. Anti-nuclear antibodies of long-standing duration without associated clinical symptoms will be adjudicated on a case-by-case basis during the Confirmatory Review of Patient Eligibility (Section 6.1.1.3).

E 05. Positive hepatitis panel (including hepatitis B surface antigen and/or hepatitis C virus antibody) prior to or at Screening.

E 06. Positive human immunodeficiency virus (HIV) antibody at Screening.

C

E 07. Treatment with rituximab monotherapy within 3 months or rituximab combination therapies (eg, with bendamustine, fludarabine, ibrutinib, or cytotoxic drugs) within 6 months prior to enrollment.

E 08. Concurrent treatment with corticosteroids other than a stable daily dose equivalent to ≤10 mg/day prednisone for previous 3 months.

B

E 09. Erythropoietin deficiency. Concurrent treatment with erythropoietin is permitted if the patient has been on a stable dose for the previous 3 months.

E 10. Concurrent usage of iron supplementation unless the patient has been on a stable dose for at least 4 weeks.

E 11. Clinically significant medical history or ongoing chronic illness that would jeopardize the safety of the patient or compromise the quality of the data derived from his/her participation in this study (as determined by the Investigator [or designee]) at Screening.

E 12. Concurrent treatment with other experimental drugs or participation in another clinical trial with any investigational drug within 30 days or 5 half-lives, whichever is greater, prior to treatment start.

E 13. Females who are pregnant, lactating, or, if having reproductive potential, are considered potentially unreliable with respect to contraceptive practice.

E 14. History of hypersensitivity to BIVV009 or any of its components.

For home infusion in/exclusion criteria see (Appendix K).

For undiluted infusion eligibility criteria see Section 4.1.

5.3 REMOVAL OF PATIENTS FROM STUDY PARTICIPATION, AND STUDY SUSPENSION AND STOPPING RULES

Patients will be informed that they are free to withdraw from the study at any time and for any reason. Patients should inform the site of withdrawal in writing. The Investigator (or designee) may remove a patient from the study if, in the Investigator's (or designee's) opinion, it is not in the best interest of the patient to continue the study. Patients may be withdrawn due to the following:

- Change in compliance with inclusion/exclusion criteria that is clinically relevant and affects patient safety
- Occurrence of AEs that, in the opinion of the Investigator, may jeopardize patient safety or data integrity. This includes clinically significant hematologic breakthrough events attributable to the development of ADA and/or the development of positive SLE auto-antibody titers
- Occurrence of pregnancy in patient while receiving study drug
- Intake of non-permitted concomitant medication that might affect patient safety or study assessments/objectives
- Clinical signs of SLE or any other immune complex disease
- Hypersensitivity or allergic reaction, including anaphylaxis, to study drug

The Investigator will immediately notify the Sponsor's Study Monitor of all patients who withdraw from treatment. In case of withdrawal, all ET assessments should be performed as applicable (Section 6.1.4). The date the patient is withdrawn from the study and the reason for withdrawal will be recorded on the patient's electronic Case Report Form (eCRF). All withdrawn patients will be followed until resolution of all their AEs or until the unresolved AEs are judged by the Investigator (or designee) to have stabilized. Patients who withdraw from study early (prior to Week 5) may be replaced at the discretion of the Sponsor. Patients who withdraw from the after Week 5 and prior to Week 23 will not be eligible to participate in Part B.

The entire study may be discontinued at the discretion of the Sponsor based on the occurrence of the following:

- Adverse events unknown to date or increased frequency, and/or severity, and/or duration of known AEs,
- Results of the interim analysis (Section 7.9) demonstrating absence of clinically significant increases in Hgb,
- Medical or ethical reasons affecting the continued performance of the study,
- Difficulties in the recruitment of patients,
- Cancellation of or change in drug development program per the discretion of the Sponsor.

6 STUDY PROCEDURES

6.1 SCHEDULE OF STUDY PROCEDURES

A schedule of events is presented in Table 3. Laboratory tests, including PD assays, are specified in Appendix A.

Table 3 - Study schedule of events

Study Visit (Week/Day)	Screening/ Observation Period[a]	Baseline	Part A Dosing (Weeks 1–25)	Part A EOT: Part B Crossover Loading Dose (Week 26)	Part B Open-Label Extension Phase	ET/EOS/ Safety Follow-up[b]
	Days -42 to -1	Day 0	Days 7, 21, 35, 49, 63, 77, 91, 105, 119, 133, 147, 161, 175	Day 182	Every 2 Weeks after Week 25	9 Weeks after Last Dose
**Visit Windows**	± 3 days	N/A	± 2 days	± 2 days	± 2 days	± 2 days
Written informed consent	X					
Demographic & baseline characteristics	X					
Detailed medical history	X					
Inclusion/exclusion criteria	X	X				
Immunization review/vaccination[c]	When applicable, vaccinations should be initiated on Day -42 or as soon as possible during Screening. The primary vaccine series should be completed during Screening when possible and otherwise prior to Week 5 of Part A. See Appendix I for the vaccination schedule for Japan.					
Bone marrow biopsy report review[d]	X					
Optional bone marrow testing for MYD88 status for consenting patients	X					
Pregnancy test (if applicable)[e]	X	X	X (Prior to study drug infusion on Days 21, 49, 77, 105, 133, and 161)[e]	X	X[e]	X
Body weight and height[f]	X	X		X	X[l]	X
Physical examination, full	X			X		X
Physical examination, brief		X	X		X[l]	

Study Visit (Week/Day)	Screening/ Observation Period[a]	Baseline	Part A Dosing (Weeks 1–25)	Part A EOT: Part B Crossover Loading Dose (Week 26)	Part B Open-Label Extension Phase	ET/EOS/ Safety Follow-up[b]
	Days -42 to -1	Day 0	Days 7, 21, 35, 49, 63, 77, 91, 105, 119, 133, 147, 161, 175	Day 182	Every 2 Weeks after Week 25	9 Weeks after Last Dose
**Visit Windows**	± 3 days	N/A	± 2 days	± 2 days	± 2 days	± 2 days
Vital signs (BP, PR, RR, body temperature)[g]	X	X	X	X	X[l]	X
12-lead electrocardiogram (predose and 1 hour after infusion on dosing days)	X	X	X (Day 91 only)		X[p]	
Virology/serology panel[h]	X					
Gilbert's Syndrome test (UGT1A1 gene)	X					
SLE panel[h]	X			X	X[t]	X
Iron panel and erythropoietin[h]	X					
Hematology panel[h]	X	X	X	X	X	X
Coagulation panel[h]	X	X		X		X
Clinical chemistry panel[h]	X	X	X	X	X	X
Urinalysis[h]	X	X		X	X[l]	X
FACIT-Fatigue[r]		X	X	X	X[l]	X
PGIS[r]		X	X (Days 35, 77, and 119 only)	X	X[l]	X
PGIC[r]		X	X (Days 35, 77, and 119 only)	X	X[l]	X
SF-12[r]		X	X (Days 35, 77, and 119 only)	X	X[l]	X
EQ-5D-5L[r]		X	X (Days 49, 91, 133 only)	X	X[l]	X

Study Visit (Week/Day)	Screening/ Observation Period[a]	Baseline	Part A Dosing (Weeks 1–25)	Part A EOT: Part B Crossover Loading Dose (Week 26)	Part B Open-Label Extension Phase	ET/EOS/ Safety Follow-up[b]
	Days -42 to -1	Day 0	Days 7, 21, 35, 49, 63, 77, 91, 105, 119, 133, 147, 161, 175	Day 182	Every 2 Weeks after Week 25	9 Weeks after Last Dose
**Visit Windows**	**± 3 days**	**N/A**	**± 2 days**	**± 2 days**	**± 2 days**	**± 2 days**
Solicited symptomatic anemia	X (Day -42 only)	X	X	X	X	X
Study drug administration[i]		X	X	X[m]	X	
ADAs against BIVV009		X	X[s]	X[s]	X[s]	X
PK Samples[j]		X	X	X	X[l]	X
PD Samples[h, j]	X[n]	X	X	X	X[l]	X
Disease-related biomarkers[h]	X	X	X[o] (Day 91 only)	X		
Prior & concomitant medications including transfusions	X	X	X	X	X	X
Healthcare resource utilization	X	X	X (Days 21, 49, 77, 105, 133, and 161)	X	X[q]	X
Adverse events[k]	X	X	X	X	X	X

ADA = anti-drug antibodies; BP = blood pressure; EOS = End of Study; EOT = End of Treatment; EQ-5D-5L = five level EuroQol five dimensions questionnaire; ET = Early Termination Visit; FACIT-Fatigue = functional assessment of chronic illness therapy - fatigue; N/A = not applicable; PD = pharmacodynamic; PGIC = Patient's Global Impression of Change; PGIS = Patient's Global Impression of [Fatigue] Severity; PK = pharmacokinetic; PR = pulse rate; QOL = quality of life; RR = respiratory rate; SF-12 = 12-Item Short Form Survey; SLE = systemic lupus erythematosus.

a The 6-week Screening/Observation Period may be extended by 1 week for patients requiring a blood transfusion during Screening/Observation prior to study drug administration.

b Patients should return to site 9 weeks after last dose for ET procedures, EOS assessment, or Safety Follow-up procedures upon completion of dosing in the study. If patient experiences a hematological breakthrough event, a PK, PD, and ADA sample should be collected at the time of the event.

c Applicable to patients who do not have documented vaccination against encapsulated bacterial pathogens within 5 years of enrollment. Refer to immunization recommendations per Section 6.1.1.1 for patients requiring vaccination. A blood sample for vaccine titers will be collected on Day 0 prior to study drug infusion to be used to determine serum relevant antibody titers should the patient be diagnosed with an infection associated with an encapsulated organism during the study. A second sample will be collected during the study period if a patient presents with symptoms of infection.

d Prior bone marrow biopsy report review, prior tissue assessment, or new bone marrow biopsy (as applicable). Sites will submit de-identified biopsy reports to independent central reader for eligibility adjudication. If biopsy is deemed unsuitable or insufficient to determine eligibility, either prior tissue may be submitted for additional hematopathology assessment or a new bone marrow biopsy will be performed during the screening period.

e Females of child-bearing potential only. Serum pregnancy test to be performed at Screening. Serum or urine pregnancy test in WOCBP (ie, women of child bearing potential) to be performed on Days 0, 21, 49, 77, 105, 133, and 161, and at Week 26/EOT or at the ET visit. Repeat serum or urine pregnancy test every 4 weeks (±2 days) during Part B.

f Height measured at Screening only. Body weight measured every 3 months during Part B.

g Vital signs measurements (supine BP, PR, RR, and oral temperature) are to be obtained at Screening and at each subsequent visit, with measurements performed predose and 1 hour (±5 minutes) after completion of administration of each dose of study drug.

h For a complete list of analytes, see protocol Appendix A.

i Study drug doses of 6.5 grams (if <75 kg) or 7.5 grams (if ≥75 kg) based on patient's baseline body weight will be administered via IV infusion over ~ 60±5 minutes on Days 0, 7, and every 14 days thereafter during Part A and every 2 weeks starting at Week 27 during Part B. Patients with underlying cardiopulmonary disease may receive a 2-hour infusion with Sponsor approval. If a patient misses a scheduled dose (outside of the 2-day window or >17 days since last dose), they must return to site (unscheduled visit) to receive another loading dose 1 week prior to the next scheduled dose. Qualifying patients at participating sites may have study drug dosed at home during certain visits in Part B, according to the rules specified in Appendix K (for the US, the Netherlands, Norway, France, Italy, Austria, Germany, and Spain specific amendments (Appendix K).

j During Part A, PK and PD blood samples will be collected at predose and 1 hour (±15 minutes) post-dose (ie, 1 hour after completion of study drug infusion) from all patients on Days 0, 7, 21, 35, 49, 63, 77, 91, 105, 119, 133, 147, 161, and 175. An additional blood sample for PK and PD analysis will be collected during the EOT visit on Day 182 or at ET if a patient withdraws early. During Part B, PK and PD samples will continue to be collected at predose and 1 hour (±15 minutes) postdose on Days 189, 217, and 245, then routinely at 3-month intervals starting at Day 273 through the remainder of the study. PK and PD samples will also be collected if a patient experiences a hematologic breakthrough event at any point during the study.

k AEs will be recorded from the time the patient signs the informed consent form until 9 weeks after administration of the last dose of study drug.

l To be performed every 3 months during the on-site visits, or for post-infusion vital signs in case of undiluted administration, after each undiluted infusion.

m After completion of all Part A EOT assessments, a cross-over loading dose will be administered to patients in a blinded manner at the Week 26 visit to initiate dosing in Part B.

n Refer to the Laboratory Manual for details of sample collection during Screening.

o Samples will be collected for a subset of the disease-related biomarkers at Day 91. Refer to the Laboratory Manual for details.

p During Part B, a 12-lead ECG will be conducting pre- and postdose at 3 months (Day 273).

q During Part B, the healthcare resource utilization data will be recorded every 4 weeks.

r To be performed in the following order: FACIT-Fatigue first, PGIS second, PGIC third, SF-12 fourth, and EQ-5D-5L fifth.

s During Part A, ADA samples will be collected at predose on Day 0, Day 7, 35, 77, 133, 175 and on Day 182 (EOT) or at ET if a patient withdraws early. During Part B, ADA samples will continue to be collected at predose, on Days 189, 217, and 245, then routinely at 3-month intervals starting at Day 273 through the remainder of the study and at safety follow up visit 9 weeks after last dose. Samples will also be collected if a patient experiences a hematologic breakthrough event or withdraws from the study early.

t SLE panel will be performed every 6 months in Part-B of the study.

6.1.1 Screening/observation period (Days -42 to Day -1)

Patients (or their legally authorized representative) must provide informed consent before any study-specific screening tests are performed. Participating study sites are required to document all screened candidates initially considered for inclusion in the study. Patients will be designated as screened following completion of all screening assessments.

Screen failures are defined as patients who sign the informed consent form (ICF) but are not subsequently dosed with BIVV009 or placebo. If a patient is considered a screen failure, the reason(s) will be documented on the screening log and in source records.

A minimal set of screen failure information is required to ensure transparent reporting of screen failure participants to meet the Consolidated Standards of Reporting Trials publishing requirements and to respond to queries from regulatory authorities. Minimal information includes demography, screen failure details, eligibility criteria, and any SAEs.

6.1.1.1 Screening assessments (initiating at Day -42)

The following assessments and procedures will be initiated following collection of written informed consent at Day -42 and completed during the 6-week Screening/Observation Period:

- Written informed consent
- Demographic and baseline characteristics including height and body weight
- Detailed medical history
- Immunization review and vaccination, if applicable
- Serum pregnancy test (if applicable)
- Physical examination (full)
- Vital signs
- Virology/serology panel
- Gilbert’s Syndrome testing (UGT1A1 gene)
- SLE panel
- Hematology panel
- Coagulation panel
- Clinical chemistry panel
- Urinalysis
- PD samples (refer to Laboratory Manual)
- Iron panel and erythropoietin assays
- Disease-related biomarkers

- 12-lead ECG
- Prior bone marrow biopsy report review, prior tissue assessment, or new bone marrow biopsy (as applicable)
- Optional bone marrow testing for MYD88 status
- Inclusion/exclusion criteria review for determination of eligibility
- Solicited symptomatic anemia assessment
- Record healthcare resource utilization data from previous 6 months
- Record prior and concomitant medications/procedures including transfusions
- Adverse event monitoring

Bone Marrow Biopsy

A bone marrow biopsy is required to have been performed within 6 months of Screening to rule out overt evidence of lymphoproliferative disease or other hematological malignancy prior to enrollment. Sites will submit de-identified biopsy reports to an independent central reader for eligibility adjudication. If biopsy is deemed unsuitable or insufficient to determine eligibility, then either the prior tissue may be submitted for additional hematopathology assessment or a new bone marrow biopsy will be performed during the screening period. Patients will have the option to consent to additional testing of bone marrow for MYD88 status. Further instructions for bone marrow biopsy assessment will be available in Appendix A (Bone Marrow Biopsy Report Review Process) to the 'Subject Eligibility Review Process Overview'.

Vaccination Against Encapsulated Bacterial Pathogens

In the event a patient does not have documented vaccination against encapsulated bacterial pathogens (*Neisseria meningitis*, including serogroup B *meningococcus*, where available, *Haemophilus influenzae*, and *Streptococcus pneumoniae*) within 5 years prior to enrollment, vaccination should be initiated during the Screening/Observation Period prior to enrollment (see Table 3). Vaccination series for these pathogens should be completed as per current regional guidelines specified for patients with persistent complement deficiency and in accordance with their respective labels, as applicable. Where no regional guidelines are available for patients with persistent complement deficiency, it is recommended that vaccinations include meningococcal conjugate, meningococcal serogroup B, 13-valent pneumococcal, 23-valent pneumococcal, and *Haemophilus influenza* Type b vaccines where commercially available.

Vaccinations should be initiated on Day -42 or as early as possible during the Screening/Observation period. The primary vaccine series should be completed during Screening when possible and otherwise prior to Week 5 of Part A. Patients must be advised that vaccination may not prevent meningococcal infections and that they should immediately report fevers or other symptoms consistent with acute infection to the Investigator.

Patients who develop symptoms consistent with an infection due to encapsulated bacterial pathogens during the study period will have a blood sample collected to test for confirmation of infection and vaccination status.

A separate vaccination schedule for Japan is provided in Appendix I.

Laboratory Tests

The first set of laboratory test results collected during the Screening Period will be utilized to assess patient eligibility for the study. Screening assessments may be repeated once at the discretion of the Principal Investigator (PI). Additional repeat screening tests may not be performed without Sponsor (or designee) approval.

6.1.1.2 Interim visits during screening/observation period

During the 6-week Screening/Observation Period, the patient will return to the clinical site approximately every 2 weeks (Day -28 and Day -14) after the initial visit for collection of laboratory samples to assess and characterize their CAgD. During these visits, the following assessments and procedures will be performed:

- Vital signs
- Collection of blood samples for assessment of the following indicators of CAgD:
  - Hemoglobin
  - Bilirubin
  - LDH
  - Haptoglobin
  - Reticulocytes
  - CH50
- Record prior and concomitant medications/procedures including transfusions
- Adverse event monitoring

Detailed information on sample collection and a list of analytes to be tested may be found in the Study Schedule of Events (Table 3) in Section 6.1, Appendix A, and the Laboratory Manual.

6.1.1.3 Confirmatory review of patient eligibility

Upon completion of screening assessments, the site will compile de-identified, key eligibility data for each patient and forward to the Study Medical Monitor for review and confirmation of eligibility prior to study enrollment/randomization. Details on the eligibility data review process may be found in the 'Subject Eligibility Process Overview'.

6.1.2 Day 0 visit (first dose)

On Day 0, patients will undergo the following procedures before administration of the study drug:

- Inclusion/exclusion criteria review for confirmation of continued eligibility
- Serum or urine pregnancy test (if applicable)

- Vaccine titers
- Body weight
- Physical examination (brief)
- Vital signs
- Hematology panel
- Coagulation panel
- Clinical chemistry panel
- Urinalysis
- Solicited symptomatic anemia assessment
- QOL assessments
  - FACIT-Fatigue (Appendix C)
  - EQ-5D-5L (Appendix D)
  - SF-12 (Appendix E)
  - PGIS (see Appendix H)
  - PGIC (see Appendix F)
- ADAs against BIVV009
- PK and PD sampling
- Disease-related biomarkers
- Record healthcare resource utilization data (see Appendix G) since previous assessment
- 12-lead ECG
- Record prior and concomitant medications/procedures including transfusions
- Adverse event monitoring

At 0 hour, study drug will be infused via an indwelling IV catheter over a period of 60±5 minutes. Patients with underlying cardiopulmonary disease may receive a 2-hour infusion with Sponsor approval. Additionally, study drug infusion may be interrupted or slowed in the event of suspicion of an allergic reaction or anaphylaxis.

After completion of the infusion, patients will undergo the following procedures:

- Vital signs at 1 hour (±5 minutes) postdose
- 12-lead ECG at 1 hour (±15 minutes) postdose
- PK and PD sampling at 1 hour (±15 minutes) postdose

6.1.3 Weeks 1-25 Part-A

On Days 7, 21, 35, 49, 63, 77, 91, 105, 119, 133, 147, 161, and 175, patients will undergo the following procedures before administration of the study drug:

- Serum or urine pregnancy test in WOCBP (ie, women of child bearing potential), if applicable, on Days 21, 49, 77, 105, 133, and 161
- Physical examination (brief)
- Vital signs
- Hematology panel
- Clinical chemistry panel
- Solicited symptomatic anemia assessment
- QOL assessments:
  - FACIT-Fatigue: all scheduled visits
  - PGIS: Days 35, 77, and 119 only
  - PGIC: Days 35, 77, and 119 only
  - SF-12: Days 35, 77, and 119 only
  - EQ-5D-5L: Days 49, 91, and 133 only
- 12-lead ECG (Day 91 only)
- During Part A, ADA samples will be collected at predose on Day 0, 7, 35, 77, 133, 175 on Day 182 (EOT)
- PK and PD sampling
- Disease-related biomarkers (subset at Day 91 only); refer to the Laboratory Manual for details
- Record healthcare resource utilization data since previous assessment on Days 21, 49, 77, 105, 133, and 161
- Record concomitant medications/procedures including transfusions
- Adverse event monitoring

At 0 hour, study drug will be infused via an indwelling IV catheter over a period of 60 ±5 minutes. Patients with underlying cardiopulmonary disease may receive a 2-hour infusion with Sponsor approval. Additionally, study drug infusion may be interrupted or slowed in the event of suspicion of an allergic reaction or anaphylaxis.

After completion of the infusion, patients will undergo the following procedures:

- Vital signs at 1 hour (±5 minutes) postdose
- 12-lead ECG (Day 91 only) at 1 hour (±15 minutes) postdose
- PK and PD sampling at 1 hour (±15 minutes) postdose

Vaccinations should be administered according to Section 6.1.1.1, if applicable, or Appendix I for Japan.

Note: Patients who miss a dose (ie, outside the dosing window or >17 days since last dose) should return to the site for an unscheduled visit 1 week prior to the next scheduled dose in order to receive an additional loading dose. The patient should then resume subsequent dosing visits every 14 days after the loading dose.

6.1.4 End-of-treatment visit in Part A (Week 26)

On Day 182, patients will undergo the following procedures:

- Serum or urine pregnancy test (if applicable)
- Body weight
- Physical examination (full)
- Vital signs
- SLE panel
- Hematology panel
- Coagulation panel
- Clinical chemistry panel
- Urinalysis
- Solicited symptomatic anemia assessment
- QOL assessment (FACIT-Fatigue, EQ-5D-5L, SF-12, PGIS, and PGIC)
- ADAs against BIVV009 (predose) or at ET if patient withdraws early.
- PK and PD sampling
- Disease-related biomarkers
- Record healthcare resource utilization data since previous assessment
- Record concomitant medications/procedures including transfusions
- Adverse event monitoring

After completion of all Part A EOT assessments, a cross-over loading dose will be administered to patients in a blinded manner at the Week 26 visit to initiate dosing in Part B.

6.1.5 Part B extension phase

6.1.5.1 Procedures to be performed every 2 weeks

For patients completing Part A, the following procedures will be performed every 2 weeks (beginning at Week 27) during Part B prior to administration of study drug:

- Hematology panel
- Clinical chemistry panel
- Solicited symptomatic anemia assessment
- Record concomitant medications/procedures including transfusions
- Adverse event monitoring

6.1.5.2 Procedure to be performed every 4 weeks

- Women of childbearing potential will undergo serum or urine pregnancy testing every 4 weeks (beginning at Week 27) during Part B prior to administration of study drug.
- Record healthcare resource utilization data.

6.1.5.3 Procedures to be performed every 3 months

In addition, the following procedures will be performed every 3 months beginning after Week 27 (or as otherwise noted) during Part B prior to administration of study drug:

- Body weight
- Physical examination (brief)
- Vital signs
- Urinalysis
- QOL assessments:
  - FACIT-Fatigue
  - EQ-5D-5L
  - SF-12
  - PGIS
  - PGIC
- 12-lead ECG (once at 3 months [Day 273])
- SLE panel (every 6 months)
- PK, PD, and ADA sampling

At 0 hour, study drug will be infused via an indwelling IV catheter over a period of 60±5 minutes. Patients with underlying cardiopulmonary disease may receive a 2-hour infusion with Sponsor approval. Additionally, study drug infusion may be interrupted or slowed in the event of suspicion of an allergic or anaphylactic reaction.

After completion of the infusion, patients will undergo the following procedures:

- Vital signs at 1 hour (±5 minutes) postdose

- 12-lead ECG (once at 3 months [Day 273]) at 1 hour (±15 minutes) postdose
- PK and PD sampling (every 3 months) at 1 hour (±15 minutes) postdose

6.1.5.4 Additional PK, PD, and ADA sampling

PK and PD samples will continue to be collected at predose and 1 hour (+/- 15 minutes) post-dose on Days 189, 217, and 245.

ADA samples will be collected at predose, on Day 189, 217, and 245.

PK, PD, and ADA samples will be collected if a patient experiences a hematologic breakthrough event (rapid fall in Hgb ≥2 g/dL associated with an increase in LDH/bilirubin and/or decrease in haptoglobin since the last scheduled visit).

6.1.5.5 Infusion of the study drug at patient's home

Home infusions will be performed at pre-selected countries/sites by a healthcare professional caregiver contingent upon completion of training delivered by the Investigator or delegated site staff member only in patients willing to be administered study drug at home, who have been treated for at least 3 months (completed visit Day 273) in Part B and in whom previous on-site infusions were uncomplicated (for the US, the Netherlands, Norway, France, Italy, Austria, Germany, and Spain, specific amendments) (Appendix K). Only patients who do not participate in, or have never participated in undiluted infusions can be considered for home infusion.

6.1.5.6 Infusion of the undiluted study drug

A subset of patients in Part B will be proposed to receive infusions with study drug undiluted with saline solution. Undiluted infusions may begin no sooner than after Week 39 (Day 273) in patients who consent to and without evidence of intolerance to the study drug and who satisfy criteria outlined in Section 4.1. Only patients who do not participate in, or have never participated in home infusion can be considered for undiluted infusions. Each qualified patient is supposed to receive at least one undiluted infusion of BIVV009, however patients are free to continue with undiluted BIVV009 until the end of study. Post-infusion vital signs will be collected for these patients. Patients receiving undiluted infusions of BIVV009 may return to diluted infusions any time, or this may be the Investigator's decision. Patients receiving undiluted BIVV009 will return to diluted infusions if they experience adverse event that in the opinion of the Investigator, may jeopardize patient safety if undiluted infusions are continued. Patients may return to diluted infusions if this AE is considered by the Investigator to be unrelated to BIVV009 and once resolved or stabilized. Hypersensitivity or allergic reactions to study drug will always result in study drug discontinuation and withdrawal from the study.

6.1.5.7 Early termination/end of study/safety follow-up visit

Upon completion of dosing in the study, or if a patient terminates the study early, the following assessments should be completed 9 weeks after administration of the last dose of study drug:

- Serum or urine pregnancy test (if applicable)

- Body weight
- Physical examination (full)
- Vital signs
- SLE panel
- Hematology panel
- Coagulation panel
- Clinical chemistry panel
- Urinalysis
- Solicited symptomatic anemia assessment
- QOL assessments (FACIT-Fatigue, EQ-5D-5L, SF-12, PGIS, and PGIC)
- ADAs against BIVV009
- PK and PD sampling
- Record healthcare resource utilization data since previous assessment
- Record concomitant medications/procedures including transfusions
- Adverse event monitoring

If a patient experiences a hematological breakthrough event during the safety follow-up period, a PK, PD, and ADA sample should be collected at the time of the event.

6.2 STUDY TREATMENT

6.2.1 Drug supplies and accountability

Patients will receive doses of either 6.5 grams or 7.5 grams of BIVV009, depending on their body weight. BIVV009 is supplied to the pharmacy for preparation for infusion in either 10 mL glass vials (18 mg/mL) or 25 mL glass vials (50 mg/mL); for subjects receiving undiluted infusions, BIVV009 is supplied in 25 mL glass vials (50 mg/mL). The Sponsor (or designee) will provide the Investigators (or designees) with adequate quantities of BIVV009 and placebo. BIVV009 drug product will be provided as a sterile, nonpyrogenic, isotonic aqueous solution containing 18 mg/mL or 50 mg/mL BIVV009 with 10 mM sodium phosphate buffer, 140 mM NaCl, 0.02% polysorbate 80 (Tween-80), and water for injection; the pH is 6.1. Each Type 1 glass vial, which has a bromobutyl rubber stopper and an aluminum seal, contains sufficient overfill to account for vial, needle, and syringe loss. Drug product can be used for IV administration only.

BIVV009 placebo is manufactured and packaged in the same configuration as the BIVV009 drug product, but without BIVV009. BIVV009 placebo will be provided as a sterile, nonpyrogenic, isotonic aqueous solution containing 10 mM sodium phosphate buffer, 140 mM NaCl, 0.02% polysorbate 80 (Tween-80), and water for injection; the pH is 6.1. Each Type 1 glass vial, which

has a bromobutyl rubber stopper and an aluminum seal, contains sufficient overfill to account for vial, needle, and syringe loss. BIVV009 placebo can be used for IV administration only.

BIVV009 and BIVV009 placebo should be stored at 2°C to 8°C, protected from light, and kept under secure conditions until immediately before use. It is to be administered via IV infusion according to the instructions given in the Pharmacy Manual for this study.

Preparation and accountability procedures for the investigational product including those associated with home infusions (for the US, the Netherlands, Norway, France, Italy, Austria, Germany, and Spain) specific amendments (Appendix K), whenever applicable, are described in further detail in the Pharmacy Manual.

6.2.2 Patient number and identification

Patients will be assigned unique study numbers that will be used on all study documentation during the course of the trial. For patients who are withdrawn by an Investigator (or designee) or who voluntarily withdraw prematurely from the study, replacement patients will be enrolled only if deemed necessary by the Sponsor. Numbers will not be reused.

6.2.3 Randomization

A

On Day 0, eligible patients will be randomized in a 1:1 ratio to treatment with either BIVV009 or placebo using an Interactive Web-based System (IWRS). Details on the randomization process are available in the eCRF Completion Guidelines.

6.2.4 Dose preparation and administration

B

Qualified pharmacy or clinical staff will prepare each unit dose of study drug for IV infusion. Dose preparation shall proceed as detailed in the Pharmacy Manual for this study. Briefly, placebo or an appropriate number of 10 mL drug product glass vials (each containing 180 mg of BIVV009) or 25 mL drug product glass vials (each containing 1.1 grams of BIVV009) will be pooled and diluted with saline solution to a total volume of 500 mL to administer either a 6.5 gram dose (for patients <75 kg) or a 7.5 gram dose (for patients ≥75 kg), depending on the patient's baseline (Day 0) body weight.

For subjects receiving undiluted infusions, appropriate number of 25 mL drug product glass vials (each containing 1.1 grams of BIVV009) will be pooled to administer either a 6.5 gram dose/130 mL (for patients <75 kg) or a 7.5 gram dose/150 mL (for patients ≥75 kg), depending on the patient's baseline (Day 0) body weight. Details are provided in the Pharmacy Manual.

C

Study drug or placebo will be infused IV by a suitable infusion pump over a period of approximately 60 minutes. (Patients with underlying cardiopulmonary disease may receive a 2-hour infusion with Sponsor approval.) The infusion catheter and tubing will be flushed both immediately before and immediately following completion of the infusion with a sufficient quantity of sterile saline for injection. For each dose, the patient's actual dose, time of dosing, if the administration is being performed at the patient's home (Appendix K), or if study drug is administered in the form of undiluted infusions will be recorded in the source documents and

transcribed onto the eCRFs. The administration location, rate, start time, stop time, any infusion interruptions, and total volume of investigational product actually administered will be recorded in the eCRFs. If an AE occurs during the administration of study drug, the rest of the infusion during which the AE occurred may be slowed or stopped at the discretion of the Investigator or the professional healthcare caregiver in case of home infusions (Appendix K).

Patients must be monitored for acute allergic reactions during infusion and for at least 2 hours after the completion of the first administration of study drug or 1 hour after the completion of each administration of study drug thereafter. Additionally, patients undergoing home infusions will be monitored by the professional healthcare caregiver for 2 hours after the completion of the first home infusion or 1 hour after the completion of each subsequent home infusion (Appendix K). Epinephrine, parenteral diphenhydramine and, for infusions carried at the study site, cardiac resuscitation equipment, must be immediately available in case an allergic reaction or anaphylaxis occurs. Site personnel or professional healthcare caregiver in case of home infusions (Appendix K), must be qualified to detect allergic reactions and anaphylaxis and treat those reactions under the guidance of the PI. The home infusion personnel will be trained in basic life support (cardiopulmonary resuscitation [CPR]) but will not be required to have equipment to perform advanced life support (eg, defibrillators). See Section 6.3.8 for further details regarding additional testing.

Patients should be instructed to report the development of rash, hives, pruritus, flushing, urticaria, etc, that may represent an allergic or hypersensitivity reaction to study drug. If any signs or symptoms of an allergic or anaphylactic reaction are observed during the infusion, administration of study drug must be immediately discontinued and the patient treated as appropriate. In case of home infusion, the investigator has to be notified immediately to guide treatment (Appendix K).

6.2.5 Blinding

Patients and all study staff will be blinded to treatment assignment. Study drug or placebo doses for infusion will be prepared by the pharmacist or qualified site staff in a blinded manner.

Investigators will have direct access to unblinding of treatment through the IWRS in the event of an emergency in which knowledge of treatment assignment would aid in immediate patient care. Should a patient's treatment be unblinded, every effort will be undertaken to protect the blind within the study team. Only a pre-specified group of unblinded team members (safety associate, data manager, biostatistician) will have access to the patient's unblinded data while the study is ongoing.

6.2.6 Concomitant medications

Patients will refrain from participation in any other investigational study drug trial in which receipt of any investigational drug occurred within 5 half-lives or 30 days, whichever is longer, prior to Day 0 and during the entire study.

Treatment with rituximab monotherapy or rituximab combination therapies (eg, with bendamustine, fludarabine, ibrutinib, or cytotoxic drugs) is prohibited.

During Part A, patients will not take any prescription or over-the-counter medications/products until completion of the follow-up assessments, unless prescribed by the Investigator or another physician for the treatment of an AE.

As noted in Section 5.2, concurrent administration of erythropoietin and/or a daily dose of corticosteroids (equivalent to ≤10 mg/day of prednisone) is acceptable provided the patient has been on a stable dose during the previous 3 months; concurrent use of B12, folate and iron supplementation is acceptable provided the patient has been on a stable dose during the previous 4 weeks.

Topical therapies without risk of systemic absorption may be allowed, and non-prescription medications for treatment of minor intercurrent illnesses (headache, viral upper respiratory tract infections, etc) are permitted at the discretion of the PI. Hormonal contraception in female patients is allowed provided patients are receiving stable treatment ≥3 months prior to Screening. Any medication taken by the patient during the study, along with its strength, frequency of dosing, and reason for its use, will be documented in the patient's source data and the eCRF.

6.2.7 Contraception

Women of non-childbearing potential are defined as permanently sterile (ie, due to hysterectomy, bilateral salpingectomy, bilateral oophorectomy) or postmenopausal (defined as at least 12 months post-cessation of menses without an alternative medical cause). Women who are of non-childbearing potential will not be required to use contraception.

Female patients of childbearing potential must be established on (≥3 months prior to Screening) and agree to continue to use the same highly effective methods of birth control (ie, contraceptive measure with a failure rate of <1% per year) in conjunction with male barrier contraception (ie, male condom with spermicide) throughout the study and for 9 weeks after the administration of the last dose of study drug. Highly effective methods of contraception include:

- Intrauterine device (IUD; Mirena®)
- Established use of oral (PO), implanted, transdermal, or hormonal method of contraception associated with inhibition of ovulation
- Bilateral tubal ligation
- Permanent birth control via the Essure procedure

Male patients will be surgically sterile for at least 90 days or when sexually active with female partners of childbearing potential will be required to use a male condom with spermicide (if locally approved for use) throughout the study and for 9 weeks after the last dose of study drug.

Patients who practice true abstinence, because of the patient's lifestyle choice (ie, the patient should not become abstinent just for the purpose of study participation) are exempt from contraceptive requirements. Periodic abstinence (eg, calendar, ovulation, symptothermal, postovulation methods) and withdrawal are not acceptable methods of contraception. If a patient

who is abstinent at the time of signing the ICF becomes sexually active they must agree to use contraception as described above.

For male patients, sexual intercourse with female partners who are pregnant or breastfeeding should be avoided unless condoms are used from the time of administration of the first dose until 9 weeks after administration of the last dose of study drug. Male patients are required to refrain from donation of sperm from Day 0 until 9 weeks after administration of the last dose of study drug.

6.3 STUDY ASSESSMENTS

Please refer to Table 3 and Section 6 (Study Procedures) for an overview of the study procedures and schedule of events. Data will be collected via an eCRF for each randomized patient.
All patients will provide written informed consent before any study-specific assessment is performed. A study-specific assessment is defined as a procedure that is not part of the routine assessments performed for diagnostic purposes or standard care. Screening assessments should occur within 6 weeks prior to administration of the study treatment (ie, on Days -42 to -1).
The immunization status should be confirmed at least 14 days prior to the planned administration of study drug (ie, on or before Day -14).

6.3.1 Demographic data

The date of birth, sex, race, and ethnicity will be documented, as permitted by local regulations.

6.3.2 Medical history

A detailed general medical history of clinically significant diseases and surgeries will be collected during Screening. Additionally, a detailed medical history of the patient's primary CAgD as well as history of all blood transfusions will be reviewed and recorded.

6.3.3 Height, body weight, and vital signs

Height, body weight, and vital signs will be documented. Vital sign measurements (including oral temperature, respiratory rate, supine blood pressure, and pulse rate) will be obtained at the time points specified in Table 3. Vital signs will be measured after the patient has been supine for at least 5 minutes. Vital signs should be measured before any other procedures that may affect pulse rate or blood pressure (eg, blood draws).

6.3.4 Electrocardiograms

A 12-lead ECG will be obtained at the time points specified in Table 3. Patients will be supine for at least 5 minutes prior to obtaining an ECG measurement. The ECG should be performed before any other procedures that may affect heart rate (eg, blood draws).

The Investigator's overall interpretation of the ECG will be recorded and any abnormalities reported.

6.3.5 Physical examinations

Full and brief physical examinations will be performed at the time points specified in Table 3. The time and date of the physical examinations will be recorded in the source document and eCRF, and any clinically significant changes from baseline will be recorded as AEs.

Full physical examinations will consist of the following: general appearance, head and neck, eyes and ears, nose and throat, chest, lungs, heart, abdomen, extremities and joints, lymph nodes, skin, and neurology.

Brief physical examinations will consist of the following: general appearance, chest, lungs, heart, abdomen, and skin.

6.3.6 Immunization review

The patient's medical and vaccination history will be reviewed to ensure that each patient is up-to-date with all required immunizations; if not already vaccinated against *Neisseria meningitidis*, including serogroup B *meningococcus*, where available, *Haemophilus influenzae*, and *Streptococcus pneumoniae* within 5 years of enrollment, these will be administered following informed consent, as applicable, per Table 3 and as described in Section 6.1.1.1 (Screening procedures) or Appendix I for Japan.

6.3.7 Clinical laboratory evaluations

Clinical laboratory evaluations (including hematology panel, clinical chemistry panel, iron panel, erythropoietin assay, coagulation safety panel, SLE panel, virology/serology panel, disease-related biomarkers, and urinalysis) as outlined in Appendix A will be performed at the time points specified in Table 3. Clinical laboratory results will be reviewed by the Investigator and results outside of the reference ranges will be documented and clinical significance noted.

Local laboratory values may be utilized for eligibility evaluation and medical management of the patient, including assessing the hemoglobin criteria necessitating blood transfusions. Detailed information on the allocation of samples for central or local laboratory processing will be available in the Laboratory Manual.

6.3.8 Additional testing in case of hypersensitivity/allergic reaction

If a patient exhibits signs of a hypersensitivity/allergic reaction during study drug administration, the study drug infusion should be stopped immediately, and medical treatment provided, as appropriate.

If a suspected anaphylactic reaction occurs (18), the following labs may be obtained per the discretion of the Investigator, after discussion with the Sponsor Medical Monitor:

- Approximately 30 to 120 minutes after the start of symptoms: blood draw for ADAs (including isotyping), tryptase, IL-6, IL-33, plasma histamine, CICs, and complement levels (CH50). A follow-up tryptase level should be obtained 8 days following the reaction.

- 24-hour urine collection for methylhistamine analysis (ideally collection should be started within 6 hours of onset of symptoms and collected even if the patient is sent to the emergency room; patient can be provided with a container for the collection.

6.4 PHARMACOKINETIC PROCEDURES

6.4.1 Pharmacokinetic blood sample collection and processing

Blood samples for PK analysis of BIVV009 levels and ADAs against BIVV009 will be collected via an indwelling catheter and/or via direct venipuncture from the arm opposite from the site of infusion. Blood samples will be collected at the time points specified in Table 3. If an indwelling catheter is used, saline flushes will be used.

6.4.2 Analytical methodology

Plasma concentrations of BIVV009 will be determined using a validated analytical procedure. Specifics of the analytical methods will be provided in a separate document.

6.5 PHARMACODYNAMIC PROCEDURES

6.5.1 Pharmacodynamic and ADA blood sample collection and processing

Blood samples for ADA and PD analysis of the Complement System Classical Pathway levels (Wieslab assay) will be obtained via an indwelling catheter and/or via direct venipuncture. Blood samples for PD analysis will be collected at the time points specified in Table 3. Complement assays will also be performed on samples, including:

- Total complement (CH50)
- Total C4
- C1q
- C1s

If an indwelling catheter is used, saline flushes will be used.

6.5.2 Analytical methodology

PK, PD, and ADA parameters will be determined using a validated analytical procedure. Specifics of the analytical methods and appropriate matrices for the different parameters will be provided in a separate document.

6.6 SAFETY PROCEDURES

Safety evaluations as needed for medical management of the patient may be repeated at the Investigator's (or designee's) discretion.

Every effort will be made to schedule and perform the procedures in accordance with the nominal time, giving considerations to appropriate posture conditions, practical restrictions, and the other procedures to be performed at the same time point.

The order of priority for scheduling procedures around a time point is (in descending order of priority):

- PK and PD blood sampling
- ADAs against BIVV009
- Vital sign measurements
- ECGs
- Blood and urine samples for clinical laboratory testing
- Physical examinations

6.7 ADVERSE EVENTS AND LABORATORY ABNORMALITIES

It is the responsibility of the Investigator to report all AEs in the eCRF. The AE and SAE reporting period will begin from the time the patient signs the ICF and continue through 9 weeks after administration of the last dose of study drug. Any SAE must be reported within 24 hours of the knowledge of the occurrence to the Study Medical Monitor and the designated Clinical Research Organization's (CRO) Clinical Safety Group. Refer to Section 6.7.4 for further details regarding SAE reporting procedures.

For urgent medical issues in which the study's Medical Director should be contacted, please refer to the study reference manual's Official Study Contact List for complete contact information.

6.7.1 Definition of adverse events

An AE is defined in the International Council for Harmonisation (ICH) Guideline for Good Clinical Practice (GCP) as "any untoward medical occurrence in a patient or clinical investigation subject administered a pharmaceutical product and which does not necessarily have a causal relationship with this treatment" (ICH E6: Section 1.2). An AE can therefore be any unfavorable and unintended sign (including an abnormal laboratory finding, for example), symptom, or disease temporally associated with the use of a medicinal (investigational) product, whether or not considered related to the medicinal (investigational) product.

6.7.1.1 Definition of adverse drug reactions

Adverse drug reactions are defined as all noxious and unintended responses to a medicinal product related to any dose administered. The phrase "responses to a medicinal product" means that a causal relationship between a medicinal product and an AE is at least a reasonable possibility (ie, the relationship cannot be ruled out).

The following categories for determining the causal relationship to the investigational medicinal product are to be used:

Probable (must have first three):

- It follows a reasonable temporal sequence from administration of study drug.
- It cannot be reasonably explained by the known characteristics of the patient's clinical state, environmental or toxic factors, or other modes of therapy administered to the patient.
- It disappears or decreases on cessation or reduction in dose.
- It follows a known pattern of response to the suspected drug.
- It reappears upon re-challenge.

Possible (must have first two):

- It does not follow a reasonable temporal sequence from administration of the drug.
- It may readily have been produced by the patient's clinical state, environmental or toxic factors, or other modes of therapy administered to the patient.
- It does not reappear or worsen when the drug is re-administered.

Unrelated:

An AE will be considered "Unrelated" to the use of the investigational drug if there is not a reasonable possibility that the event has been caused by the product under investigation and does not meet the criteria for drug relationship listed under possible or probable. Factors pointing toward this assessment include, but are not limited to, the lack of reasonable temporal relationship between administration of the drug and the event, the presence of a biologically implausible relationship between the product and the AE (eg, the event occurred before administration of drug), or the presence of a more likely alternative explanation for the AE.

In case of missing causality assessment in the eCRF or SAE reporting form, the event will be regarded as possibly related unless further specified. Any SAE recorded as probably or possibly related will be categorized as "related" for regulatory reporting purposes.

A serious drug reaction is an adverse drug reaction that meets the definition of a serious event (provided below).

6.7.1.2 Definition of serious adverse events

An SAE is defined as any untoward medical occurrence (AE) that at any dose:

- Results in death
- Is life-threatening (patient was at immediate risk of death at the time of the event)
- Requires inpatient hospitalization or prolongation of existing hospitalization
- Results in persistent or significant disability/incapacity
- Is a congenital anomaly/birth defect
- Any other significant medical condition

A hospitalization meeting the regulatory definition for "serious" is any inpatient hospital admission. Any AE that does not meet one of the definitions of serious (ie, important medical events that may not be immediately life-threatening or result in death or hospitalization but may jeopardize the patient or may require interventions to prevent one of the other outcomes listed above [eg, emergency room visit, outpatient surgery, or requires urgent investigation]) may be considered by the Investigator to meet the "other significant medical condition" criterion for classification as an SAE. Examples include allergic bronchospasm, convulsions, and blood dyscrasias.

If a subject develops a Grade 3 or higher allergic reaction per Common Terminology Criteria for Adverse Events (CTCAE) grading or an anaphylactic reaction (see Section 6.3.8) in association with BIVV009 administration, the event should be reported as an SAE.

Exceptions from SAE reporting:

Hospitalization for performing protocol-required procedures or administration of study treatment is not classified as an SAE.

6.7.1.3 Definition of unexpected adverse event/SUSAR

An unexpected AE is any adverse drug event, the specificity or severity of which is not consistent with the current Investigator's Brochure (IB) of BIVV009. Also, reports that add significant information on specificity or severity of a known, already documented AE constitute unexpected AEs. An event more specific or more severe than described in the IB would be considered "unexpected."

A suspected unexpected serious adverse reaction (SUSAR) is a serious adverse reaction, the nature or severity of which is not consistent with the IB. All suspected adverse reactions related to BIVV009 that occur in the concerned clinical trial and that are both unexpected and serious (SUSARs) are subject to expedited reporting as per national regulatory requirements in participating countries.

6.7.2 Clinical adverse events

The Investigator is responsible for ensuring that all AEs observed by the Investigator or reported by the patient are properly captured in the patient's medical records.

The following AE attributes must be assigned by the Investigator:

- Adverse event term
- Dates of onset and resolution
- Seriousness (yes/no)/seriousness criterion
- Severity
- Assessment of relatedness to study drug

- Outcome
  - Recovered/resolved
  - Recovered/resolved with sequelae
  - Not recovered/not resolved
  - Fatal
  - Unknown (only applicable if patient is lost to follow-up)
- Action taken:
  - None
  - Study drug temporarily interrupted
  - Study drug permanently discontinued

During the study, serious and non-serious AEs will be followed until resolved or clinically stable. At the end of the study, all ongoing SAEs will be followed until resolved, stabilized, or returned to baseline.

It will be left to the Investigator's clinical judgment to determine whether an AE is related and of sufficient severity to require the patient's removal from treatment or from the study. A patient may also voluntarily withdraw from treatment due to what he or she perceives as an intolerable AE. If either of these situations arises, the patient should be strongly encouraged to undergo an ET visit assessment and be under medical supervision until symptoms cease or the condition becomes stable.

All clinical AEs encountered during the clinical study will be reported on the AE page of the eCRF, regardless of causality. Severity of AEs will be graded using the CTCAE, version 4.03 (see Appendix B).

If an AE occurs that is not contained in the CTCAE version 4.03, the five-point scale below will be used.

**Grade 1**:	Mild; asymptomatic or mild symptoms; clinical or diagnostic observations only; intervention not indicated
**Grade 2**:	Moderate; minimal, local, or noninvasive intervention indicated; limiting age-appropriate instrumental activities of daily living (ADL)
**Grade 3**:	Severe or medically significant but not immediately life-threatening; hospitalization or prolongation of hospitalization indicated; disabling; limited self-care ADL
**Grade 4**:	Life-threatening consequences; urgent intervention indicated
**Grade 5**:	Death related to AE

6.7.3 Laboratory test abnormalities

Laboratory test value abnormalities that have worsened from baseline should not be reported on the AE page of the eCRF as AEs unless they satisfy one or more of the following conditions for clinical significance:

- Accompanied by clinical symptoms
- Leading to a change in study medication (eg, dose modification, interruption, or permanent discontinuation)
- Requiring a change in concomitant therapy (eg, addition of, interruption of, discontinuation of, or any other change in a concomitant medication, therapy, or treatment

Any laboratory result abnormality fulfilling the criteria for an SAE should be reported as such, in addition to being recorded as an AE in the eCRF.

6.7.4 Serious adverse event reporting - procedures for investigators

All SAEs occurring from the time of informed consent through the final study visit, or 9 weeks after administration of the last dose of study drug, whichever occurs later, must be reported to the Sponsor's designated CRO's Clinical Safety Group within 24 hours of the knowledge of the occurrence. All SAEs that the Investigator considers related to study drug occurring after the follow-up period must be reported directly to the Sponsor.

To report the SAE, the SAE form for the study should be completed and submitted within 24 hours of the site becoming aware of the event to the designated CRO's Clinical Safety Group via email or facsimile (FAX) per the instructions provided on the SAE report form.

The Sponsor (or designee) will report SUSARs to the appropriate regulatory authorities and Investigators according to local law.

6.7.5 Follow-up of adverse events

All serious and non-serious AEs, regardless of whether or not they are assessed as related to study drug, should be followed up during the study until they have resolved, returned to baseline status, or stabilized. If a clear explanation is established, it should be recorded on the eCRF. If there is a unifying diagnosis, the diagnosis should be reported rather than a collection of signs and symptoms.

Ongoing SAEs at the end of study should continue to be followed until the event has resolved, returns to baseline, the condition becomes chronic in nature, stabilizes (in the case of persistent impairment), or the patient dies. Within 24 hours of receipt of any follow-up information, the Investigator must update the eCRF, complete and submit an SAE follow-up form along with any supporting documentation (eg, patient discharge summary or autopsy reports) to the designated CRO's Clinical Safety Group via email or FAX per the instructions provided on the SAE report form.

6.7.6 Follow-up of abnormal laboratory results

In the event of unexplained abnormal laboratory test values, the tests should be repeated immediately and follow-up until they have returned to the normal range and/or an adequate explanation of the abnormality is found. If a clear explanation is established, it should be recorded on the eCRF. Regarding the documentation of abnormal laboratory tests, please refer to Section 6.7.3.

6.7.7 Pregnancy

If a patient becomes pregnant during the study or within 9 weeks of receiving study drug, or if the partner of a patient participating in the study conceives after the patient has received the first dose of study drug and up to 9 weeks after receiving the last dose of study drug, the Investigator should report the pregnancy to the designated CRO's Clinical Safety Group by completing and forwarding a Pregnancy Report Form within 24 hours of being notified.

A patient becoming pregnant while on study drug will immediately be withdrawn from the study, study treatment will be stopped, and early study termination procedures will be performed.

The patient or patient's partner should be followed by the Investigator until the end of the pregnancy. If the pregnancy ends for any reason before the anticipated date, the Investigator should notify the designated CRO's Clinical Safety group by completing and forwarding an updated Pregnancy Report Form. At the end of the pregnancy, the Investigator should document the outcome of the pregnancy and provide a final update via an updated Pregnancy Report Form. If outcome of the pregnancy meets the criteria for immediate classification as an SAE (ie, postpartum complication, spontaneous abortion, stillbirth, neonatal death, or congenital anomaly), the Investigator should follow the procedures for reporting an SAE.

6.7.8 Overdose

An overdose is any dose of study treatment administered to a subject or taken by a subject that exceeds the dose assigned to the subject according to the protocol. Overdoses are not considered AEs and should not be recorded as an AE on the CRF; however, all overdoses must be recorded on an Overdose form and faxed or emailed to the designated CRO's Clinical Safety Group within 24 hours of the site becoming aware of the overdose. An overdose must be reported to the designated CRO's Clinical Safety Group even if the overdose does not result in an AE. If an overdose results in an AE, the AE must be recorded. If an overdose results in an SAE, both the SAE and Overdose forms must be completed and faxed or emailed to the designated CRO's Clinical Safety Group. All study treatment-related dosing information must be recorded on the dosing CRF.

7 DATA ANALYSES AND SAMPLE SIZE

Data from Part A and Part B of this study will be analyzed and reported separately. A separate Statistical Analysis Plan (SAP) for each part of the study will be produced providing full details of all analyses to be performed for Part A and Part B. Should there be any differences between the analyses plan and methodology described within the SAP versus the clinical protocol, the SAP will take precedence.

For the purposes of regulatory submission, an interim analysis of the Part A data will be performed after all patients have completed Part A.

7.1 DESCRIPTION OF OBJECTIVES AND ENDPOINTS

The objectives of the study and the endpoints to be analyzed are described in Section 4.3.
In general, continuous variables will be summarized by descriptive statistics, including: number, mean, median, standard deviation (SD), minimum, and maximum. Categorical variables and response variables will be presented with the number and percentage in each category. Any hypothesis tests will be performed at a two-sided 0.05 significance level. Unless otherwise specified, all data will be presented in patient data listings.

For analyses purposes in Part A, baseline is defined as the last value obtained during Screening immediately prior to administration of the first dose of study drug.

For endpoints involving laboratory parameters, except for hematology panel, results from the central laboratory will be used. Local laboratory values were utilized for Direct Antiglobulin Test (DAT) for, among others, eligibility purposes and hematology panel that allows both determination of eligibility and the medical management of the patient, with assessment of the hemoglobin criteria necessitating blood transfusions.

7.2 DEMOGRAPHICS AND BASELINE DISEASE CHARACTERISTICS

Demographic and other baseline disease characteristics will be summarized using descriptive statistics for the overall population. Data to be tabulated will include, but not be limited to, age, race, medical history, and other disease-specific measures.

7.3 EFFICACY - PART A

7.3.1 Analysis populations

A

Full Analysis Set Population: The Intent-to-Treat (ITT) Population consists of all randomized subjects who received at least 1 dose (including partial dose) of study drug. All subjects in the ITT population will be included in the Full Analysis Set (FAS). For analysis, FAS and the ITT Population are considered exchangeable, but the term FAS will be used in subsequent sections.

Per-Protocol (PP) Population: The PP Population is defined as a subset of FAS who do not have any important protocol deviations impacting their efficacy assessments.

The FAS analyses will be the primary analyses and the PP analyses will be considered as supportive.

The patients in the FAS that are excluded from the PP Population will be listed, together with the reason for exclusion from the PP Population.

7.3.2 Methods of analysis

A

Each patient in the FAS will be classified as a responder or non-responder per the response criteria, and will be compared between treatment groups using Cochran-Mantel-Haenszel test at the significance level of 0.05. The difference in the proportion of responders between groups will be calculated with a 95% exact confidence interval (CI). To assess the hematology component of response, the mean of the non-missing Hgb assessments at the Week 23, Week 25, and Week 26 analysis visits (treatment assessment endpoint) will be used. Visit windows will be used to assign analysis visits, as detailed in the SAP. Patients missing all three analysis visits will be counted as non-responders. Sensitivity analyses will be performed to evaluate the impact by the missing data, when appropriate. Details will be specified in the SAP.

Secondary endpoints, defined as follows, will be tested only when the primary endpoint is statistically significant:

1. Mean change from baseline in Hgb at the treatment assessment endpoint (mean of values at Week 23, 25, and 26)
2. Mean change from baseline in bilirubin (excluding patients with Gilbert's Syndrome) at the treatment assessment endpoint
3. Mean change from baseline in QOL, as assessed by the change in FACIT-fatigue scale scores at the treatment assessment endpoint
4. Mean change from baseline in LDH at the treatment assessment endpoint
5. Incidence of solicited symptomatic anemia at EOT

B

The change from baseline variables will be analyzed using analysis of covariance (ANCOVA) with baseline as covariate. A multiple testing procedure will be specified in the SAP to control the overall Type I error rate among the secondary endpoints.

As a sensitivity analysis, the efficacy analysis will be repeated with patients stratified by previous rituximab therapy and/or cytotoxic therapy versus patients who are naïve to rituximab therapy and/or cytotoxic therapy using the FAS.

Other efficacy assessments will be summarized by overall population using descriptive statistics, proportions and graphically where applicable.

7.4 EFFICACY - PART B

7.4.1 Analysis populations

Intent-to-Treat Population: The ITT Population is defined as all patients who received at least 1 dose of study drug in Part B.

Per-Protocol Population: The PP Population is defined as a subset of ITT Population who do not have any important protocol deviations impacting their efficacy assessments.

7.4.2 Methods of analysis

All endpoints will be analyzed using descriptive statistics, frequency, percentage, 95% CIs, and graphically, as appropriate.

7.5 PHARMACOKINETICS

7.5.1 Analysis population

Part A – All patients who receive at least 1 dose of study drug and have at least 1 evaluable PK sample will be included in the PK analysis population.

Part B – all patients who receive at least 1 dose of study drug and have at least 1 evaluable PK sample during the extension phase will be included in the PK analysis population.

For the purposes of PK parameter analysis, an evaluable patient is defined as a patient who has received at least 1 dose of BIVV009 and has completed the relevant blood sample collections enabling acceptable determination of PK parameters.

7.5.2 Methods of analysis

In general, descriptive statistics including number of observations, mean, SD, median, minimum, and maximum will be presented for continuous parameters. Summary descriptive statistics and individual patient listings will be presented for all PK parameters by time point and study day.

BIVV009 concentrations and individual PK parameter estimates will be listed for each patient and summarized descriptively by time point and study day. Summary descriptive statistics will include the number of observations, arithmetic and geometric means, and their associated CIs, SD, coefficient of variation, median, minimum, and maximum. Mean BIVV009 concentration-versus-time profiles will be plotted.

PK parameters will be log-transformed for these analyses and estimated means, mean differences, and CIs on the log scale will be exponentiated to obtain estimates for geometric means, geometric mean ratios, and CIs, respectively, on the original scale. In addition, untransformed PK parameters will be used to calculate arithmetic means and arithmetic mean ratios.

7.6 PHARMACODYNAMICS

7.6.1 Analysis population

Part A – All patients who receive at least 1 dose of study drug and have at least 1 evaluable PD sample during Part A will be included in the PD analysis population.

Part B – all patients who receive at least 1 dose of study drug and have at least 1 evaluable PD sample during the extension period will be included in the PD analysis population.

7.6.2 Methods of analysis

In general, descriptive statistics including number of observations, mean, SD, median, minimum, and maximum will be presented for continuous parameters. Categorical variables will be presented with the number and percentage in each category. Summary descriptive statistics (absolute values and changes from baseline) and individual patient listings will be presented for all PD parameters by time point and study day.

7.7 IMMUNOGENICITY

7.7.1 Analysis population

Part A – All patients who receive at least 1 dose of study drug and have at least 1 evaluable ADA sample during Part A will be included in the ADA analysis population.

Part B – All patients who receive at least 1 dose of study drug and have at least 1 evaluable ADA sample during the extension period will be included in the ADA analysis population.

7.7.2 Methods of analysis

Methods of analysis will be described in the statistical analysis plan (SAP).

7.8 SAFETY

7.8.1 Analysis population

Part A – all patients who receive at least 1 dose of study drug will be evaluable for the analysis of safety.

Part B – all patients who received at least 1 dose of study drug during the extension period will be evaluable for the analysis of safety.

7.8.2 Methods of analysis

A

Adverse events will be classified using the Medical Dictionary for Regulatory Activities (MedDRA) System Organ Class and Preferred Term. Tabulations of TEAEs and serious TEAEs by frequency, relatedness, and severity will be presented. Patient listings will be provided for SAEs, AEs resulting in discontinuation of the study or study treatment, and all deaths. In addition, specific summary tables will be presented for hemolytic breakthrough, infections (Grade 3 or above), and thrombolytic events.

Changes from baseline in clinical laboratory parameters (except those considered efficacy and PD endpoints), vital signs, and ECG parameters will be summarized over time using descriptive statistics. The number and percentage of patients who have positive ADAs will be presented.

Concomitant medications and physical exam data will be displayed in listings only.

7.9 INTERIM ANALYSES

For the purposes of regulatory submission, an interim analysis of safety and efficacy data will be performed for Part A after all patients have completed Part A. Parts A and B will have separate database locks to enable submission of the BLA/MAA following completion of Part A. Additional interim analyses of Part B data may be performed at the Sponsor's discretion for purposes of regulatory filings, publications, or future planning.

7.10 SAMPLE SIZE CONSIDERATIONS

Approximately 40 patients with primary CAgD who do not have a recent history of transfusion will be randomized.

B

With 40 patients (20 per group), there is 87% power to detect a statistically significant difference of 50% between the BIVV009 group and placebo group if the true response rates are 85% and 35% for the BIVV009 and placebo groups, respectively. This calculation assumes a 2-sided 5%-level test comparing the response rates between the BIVV009 and placebo groups. A 50% improvement over placebo is considered clinically relevant.

7.11 DATA HANDLING AND RECORD KEEPING

Any changes to information in the study progress notes and other source documents will be initialed and dated on the day the change is made by a clinical site staff member authorized to make the change. Changes will be made by striking a single line through erroneous data and clearly entering the correct data (eg, wrong data right data). If the reason for the change is not apparent, a brief explanation for the change will be written adjacent to the change by the clinician.

Patient information will be captured and managed by study sites on eCRFs by a web-based electronic data capture (EDC) tool developed and supported by CRO and approved by the

Sponsor. Data should be entered into the EDC system in a timely manner as outlined within the CRF Completion Guidelines.

Data management will be performed by CRO according to their Standard Operating Procedures (SOPs). The Data Management Plan will be approved by the Sponsor.

7.12 QUALITY CONTROL AND QUALITY ASSURANCE

Quality control procedures will be implemented at each stage of data handling to ensure that all data are reliable and have been processed correctly. Data anomalies will be communicated to the sites for clarification and resolution, as appropriate.

During and/or after completion of the study, quality assurance officers assigned by the Sponsor or the regulatory authorities may wish to perform onsite audits or inspections. The Investigator will be expected to cooperate with any audit or inspection and to provide assistance and documentation (including source data) as requested.

8 ADMINISTRATIVE ASPECTS

8.1 CHANGE IN PROTOCOL

There will be no alterations in the protocol without agreement between the Sponsor and the Investigator.

There will be no alterations in the protocol affecting patient safety without the express written approval of the Sponsor, Investigator, and the Institutional Review Boards/Ethics Committees (IRBs/ECs) (see form FDA 1572).

All protocol amendments must be submitted to the IRBs/ECs and regulatory authorities if required by local law. Protocol modifications that affect patient safety, the investigational scope, or the scientific quality of the study must be approved by the IRB/EC before implementation of such modifications to the conduct of the study. If required by local law, such modifications must also be approved by the appropriate regulatory agency before implementation. However, the Sponsor may, at any time, amend this protocol to eliminate an apparent immediate hazard to a patient. In this case, the appropriate regulatory authorities will be notified subsequent to the modification.

8.2 SITE INITIATION VISIT/INVESTIGATOR MEETING

Prior to the start of the clinical study, the representative(s) of the Sponsor will meet with the Investigator and appropriate clinical staff to familiarize the Investigator and clinical staff with the clinical protocol and the materials necessary for conducting the clinical study.

8.3 DISCLOSURE

All information provided regarding the study, as well as all information collected/documented during the study will be regarded as confidential. The Investigator (or designee) agrees not to disclose such information in any way without prior written permission from the Sponsor.

Any publication of the results, either in part or in total (eg, articles in journals or newspapers, oral presentations, abstracts) by the Investigator or their representative(s), shall require prior notification and review, within a reasonable time frame, by the Sponsor, and cannot be made in violation of the Sponsor's confidentiality restrictions or to the detriment of the Sponsor's intellectual property rights.

Sponsor will register the study and post study results regardless of outcome on a publicly accessible website in accordance with the applicable laws and regulations.

8.4 MONITORING

The Monitor has the responsibility to familiarize the Investigator(s) and the entire center staff involved in the study with all study procedures including the administration of study drug. The

Monitor will monitor the study conduct, proper eCRF and source documentation completion and retention, and accurate study drug accountability. To this end, the Monitor will visit the clinical site at suitable intervals and be in frequent contact through verbal and written communication. It is essential that the Study Monitor has access to all documents (related to the study and the individual participants) at any time these are requested. In turn, the Monitor will adhere to all requirements for patient confidentiality as outlined in the ICF. The Investigator and Investigator's staff will be expected to cooperate with the Study Monitor, to be available during a portion of the monitoring visit to answer questions, and to provide any missing information.

Monitoring details describing strategy (eg, risk-based initiatives in operations and quality such as Risk Management and Mitigation Strategies and Analytical Risk-Based Monitoring), methods, responsibilities and requirements, including handling of noncompliance issues and monitoring techniques (central, remote, or on-site monitoring) are provided in separate study documents.

8.5 ETHICAL ASPECTS

The Sponsor, CRO, and the Investigator must comply with all instructions, regulations, and agreements in this protocol and applicable ICH and GCP guidelines, and conduct the study according to local regulations. The Investigator may delegate responsibilities for study-related tasks where appropriate to individuals sufficiently qualified by education, training, and experience, in accordance with applicable ICH and GCP guidelines. The Investigator should maintain a list of the appropriately qualified persons to whom significant study-related duties have been delegated. The Investigator is responsible for supervising those individuals and for implementing procedures to ensure the integrity of the tasks performed and any data generated.

8.5.1 Declaration of helsinki/good clinical practice

The Declaration of Helsinki is the accepted basis for clinical study ethics, and must be fully followed and respected by all engaged in research on human beings. Any exceptions must be justified and stated in the protocol. The latest version of the Declaration of Helsinki is available under www.wma.net/en/30publications/10policies/b3/index.html.pdf. Additionally, it is the responsibility of all engaged in research on human beings to ensure that the study is performed in accordance with the international GCP standards and according to all local laws and regulations concerning clinical studies.

8.5.2 Patient information and informed consent

It is the responsibility of the Investigator to obtain written informed consent from each patient participating in this study, after adequate explanation of aim, importance, anticipated benefits, and potential hazards and consequences of the study according to applicable local laws. Written informed consent must be obtained before any study-specific procedures are performed. It must be also explained to the patient that he/she is completely free to refuse to enter the study or to withdraw from it at any time for any reason without incurring any penalty or withholding of treatment on the part of the Investigator.

With the declaration of consent the patient agrees that data on his/her medical history are recorded within the framework of the clinical study and that they are transferred to the Sponsor in a pseudo-anonymized manner. Patients will be informed that their race and ethnicity will be collected and will be used during analysis of study results.

The patient also agrees to allow the monitor/auditor/health authorities to verify the collected patient data against the patient's original medical records for the purpose of source data verification.

The ICF – personally signed and dated by the patient and the Investigator – must be kept on file by the Investigator(s) and documented in the eCRF and the patient's medical records. The Investigator confirms to the Sponsor to obtain the written informed consent from any patient before participating in the study.

If new safety information results in significant changes in the risk/benefit assessment, the consent form should be reviewed and updated if necessary. All patients (including those already being treated) should be informed of the new information and must give their written informed consent to continue in the study.

If the family doctors are informed of their patients' participation in the clinical study, this should be mentioned in the consent form.

8.6 INDEPENDENT ETHICS COMMITTEE/INSTITUTIONAL REVIEW BOARD AND REGULATORY AUTHORITIES

It is the responsibility of the Sponsor to obtain and maintain independent approval from the applicable Regulatory Authorities to conduct the study in accordance with applicable regulatory requirements. It is the responsibility of the Sponsor to ensure that a positive opinion from the ECs/IRBs to conduct the study in accordance with applicable regulatory requirements is in place.

8.7 RECORDS

Data collected at Screening and during the study will be recorded in the patient's source documents and retained at the study site for all patients who sign the ICF. Patients who are enrolled in the study will have their data retained in the source documents at the site and also have their data entered into the eCRF. To maintain confidentiality, the patients will be identified only by screening and patient numbers.

The completed eCRFs will be transferred to the Sponsor (or designee). Copies of each source document will be retained by the Investigator (or designee). A compact disk containing the site eCRF data will be provided to the site at the completion of the study. All source documents, records, and reports will be retained by the clinical site in accordance with 21 CFR 312.62(c). The minimum retention time for study records will meet the strictest standard applicable to that site, as dictated by any institutional requirements or local, national, or regional laws or regulations. Prior to proceeding with destruction of records, the Investigator must notify the Sponsor in writing and receive written authorization from the Sponsor to destroy study records.

In addition, the Investigator must notify the Sponsor of any changes in the archival arrangements including but not limited to archival at an offsite facility or transfer of ownership if the Investigator leaves the site.

All primary data, or copies thereof (eg, laboratory records, eCRFs, data sheets, correspondence, photographs, and computer records), which are a result of the original observations and activities of the study and are necessary for the reconstruction and evaluation of any study report, will be retained in the clinical site archives.

9 REFERENCES

1. Berentsen S, Tjonnfjord GE. Diagnosis and treatment of cold agglutinin mediated autoimmune hemolytic anemia. Blood Rev. 2012;26(3):107-15.

2. Berentsen S, Beiske K, Tjonnfjord GE. Primary chronic cold agglutinin disease: an update on pathogenesis, clinical features and therapy. Hematology. 2007;12(5):361-70.

3. Petz LD. Cold antibody autoimmune hemolytic anemias. Blood Rev. 2008;22(1):1-15.

4. Berentsen S. How I manage cold agglutinin disease. Br J Haematol. 2011;153(3):309-17.

5. Swiecicki PL, Hegerova LT, Gertz MA. Cold agglutinin disease. Blood. 2013;122(7):1114-21.

6. Arthold C, Skrabs C, Mitterbauer-Hohendanner G, Thalhammer R, Simonitsch-Klupp I, Panzer S, et al. Cold antibody autoimmune hemolytic anemia and lymphoproliferative disorders: a retrospective study of 20 patients including clinical, hematological, and molecular findings. Wien Klin Wochenschr. 2014;126(11-12):376-82.

7. Berentsen S. Complement, cold agglutinins, and therapy. Blood. 2014;123(26):4010-2.

8. Berentsen S. How I manage patients with cold agglutinin disease. Br J Haematol. 2018;181(3):320-30.

9. Mullins M, Jiang X, Bylsma LC, Fryzek JP, Reichert H, Chen EC, et al. Cold agglutinin disease burden: a longitudinal analysis of anemia, medications, transfusions, and health care utilization. Blood Adv. 2017;1(13):839-48.

10. Barcellini W, Fattizzo B, Zaninoni A, Radice T, Nichele I, Di Bona E, er al. Clinical heterogeneity and predictors of outcome in primary autoimmune hemolytic anemia: a GIMEMA study of 308 patients. Blood. 2014;124(19):2930-6.

11. Röth A, Bommer M, Hüttmann A, Herich-Terhürne D, Kuklik N, Veronika L, et al. Complement inhibition with eculizumab in patients with cold agglutinin disease (CAD): Results from a prospective Phase II trial (DECADE Trial). Blood. 2015;128(23):274.

12. Broome C, Cunningham JM, Mullins M, Jiang X, Bylsma L, Fryzek J, et al. Incidence of thromboembolic events is increased in a retrospective analysis of a large cold agglutinin disease (CAD) cohort. Blood. 2017;130(Suppl 1):928.

13. Berentsen S. Cold agglutinin disease. Hematology Am Soc Hematol Educ Program. 2016(1):226-31.

14. Randen U, Troen G, Tierens A, Steen C, Warsame A, Beiske K, et al. Primary cold agglutinin-associated lymphoproliferative disease: a B-cell lymphoma of the bone marrow distinct from lymphoplasmacytic lymphoma. Haematologica. 2014; 99(3):497-504.

15. Berentsen S, Ulvestad E, Langholm R, Beiske K, Hjorth-Hansen H, Ghanima W, et al. Primary chronic cold agglutinin disease: a population based clinical study of 86 patients. Haematologica. 2006;91(4):460-6.

16. Jäger U, Gilbert JC, Panicker S, Parry GC, Fillitz M, Schenk T, et al. The anti C1s complement antibody TNT009 induces rapid complete remissions of anaemia in patients with primary cold agglutinin disease. 21st European Haematology Association (EHA) Conference. 2016;Abstract LB2237, 341.

17. Jaeger U, D'Sa S, Schoergenhofer C, Bartko J, Sillaber C, Jilma-Stohlawetz P, et al. Long term efficacy, safety and PK/PD profile of the anti-C1s antibody (BIVV009) in primary cold agglutinin disease patients. Blood. 2017;130(Suppl 1):703.

18. Simons FE. Anaphylaxis. J Allergy Clin Immunol. 2010;125(2 Suppl 2):S161-81.

INVESTIGATOR AGREEMENT

Study Drug: BIVV009

Protocol: BIVV009-04

A PHASE 3, RANDOMIZED, DOUBLE-BLIND, PLACEBO-CONTROLLED STUDY TO ASSESS THE EFFICACY AND SAFETY OF BIVV009 IN PATIENTS WITH PRIMARY COLD AGGLUTININ DISEASE WITHOUT A RECENT HISTORY OF BLOOD TRANSFUSION

Version 8

04 November 2020

I have read the foregoing protocol and agree to conduct the study as described herein.

Principal Investigator (signature)	Date

Principal Investigator (print)

10 APPENDICES

Appendix A CLINICAL LABORATORY EVALUATIONS

Clinical Chemistry Panel:
Alanine aminotransferase
Albumin
Alkaline phosphatase
Aspartate aminotransferase
Blood urea nitrogen
Calcium
Chloride
Creatinine
Glucose
Haptoglobin
Lactate dehydrogenase (LDH)
Potassium
Sodium
Total bilirubin
Direct bilirubin
Indirect bilirubin
Total protein
Uric acid

Hematology Panel:
Hematocrit
Hemoglobin
Mean corpuscular hemoglobin
Mean corpuscular hemoglobin concentration
Mean corpuscular volume
Platelet count
Red blood cell (RBC) count
RBC distribution width
Reticulocyte count
White blood cell (WBC) count
WBC differential (absolute):
- Basophils
- Eosinophils
- Lymphocytes
- Monocytes
- Neutrophils

Urinalysis:
Bilirubin
Color and appearance
Glucose
Ketones
Leukocyte esterase
Nitrite
Occult blood
pH and specific gravity
Protein
Urobilinogen
Microscopic exam including
- bacteria, casts, crystals, epithelial cells, RBCs, and WBCs (if protein, leukocyte esterase, nitrite, or blood is positive)

Pregnancy Test
(for women of childbearing potential): serum test during Screening, serum or urine test at all other time points

Hepatic Panel/HIV:
Hepatitis B surface antigen
Hepatitis C virus antibody
Human immunodeficiency virus antibody

Pharmacodynamic (PD) Assays:
Complement System Classical Pathway (Wieslab-CP)
CH50
Total C4
C1q
C1s

Systemic Lupus Erythematosus Panel:
Antinuclear antibodies (ANA)
multiplex with double stranded DNA
Anti-La/SSB antibody (SS-B)
Anti-ribonucleoprotein antibody (RNP)
Anti-Smith antibody (Sm)
Anti-Ro/SSA antibody (SS-A)
Anti-scleroderma antibody (Scl-70)
Anti-Chromatin antibody
Anti-Jo-1 antibody
Anti-Centromere B antibody
Circulating immune complexes (CIC)

Coagulation Panel:
Prothrombin time or the international ratio of PT (PT-INR)
Activated partial thromboplastin time (aPTT)
D-dimer
Thrombin-antithrombin assay

Iron Panel and Erythropoietin
Erythropoietin
Serum iron
Total iron binding capacity (TIBC)
Transferrin saturation
Ferritin

Gilbert's Syndrome Test
UGT1A1 gene

Disease-Related Biomarkers
DAT (polyspecific, anti-IgG & anti-C3d)
LDH isoforms
Cold agglutinin (CAg) titer
Ig subsets (IgA, IgD, IgG, IgM)
Vaccine titers
CAg thermal amplitude

For information on the anti-drug antibody (ADA) and PK evaluations please refer to the Laboratory Manual.

Detailed information on the allocation of samples for central or local laboratory processing is available in the Laboratory Manual.

Appendix B CI-CTC VERSION 4.03 (CTCAE V4.03)

https://evs.nci.nih.gov/ftp1/CTCAE/CTCAE_4.03/CTCAE_4.03_2010-06-14_QuickReference_8.5x11.pdf

Appendix C FUNCTIONAL ASSESSMENT OF CHRONIC ILLNESS THERAPY (FACIT)-FATIGUE SCALE

FACIT Fatigue Scale (Version 4)

Below is a list of statements that other people with your illness have said are important. **Please circle or mark one number per line to indicate your response as it applies to the past 7 days.**

		Not at all	A little bit	Some-what	Quite a bit	Very much
HI7	I feel fatigued	0	1	2	3	4
HI12	I feel weak all over	0	1	2	3	4
An1	I feel listless ("washed out")	0	1	2	3	4
An2	I feel tired	0	1	2	3	4
An3	I have trouble starting things because I am tired	0	1	2	3	4
An4	I have trouble finishing things because I am tired	0	1	2	3	4
An5	I have energy	0	1	2	3	4
An7	I am able to do my usual activities	0	1	2	3	4
An8	I need to sleep during the day	0	1	2	3	4
An12	I am too tired to eat	0	1	2	3	4
An14	I need help doing my usual activities	0	1	2	3	4
An15	I am frustrated by being too tired to do the things I want to do	0	1	2	3	4
An16	I have to limit my social activity because I am tired	0	1	2	3	4

Appendix D EQ-5D-5L

EQ-5D-5L

Sample

Health Questionnaire

English version for the USA

USA (English) © 2009 EuroQol Group EQ-5D™ is a trade mark of the EuroQol Group

Under each heading, please check the ONE box that best describes your health TODAY.

**MOBILITY**	
I have no problems walking	❑
I have slight problems walking	❑
I have moderate problems walking	❑
I have severe problems walking	❑
I am unable to walk	❑
**SELF-CARE**	
I have no problems washing or dressing myself	❑
I have slight problems washing or dressing myself	❑
I have moderate problems washing or dressing myself	❑
I have severe problems washing or dressing myself	❑
I am unable to wash or dress myself	❑
**USUAL ACTIVITIES** *(e.g. work, study, housework, family or leisure activities)*	
I have no problems doing my usual activities	❑
I have slight problems doing my usual activities	❑
I have moderate problems doing my usual activities	❑
I have severe problems doing my usual activities	❑
I am unable to do my usual activities	❑
**PAIN / DISCOMFORT**	
I have no pain or discomfort	❑
I have slight pain or discomfort	❑
I have moderate pain or discomfort	❑
I have severe pain or discomfort	❑
I have extreme pain or discomfort	❑
**ANXIETY / DEPRESSION**	
I am not anxious or depressed	❑
I am slightly anxious or depressed	❑
I am moderately anxious or depressed	❑
I am severely anxious or depressed	❑
I am extremely anxious or depressed	❑

sample

2

USA (English) © 2009 EuroQol Group EQ-5D™ is a trade mark of the EuroQol Group

- We would like to know how good or bad your health is TODAY.
- This scale is numbered from 0 to 100.
- 100 means the best health you can imagine.
  0 means the worst health you can imagine.
- Mark an X on the scale to indicate how your health is TODAY.
- Now, please write the number you marked on the scale in the box below.

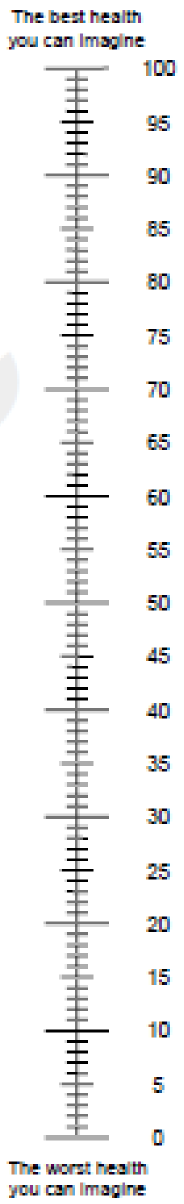

YOUR HEALTH TODAY = ☐

Sample

USA (English) © 2009 EuroQol Group EQ-5D™ is a trade mark of the EuroQol Group

Appendix E 12-ITEM SHORT FORM SURVEY (SF-12)

Your Health and Well-Being

This survey asks for your views about your health. This information will help keep track of how you feel and how well you are able to do your usual activities. *Thank you for completing this survey!*

For each of the following questions, please mark an ☒ in the one box that best describes your answer.

1. In general, would you say your health is:

Excellent	Very good	Good	Fair	Poor
☐ 1	☐ 2	☐ 3	☐ 4	☐ 5

2. The following questions are about activities you might do during a typical day. Does your health now limit you in these activities? If so, how much?

	Yes, limited a lot	Yes, limited a little	No, not limited at all
a Moderate activities, such as moving a table, pushing a vacuum cleaner, bowling, or playing golf	☐ 1	☐ 2	☐ 3
b Climbing several flights of stairs	☐ 1	☐ 2	☐ 3

3. During the <u>past 4 weeks</u>, how much of the time have you had any of the following problems with your work or other regular daily activities <u>as a result of your physical health?</u>

	All of the time	Most of the time	Some of the time	A little of the time	None of the time
a Accomplished less than you would like	☐ 1	☐ 2	☐ 3	☐ 4	☐ 5
b Were limited in the kind of work or other activities	☐ 1	☐ 2	☐ 3	☐ 4	☐ 5

4. During the <u>past 4 weeks</u>, how much of the time have you had any of the following problems with your work or other regular daily activities <u>as a result of any emotional problems</u> (such as feeling depressed or anxious)?

	All of the time	Most of the time	Some of the time	A little of the time	None of the time
a Accomplished less than you would like	☐ 1	☐ 2	☐ 3	☐ 4	☐ 5
b Did work or other activities less carefully than usual	☐ 1	☐ 2	☐ 3	☐ 4	☐ 5

5. During the past 4 weeks, how much did pain interfere with your normal work (including both work outside the home and housework)?

Not at all	A little bit	Moderately	Quite a bit	Extremely
☐ 1	☐ 2	☐ 3	☐ 4	☐ 5

6. These questions are about how you feel and how things have been with you during the past 4 weeks. For each question, please give the one answer that comes closest to the way you have been feeling. How much of the time during the past 4 weeks…

	All of the time	Most of the time	Some of the time	A little of the time	None of the time
a Have you felt calm and peaceful?	☐ 1	☐ 2	☐ 3	☐ 4	☐ 5
b Did you have a lot of energy?	☐ 1	☐ 2	☐ 3	☐ 4	☐ 5
c Have you felt downhearted and depressed?	☐ 1	☐ 2	☐ 3	☐ 4	☐ 5

7. During the past 4 weeks, how much of the time has your physical health or emotional problems interfered with your social activities (like visiting with friends, relatives, etc)?

All of the time	Most of the time	Some of the time	A little of the time	None of the time
☐ 1	☐ 2	☐ 3	☐ 4	☐ 5

Thank you for completing these questions!

Appendix F PATIENT'S GLOBAL IMPRESSION OF CHANGE (PGIC)

Since the start of the study, my overall status is:

√ one box only:

[1] ☐ Very much improved

[2] ☐ Much improved

[3] ☐ Minimally improved

[4] ☐ No change

[5] ☐ Minimally worse

[6] ☐ Much worse

[7] ☐ Very much worse

(US/English)

SAMPLE

Appendix G HEALTHCARE RESOURCE UTILIZATION

Healthcare resource utilization data will be collected during the Screening/Observation Period; on Day 0; on Days 21, 49, 77, 105, 133, and 161; at EOT for Part A; every 4 weeks during Part B; and at the ET/EOS visit.

For screening, please provide the healthcare resource utilization data for the past 6 months prior to the start of the Screening/Observation period.

Has the patient had any additional non-study outpatient visits, emergency room visits, or inpatient hospitalizations related to their cold agglutinin disease during the last 4 weeks?

Yes or No

If yes, please provide the information below including the number of visits.

		Yes/No	How Many
A.	Extra or unscheduled (non-study) visit to the office of the study doctor	_______	_______
B.	Visit to a generalist doctor	_______	_______
C.	Visit to a specialist doctor	_______	_______
D.	Visit to another healthcare professional (eg, nurse, therapist)	_______	_______
E.	Complementary/alternative visit (eg, homeopathic, herbalist)	_______	_______
F.	Visit to an urgent care or walk-in clinic (excluding a hospital emergency room)	_______	_______
G.	Visit to a hospital emergency room<br>Reason(s) for the visit (diagnosis): ____________________________	_______	_______
H.	Use of an ambulance service	_______	_______
I.	Hospitalization		

Date of hospital admission ____________________ and date of discharge ________________

Discharge diagnosis: ______________________________

Was the patient admitted to the intensive care unit (ICU)? Yes or No

If yes, please provide dates or duration in the ICU.

Date of ICU admission: ___________________ and date of ICU discharge: __________________

OR

Duration of ICU stay: __________________

Appendix H PATIENT'S GLOBAL IMPRESSION OF [FATIGUE] SEVERITY

See below for an example.

Protocol No: ___________ Patient ID ___________ Visit Date ___________ Visit ID: ___________

Patient Global Impression of Severity (PGIS)

1. Please choose the response below that best describes the severity of your fatigue over the past week. (Select ONE box)

None	☐ 1
Mild	☐ 2
Moderate	☐ 3
Severe	☐ 4
Very Severe	☐ 5

Appendix I VACCINE SCHEDULE FOR PATIENTS IN JAPAN ONLY

The vaccine schedule for patients in Japan who do not have documented vaccination in the 5 years prior to enrollment is provided below.

1. Meningococcal conjugate vaccine (MenACWY) (2-dose series 8 weeks apart)
   a) The first dose should be given on Day -42 when possible and no later than Day -28 during the Screening/Observation Period.
   b) The second dose should be given 8 weeks after the first dose, between Day 14 and Day 32 in Part A.
2. Pneumococcal vaccine (PPSV23)
   a) Single dose should be given on Day -42 when possible and no later than Day -28 during the Screening/Observation Period.

Notes: If necessary, vaccinations may be administered at an unscheduled visit per patient and vaccine availability.

For vaccination requirements in countries other than Japan, please vaccinate per local vaccine availability and vaccination guidelines for patients with complement deficiency as discussed in Section 6.1.1.1.

Appendix J PROTOCOL AMENDMENT HISTORY

The "Protocol Amendment Summary of Changes Table" for the current amendment is located at the start of this document (after title page).

History of previous protocol amendments is as follows:

Document	Country/countries impacted by amendment	Date
Protocol Version 7	All	07 JULY 2020
Protocol Version 6	All	15 OCTOBER 2019
Protocol Version 5	All	19 JULY 2018
Protocol Version 4	Not implemented	29 JUNE 2018
Protocol Version 3.1	Japan	27 MARCH 2018
Protocol Version 3	All, except Japan	21 MARCH 2018
Protocol Version 2	Not implemented	NA
Protocol Version 1.6	Italy	29 JANUARY 2018
Protocol Version 1.5	Belgium	10 JANUARY 2018
Protocol Version 1.4	France	15 DECEMBER 2017
Protocol Version 1.3	Norway	06 DECEMBER 2017
Protocol Version 1.2	UK	29 NOVEMBER 2017
Protocol Version 1.1	All	09 SEPTEMBER 2017
Protocol Version 1 (original)	All	24 AUGUST 2017

Protocol Version 7:

This is a global amendment to protocol Version 6 and is regarded as substantial amendment based on the criteria set forth in Article 10(a) of Directive 2001/20/EC of the European Parliament and the Council of the European Union.

The primary reasons for this amendment to Protocol BIVV009-04 (Cadenza), Version 7 are:

- To remove requirements for cardiac resuscitation equipment during home infusion visits in countries already identified for the home infusion sub-study (the US, the Netherlands, Norway, France, Italy, Austria, Germany, and Spain) as the risk of occurrence of emergency situations during infusions at home has been minimized by authorizing enrollment of patients without history of hypersensitivity reactions to study drug, and assistance by personnel trained in basic life support will be implemented,
- To introduce the option of infusion with undiluted solution of BIVV009 in a subset of patients in Part B of the study,
- To clarify the order of assessments post dose of IMP,
- To add clarification regarding action taken with the IMP in case of allergic or hypersensitivity reactions.

PROTOCOL AMENDMENT SUMMARY OF CHANGES

Section # and name	Description of change	Brief rationale
Title Page, Approval of the Protocol, Protocol Amendment Summary of Changes Table	Document formatting revisions.	The change was made to reflect the update of the amended protocol version 7.
Synopsis: Objectives	Exploratory Objective in Part B to describe the safety of undiluted infusions with BIVV009 was added.	The changes were made in relation to the introduction of undiluted infusion of the IMP for a subset of patients.
Synopsis: Test Product(s), Dose, and Mode of Administration	At certain visits during Part B administration of the study drug may be provided in the form of undiluted solution to a subset of patients was added.	
Synopsis: Safety Outcome Measures	For patients receiving undiluted infusions, safety assessments will additionally include AEs with onset within 24 hours of the infusion was added.	
Section 2.2 Background and Study Rationale	Information about undiluted solution and its benefits to patients and pharmacy staff was added.	
Section 2.2.4 Potential Risks and Benefits and Appendix K	Potential risks associated with dosing at home were further detailed.	
Section 2.2.4 Potential Risks and Benefits	Benefit and risk information for the inclusion of undiluted infusions was added.	
Section 3.5 Exploratory Objective (Part B)	Exploratory objective to describe the safety of undiluted infusions with BIVV009 was added.	
Section 4.1 Study Design	Criteria for qualifying patients in Part B to receive infusions with study drug undiluted with saline solution were added.	
Section 4.1 Study Design	Updated the responder definition in Table 2 for Part A.	The responder definition was not changed, rather the update to the language was made to make the definition clearer for each individual component.
4.3.5 Safety Endpoints	For patients receiving undiluted infusions, safety assessments will additionally include AEs with onset within 24 hours of the infusion was added.	The change was made in relation to the introduction of undiluted infusion of the IMP for a subset of patients.
Section 5.2 Exclusion Criteria	Reference to Section 4.1 eligibility criteria for undiluted infusion was added.	The change was made in relation to the introduction of undiluted infusion of the IMP for a subset of patients.
Section 5.3 Removal of Patients from Study Participation, and Study Suspension and Stopping Rules	Hypersensitivity or allergic reactions including anaphylaxis to study drug were added to the list of withdrawal criteria. The last point in the list of reasons for study discontinuation regarding hypersensitivity reactions during home infusions was removed.	The change was not due to new safety concern but rather to clarify that any new hypersensitivity/allergic and/or anaphylactic reactions to study drug should lead to study drug discontinuation as hypersensitivity is a contraindication to BIVV009 administration.

Section # and name	Description of change	Brief rationale
Section 6.1.5.3 Procedures to be Performed Every 3 Months	Day of ECG postdose in Part B changed from 217 to 273.	Correction of a typo.
Section 6.1.5.5. Infusion of Study Drug at Patient's Home and Appendix K	Participation in undiluted infusions was added as a non-eligibility criteria for home infusion.	The change was made to precise home infusion eligibility criteria.
Section 6.1.5.6 Infusion of the Undiluted Study Drug	This is a new section that was added to amended protocol version 7.	The change was made in relation to the introduction of undiluted infusion of the IMP for a subset of patients.
Section 6.1.5.7 Early Termination/End of Study/Safety Follow-up Visit	The title and content in this section were previously found in Section 6.1.5.6 in amended protocol version 6 and the title and content in this section has been moved its current location in amended protocol version 7.	The change was made to reflect the update in formatting of sections the amended protocol version 7.
Section 6.2.1 Drug Supplies and Accountability	Information about the IMP BIVV009 kit configuration supplied for subjects receiving undiluted infusions was added.	The change was made in relation to the introduction of undiluted infusion of the IMP for a subset of patients.
Section 6.2.4 Dose Preparation and Administration and Appendix K	Removal of the requirement for cardiac resuscitation equipment during home infusion visits (for the countries identified as per protocol version 6 eg, US, the Netherlands, Norway, France, Italy, Austria, Germany, and Spain).	Only patients treated for the minimum of 3 months with BIVV009 and who did not exhibit hypersensitivity reaction to study drug are eligible for home infusions. Rescue medications will be immediately available for use as per the PI's guidance during home infusion visits and the home infusion personnel will be trained in basic life support (cardiopulmonary resuscitation [CPR]). As a result, the requirement for cardiac resuscitation equipment during home infusion was removed.
Section 6.2.4 Dose Preparation and Administration	Information for dose preparation and administration for subjects receiving undiluted infusions was added.	The change was made in relation to the introduction of undiluted infusion of the IMP for a subset of patients.
Section 6.3.3 Height, Body Weight, and Vital Signs	It was specified that 1-hour post-infusion vital signs should be measured before blood draws.	The order of post-infusion assessments was clarified to make sure that procedures that may impact vital signs, ie, blood draws are done at the end of the office visit.
Section 7.3.1 Analysis Populations	Regarding ITT population description addition of the term "Full Analysis Set" has been added and will be used instead of "ITT Population".	The change was made to align with the language in the SAP.
Section 7.3.2 Methods of Analysis	Regarding ITT population description addition of the term "Full Analysis Set" has been added and will be used instead of "ITT Population".	The change was made to align with the language in the SAP.
Appendix K	Editorial changes to the Appendix K.	This change was made to remove redundant text and correct existing inconsistencies.

In addition, other minor editorial changes (eg, grammatical, stylistic, and minor typographical error corrections) were implemented throughout the protocol, as well as an administrative change in (Section 8.4).

Protocol Version 6:

Section # and Name	Description of Change	Brief Rationale
Synopsis, Section 2.2, Section 2.2.4, Section 3.5, Section 4.1 (Part-B), Section 4.3.4, Section 4.3.5, Section 5.2, Section 5.3, Section 6.1 Table-3, Section 6.1.5.5, Section 6.2.1, Section 6.2.4, Appendix K (Appendix K) and Appendix L (Appendix L).	Home infusion will be performed by a healthcare professional caregiver, applicable to pre specified countries (the US, the Netherlands, Norway, France, Italy, Austria, Germany, Spain), and sites only. Qualifying patients who consent for home infusions will have study drug infused at their homes at selected visits, supported by a healthcare professional caregiver.	The primary reasons for this amendment to Protocol BIVV009-04 (Cadenza) are<br>- to provide home infusion facilities to the patients (the option of home infusions may be beneficial for CAgD patients who often are an elderly population with other comorbidities. This procedure is intended to characterize the safety and patient satisfaction with the convenience of home infusions).<br>- to add new exploratory objective of immunogenicity,<br>- to introduce additional time points when samples for ADAs against BIVV009 are collected (additional ADA samplings will help characterize immunogenicity of BIVV0009),<br>- to specify that predose PD back-up samples will be used to assess immunogenicity in patients who consented to future use of samples, - to introduce additional time points for SLE panel in Part B (As duration of participation in Part B is patient-specific, it has been specified that surveillance with systemic lupus erythematosus (SLE) serologic testing will be done every 6 months),<br>- to clarify PK/PD sampling schedules for Part-B of the study (to fix the inconsistency concerning PK and PK sampling schedule during Part B between Schedule of Study Procedures table and body of the protocol)<br>- to introduce a correction in Appendix A.
Synopsis, Section 3.2, Section 3.4, Section 4.1 (Part-B), Section 4.2, Section 4.3.8, Section 6.1 Table-3, Section 6.1.3, Section 6.1.4, Section 6.1.5.3, Section 6.1.5.4, Section 6.5.1, Section 6.5.2, Section 6.6, Section 7.7 and Table 4 Appendix K (Appendix K)	Additional time points for ADAs sample collection against BIVV009 have been added. New exploratory objective of immunogenicity has also been added.	
Synopsis, Section 4.2, Section 4.3.6, Section 4.3.7, Section 6.1 Table -3, section 6.1.5.3, Section 6.1.5.4, Section 6.5.1, Section 6.5.2 and Table 4 Appendix K (Appendix K).	Time points when PK and PD samples are to be collected during Part B has been clarified.	
Section 6.1 Table-3, Section 6.1.5.3, and Table 4 Appendix K (Appendix K).	New time points for SLE panel in Part B have been added	
Section 4.2, Section 4.3.8	Mentioned that ADA against BIVV009 may be assessed using available predose PD back-up samples for patients having consented to the use of their blood samples for future research.	
Appendix A	“Iron” was deleted from the clinical chemistry panel list.	

Three new appendix sections have also been created to add “Home Infusion Patient Satisfaction Questionnaire” (Appendix L), to display “Protocol Amendments History” (Appendix J), and to add “Country Specific Requirements- home infusions with BIVV009” (Appendix K).

Protocol Version 5:

Protocol Version	Description of Change	Brief Rationale
Protocol Version 5 (from Version 3)	Synopsis, Section 2.2.2, Section 4, Section 5.1, Section 6, Table -3 and appendices have been updated.	The primary reason for this amendment to Protocol BIVV009-04 was to incorporate the following changes:<br>- The study number was revised.<br>- The PGIS was added per FDA request.<br>- "Documented" was added to clarify that previous vaccinations must be confirmed with vaccination records rather than just patient report. "Where available" was added because vaccination against serogroup B meningococcus isn't available in all of the participating countries.<br>- The start date for the collection of PK & PD samples during Part B was corrected.<br>- BIVV009-01 study information was revised for clarity and updated to include Part E.<br>- To reduce the number of patients in the Cardinal study who are minimally transfused.<br>- The vaccination schedule from the country-specific protocol for Japan (Version 3.1) was added to the vaccination schedule for the other participation countries in this version of the protocol for completeness.<br>- Windows were added for the post-dose vital signs, ECGs, and PK/PD sampling time points for completeness.<br>- BIVV009 to be supplied in larger volume and concentration.
Protocol Version 4 (not implemented)	Synopsis, Section 2.2.2, Section 4, Section 5.1, Section 6, Table -3, appendices have been updated.	The primary reason for this amendment to Protocol BIVV009-04 was to incorporate the following changes:<br>- The study number was revised.<br>- The PGIS was added per FDA request.<br>- The start date for the collection of PK & PD samples during Part B was corrected.

Protocol Version	Description of Change	Brief Rationale
		- BIVV009-01 study information was revised for clarity and updated to include Part E.<br>- To reduce the number of patients in the Cardinal study who are minimally transfused.<br>- The vaccination schedule from the country-specific protocol for Japan (Version 3.1) was added to the vaccination schedule for the other participation countries in this version of the protocol for completeness.<br>- Windows were added for the post-dose vital signs, ECGs, and PK/PD sampling time points for completeness.<br>- BIVV009 to be supplied in larger volume and concentration.<br>- The frequency of PGIC assessment time points was updated to every 3 months<br>- Footnote "r" was added to show the order the assessments are to be conducted<br>- The PGIS and PGIC assessments were added at the same time points as the SF-12
Protocol Version 3.1 (from Version 3)	Synopsis, Section 5.1, Section 6.1.1.1, Section 6.1.1.2, and Table-3 were updated and Appendix-H was added.	The primary reason for this amendment to Protocol BIVV009-04 was to incorporate the following changes:<br>- The inclusion criterion on vaccinations was changed.<br>- Instructions on vaccination were updated.
Protocol Version 3 (from Version 1.1)	Synopsis, Section 2.2, Section 2.2.2, Section 2.2.3, Section 2.2.4, Section 4.1, Section 4.3.3, Section 4.3.4, Section 4.4, Section 4.5, Section 5.1, Section 5.2, Section 5.3, Table -3, Section 6.1.1.1, Section 6.1.2, Section 6.1.3, Section 6.1.4, and Section 6.1.5, Section 6.1.6, Section 6.2.4, Section 6.2.5, and Section 6.2.7, Section 6.3.6, Section 6.3.8, Section 6.7, Section 6.7.1.1, Section 6.7.1.2, Section 6.7.1.3, Section 6.7.2, Section 6.7.4, Section 6.7.5, Section 6.7.7, and Section 9 have been updated.	The primary reason for this amendment to Protocol BIVV009-04 was to incorporate the following changes:<br>- The original Appendix B (Vaccination Schedule – Encapsulated Bacterial Pathogens) was removed and additional instructions were added to Section 6.1.1.1 (reference to the vaccination schedule was changed from Appendix B to Section 6.1.1.1).

Protocol Version	Description of Change	Brief Rationale
		- The original Appendix C (Guidelines for the Diagnosis and Treatment of Hypersensitivity Reactions and Anaphylaxis) also was removed.<br>- A healthcare resource utilization appendix was added.<br>- The current appendices are labeled per below:<br>Appendix A: Clinical Laboratory Evaluations<br>Appendix B: NCI-CTC Version 4.03 (CTCAE V4.03)<br>Appendix C: Functional Assessment of Chronic Illness Therapy (FACIT) – Fatigue Scale<br>Appendix D: EQ-5D-5L<br>Appendix E: 12-Item Short Form Survey (SF-12)<br>Appendix F: Patient's Global Impression of Change (PGIC)<br>Appendix G: Healthcare Resource Utilization<br>- The number of patients was changed from 40 to approximately 40 patients.<br>- Hemolytic "flare" was changed to hemolytic breakthrough.<br>- Beginning on Day 0, urine pregnancy testing was changed to serum or urine pregnancy testing.<br>- Phase 3 was added to the study title.
Protocol Version 1.6 (from Version 1.1)	Section 6.2.5 has been updated.	The primary reason for this amendment to Protocol BIVV009-04 was to incorporate the following changes:<br>- New instructions on blinding/unblinding procedure in Section 6.2.5 have been added.
Protocol Version 1.5 (from Version 1.1)	Table 3 (Study schedule of events), Section 6.1.3, and Section 6.1.5 have been updated.	The primary reason for this amendment to Protocol BIVV009-04 was to incorporate the following changes:<br>- Pregnancy test instructions were modified.

Protocol Version	Description of Change	Brief Rationale
Protocol Version 1.4 (from Version 1.1)	Synopsis, Section 4.1, Section 4.4, Section 4.5, Section 5.1, Section 5.2, Section 5.3, Table-3, Section 6.1.5, Section 6.1.6, Section 6.2.5, Section 6.2.7, Section 6.7, Section 6.7.4, and Section 6.7.7 have been updated.	The primary reason for this amendment to Protocol BIVV009-04 was to incorporate the following changes:<br>- Global: For clarity, hemolytic “flare” was changed to hemolytic breakthrough.<br>- Blinding/unblinding instructions were added<br>- Inclusion criteria (bilirubin level, use of contraception) and one exclusion criterion (other autoimmune disorders) were modified.<br>- Part A/B duration of treatment was changed from every 6 weeks to 9 weeks.
Protocol Version 1.3 (from Version 1.1)	Synopsis, Section 4.1, Section 4.5, Section 5.1, Section 5.2, Table-3, Section 6.1.3, Section 6.1.5, and Section 6.1.6, Section 6.2.5, and Section 6.7.1.3 have been updated.	The primary reason for this amendment to Protocol BIVV009-04 was to incorporate the following changes:<br>- The Part B study duration was changed from approximately 1 year to 1 year.<br>- Ferritin level at baseline was adjusted in inclusion criteria and exclusion criteria.<br>- Urine pregnancy testing frequency was changed from every 3 months to every 4 weeks.<br>- Blinding/unblinding instructions were added.<br>- Unexpected adverse event/SUSAR data collection instruction was modified.
Protocol Version 1.2 (from Version 1.1)	Synopsis, Section 2.2.2, Section 4.1, Section 4.5, Section 5.1, Table -3, Section 6.1.1.1, Section 6.1.6, Section 6.2.5, and Section 6.3.6 have been updated.	The primary reason for this amendment to Protocol BIVV009-04 was to incorporate the following changes:<br>- The original Appendix B (Vaccination Schedule – Encapsulated Bacterial Pathogens) was removed and additional instructions were added to Section 6.1.1.1 (reference to the vaccination schedule was changed from Appendix B to Section 6.1.1.1).<br>- The appendices were relabeled as shown below.

Protocol Version	Description of Change	Brief Rationale
		Appendix A: Clinical Laboratory Evaluations<br>Appendix B: Guidelines for the Diagnosis and Treatment of Hypersensitivity Reactions and Anaphylaxis<br>Appendix C: NCI-CTC Version 4.03 (CTCAE V4.03)<br>Appendix D: Functional Assessment of Chronic Illness Therapy (FACIT) – Fatigue Scale<br>Appendix E: EQ-5D-5L<br>Appendix F: 12-Item Short Form Survey (SF-12)<br>Appendix G: Patient's Global Impression of Change (PGIC)
Protocol Version 1.1	The company name, True North Therapeutics, Inc. was replaced by Bioverativ USA throughout the document.<br>Exploratory efficacy end-point, "Patients Global Impression of Severity (PGIS)" was deleted throughout the document.	The primary reason for this amendment to Protocol BIVV009-04 was to incorporate a change in responsibility and obligations of the study sponsor and to delete Exploratory efficacy end-point, PGIS and changes in time point of 12 lead ECG.

Appendix K COUNTRY SPECIFIC REQUIREMENT – HOME INFUSIONS WITH BIVV009

Compared to the global protocol, the following country specific changes are applicable to the US, the Netherlands, Norway, France, Italy, Austria, Germany, and Spain:

Synopsis (Objectives)

Part B

Exploratory objective:

- To describe the safety and patient satisfaction with the convenience of home infusions with BIVV009 in a subset of patients.

Synopsis (Test Product(s), Dose, and Mode of Administration)

Home infusion will be performed at pre-selected countries/sites by a healthcare professional caregiver contingent upon completion of training delivered by the investigator or delegated site staff member only in patients willing to be administered study drug in home, who have been treated for at least 3 months (completed visit Day 273) with the study drug BIVV009 in Part B, in whom previous on-site infusions were uncomplicated, and who satisfy the following criteria:

1. Able to comprehend and give informed consent for participation in the alternate home infusion scheme and willing to be infused at home for at least 4 doses, alternating with regular office visits.
2. Treated for at least 3 months in Part B (completed visit Day 273)
3. No history of hypersensitivity reaction to BIVV009
4. Considered by the investigator to be a good candidate for home infusions
5. Not participating in, or have never participated in undiluted infusion administration

Qualifying patients who consent for home infusions will have study drug administered in home every other bi-weekly visit alternating with regular office visit with drug infusion. Qualification for home infusions and informed consent will be collected no later than at office visit two weeks before first planned home infusion.

For subjects receiving drug injections in home as administered by a healthcare professional during the Part B open-label phase, the healthcare professional will collect during home visits specific information (AEs, concomitant medications, solicited anemia assessment, as well as, after the 1st and 4th home infusions, the patient will answer (by filling up the form) the Home Infusion Patient Satisfaction Survey) (Appendix L). The data collected on dedicated paper forms will be provided to the Investigator within 24 hours for entry in EDC. The healthcare professional will monitor the subject during infusion lasting 1-hour or, in certain patients 2-hour (eg, patients with underlying cardiopulmonary disease, with sponsor's approval) and for 2 hours after the first home infusion dose and 1 hour after the completion of each subsequent home infusion. Any adverse events occurring during home visits will be immediately reported by the healthcare professional caregiver to the Investigator.

First office visit 2 weeks after first home infusion will include assessments normally planned to be performed every 3 months. Neither blood samples nor urine or serum pregnancy tests will be performed during home infusion visits; blood tests scheduled to be performed at 2, or 4 weeks intervals as well urine or serum pregnancy tests in WOCBP (ie, women of child bearing potential) will be performed during office visits every four weeks while the patient is within the home infusion scheme.

In case a scheduled home infusion visit happens to coincide with an every 3 month office visit, rather than this visit occurring at home, the patient should attend the office visit with study drug infused at the office site. The next home infusion visit will then occur 2 weeks later. Subsequently, the bi-weekly alternating office visit and home visits with drug infusions will then resume as previously planned.

Patients who miss a dose (ie, outside the dosing window or >17 days since last dose, regardless if last dose was given at home or at the site) should return to the site for an unscheduled visit to receive another loading dose prior to their next scheduled visit which will also take place at the office. Such patient may return to the schedule of home visits alternating with regular office visits if eligibility criteria for home infusions are still met.

Patients undergoing home infusions with BIVV009 will return to bi-weekly dosing at the study site if they experience adverse event that in the opinion of the Investigator, may jeopardize patient safety if home infusions are continued. Patients may return to home infusions if this AE is considered by the Investigator to be unrelated to BIVV0009 and once resolved or stabilized. Hypersensitivity or allergic reactions to study drug will always result in study drug discontinuation and withdrawal from the study.

Infusions given in the home setting versus at the investigational site will be captured through the case report form (CRF) for drug administration.

Table 4 - Study schedule of events (Part B) for patients with home infusions after Day 273

Study Visit (Week/Day)	Part B post Day 273 Open-Label Extension Phase	Part B post Day 273 Open-Label Extension Phase [m]	ET/EOS/Safety Follow-up [a]
	On site visit	Home visit	9 Weeks after Last Dose On site visit
**Visit Windows**	**± 2 days**	**± 2 days**	**± 2 days**
Pregnancy test (if applicable) [b]	X		X
Body weight	X[h]		X
Physical examination, full			X
Physical examination, brief	X[h]		
Vital signs (BP, PR, RR, body temperature) [c]	X[h]		X
SLE panel [d]	X[d]		X

Study Visit (Week/Day)	Part B post Day 273 Open-Label Extension Phase	Part B post Day 273 Open-Label Extension Phase [m]	ET/EOS/Safety Follow-up [a]
	On site visit	Home visit	9 Weeks after Last Dose On site visit
**Visit Windows**	**± 2 days**	**± 2 days**	**± 2 days**
Hematology panel [d]	X		X
Coagulation panel [d]			X
Clinical chemistry panel [d]	X		X
Urinalysis [d]	X[h]		X
FACIT-Fatigue [j]	X[h]		X
PGIS [j]	X[h]		X
PGIC [j]	X[h]		X
SF-12 [j]	X[h]		X
EQ-5D-5L [j]	X[h]		X
Solicited symptomatic anemia	X	X	X
Study drug administration [e]	X	X	
ADAs against BIVV009 and neutralizing anti bodies	X[k]		X
PK Samples [f]	X[h]		X
PD Samples [d, f]	X[h]		X
Concomitant medications including transfusions	X	X	X
Healthcare resource utilization	X[i]		X
Adverse events [g]	X	X[n]	X
Home infusion satisfaction survey		X[l]	

ADA = anti-drug antibodies; BP = blood pressure; EOS = End of Study; EQ-5D-5L = five level EuroQol five dimensions questionnaire; ET = Early Termination Visit; FACIT-Fatigue = functional assessment of chronic illness therapy - fatigue; N/A = not applicable; PD = pharmacodynamic; PGIC = Patient's Global Impression of Change; PGIS = Patient's Global Impression of [Fatigue] Severity; PK = pharmacokinetic; PR = pulse rate; QOL = quality of life; RR = respiratory rate; SF-12 = 12-Item Short Form Survey;

[a] Patients should return to site 9 weeks after last dose for ET procedures, EOS assessment, or Safety Follow-up procedures upon completion of dosing in the study. If patient experiences a hematological breakthrough event, a PK, PD, and ADA sample should be collected at the time of the event.
[b] Females of child-bearing potential only. Serum or urine pregnancy test every 4 weeks (+/- 2 days) during Part B at on-site visit and at ET/EOS safety follow-up visit
[c] Vital signs measurements (supine BP, PR, RR, and oral temperature) are to be obtained with measurements performed predose and 1 hour (±5 minutes) after completion of administration of each dose of study drug (on site visits only).
[d] For a complete list of analytes, see protocol Appendix A. SLE panel testing should occur every 6 months at on site visits in Part B.
[e] Study drug doses of 6.5 grams (if <75 kg) or 7.5 grams (if ≥75 kg) based on patient's baseline body weight will be administered via IV infusion over ~ 60±5 minutes, every 2 weeks during Part B. Patients with underlying cardiopulmonary disease may receive a 2-hour infusion with Sponsor approval. If a patient misses a scheduled dose (outside of the 2-day window or >17 days since last dose), they must return to site (unscheduled visit) to receive another loading dose 1 week prior to the next scheduled dose.

[f] During Part B, PK and PD samples will continue to be collected routinely at predose and 1 hour (±15 minutes) postdose at 3-month intervals starting at Day 273 through the remainder of the study. PK and PD samples will also be collected if a patient experiences a hematologic breakthrough event at any point during the study.
[g] AEs will be recorded from the time the patient signs the informed consent form until 9 weeks after administration of the last dose of study drug. For patients with home infusions, safety assessments will additionally include AEs with onset within 24 hours of the of infusion.
[h] To be performed every 3 months during the on-site visits.
[i] During Part B, the healthcare resource utilization data will be recorded every 4 weeks, at on site visit;
[j] To be performed in the following order: FACIT-Fatigue first, PGIS second, PGIC third, SF-12 fourth, and EQ-5D-5L fifth.
[k] During Part B post Day 273, ADA samples will be collected every 3 months predose, and at Safety Follow up visit 9 weeks after last dose. Samples will also be collected if a patient experiences a hematologic breakthrough event or withdraws from the study early.
[l] After the 1st and 4th home infusions
[m] Post-Day 273 extension phase home visit is alternating with every 4 weeks with site visit and if needed, a loading dose can be infused at the site visit.
[n] HCP records and AEs will be collected on paper forms and will be forwarded to PI within 24h; site will be informed of any AEs immediately; All SAE reporting timelines still apply (ie need to be reported to sponsor safety database within 24 hours of awareness by the home infusion healthcare professional).

Section 2.2 (Background and Study Rationale)

Patients with CAgD are often elderly and/or have numerous co-morbidities affecting their mobility. Moreover, clinical centers specialized in the management of CAgD are infrequent and may be located far from patients' home. Consequently, some patients may find the option of home infusions with drug against CAgD, beneficial. To this end a group of patients at preselected sites/countries will be offered the possibility of home infusions during Part B, assisted by trained health care professional.

Section 2.2.4 (Potential Risks and Benefits)

Home infusions with the study drug will be proposed to a number of patients in countries pre-selected to participate in home infusion. Home infusions will be assisted by a trained healthcare professional, and will concern patients who express such wish, after having been qualified by the Investigator and no sooner than after Week 39 (Day 273) and without evidence of hypersensitivity to study drug. Only patients treated with BIVV009 for the minimum of 3 months and in whom previous infusions with BIVV009 were not associated with a hypersensitivity reaction are eligible for the home infusion. Additionally, patients who are participating in or have ever participated in undiluted infusions are not eligible for home infusions. Rescue medications to treat hypersensitivity/allergic reactions as per the PI's guidance will be immediately available during home infusion visits. The home infusion personnel will be trained in basic life support (cardiopulmonary resuscitation [CPR]) but will not be required to have equipment to perform advanced life support (eg, defibrillators).

Section 3.5 (Exploratory objective [Part B])

- To describe the safety and patient satisfaction with the convenience of home infusions with BIVV009 in a subset of patients in the US, the Netherlands, Norway, France, Italy, Austria, Germany, and Spain.

Section 4.1 (Study Design: Part-B)

A subset of patients from selected sites/countries, who have been treated for a minimum 3 months (completed visit Day 273) in Part B, and who were determined to have tolerated BIVV009 well, will be invited to have infusions with BIVV009 performed at their homes, after

having been qualified by the Investigator. Home infusions will be carried out by a trained nurse. Patients will follow the alternate home infusion scheme, ie, home infusion at the patient's home will be alternating with office visits, so that patients will attend office visit every 4 weeks alternating with home infusions every 4 weeks (+/- 2 days).

Section 6.1, Table -3 (Footnote "i")

Study drug doses of 6.5 grams (if <75 kg) or 7.5 grams (if ≥75 kg) based on patient's baseline body weight will be administered via IV infusion over ~ 60±5 minutes on Days 0, 7, and every 14 days thereafter during Part A and every 2 weeks starting at Week 27 during Part B. Patients with underlying cardiopulmonary disease may receive a 2-hour infusion with Sponsor approval. If a patient misses a scheduled dose (outside of the 2-day window or >17 days since last dose), they must return to site (unscheduled visit) to receive another loading dose 1 week prior to the next scheduled dose. Qualifying patients at participating sites may have study drug dosed at home, alternating with study drug dosed during office visits in Part B.

Section 6.1.5.5 (Infusion of the study drug at patient's home)

Home infusions will be performed at pre-selected countries/sites by a healthcare professional caregiver contingent upon completion of training delivered by the Investigator or delegated site staff member only in patients willing to be administered study drug at home, who have been treated for at least 3 months (completed visit Day 273) with the study drug BIVV009 in Part B, and in whom previous on-site infusions were uncomplicated, who satisfy the following criteria:

1. Able to comprehend and give informed consent for participation in the alternate home infusion scheme and willing to be infused at home for at least 4 doses, alternating with regular office visits.
2. Treated for at least 3 months in Part B (completed visit Day 273)
3. No history of hypersensitivity reaction to BIVV009
4. Considered by the investigator to be a good candidate for home infusions
5. Not participating in, or have never participated in undiluted infusion administration

Qualifying patients who consent for home infusions will have study drug administered in home every other bi-weekly visit alternating with regular office visit with drug infusion. Qualification for home infusions and informed consent will be collected no later than at office visit two weeks before first planned home infusion.

For subjects receiving drug injections in home as administered by a healthcare professional during the Part B open-label phase, the healthcare professional will collect specific information (AEs, concomitant medications, solicited anemia assessment as well as, after the 1st and 4th home infusions, the patient will answer the Home Infusion Patient Satisfaction Survey). The data collected on dedicated paper forms will be provided to the Investigator within 24 hours for entry in EDC. The healthcare professional will monitor the subject during 1-hour or, in certain patients 2-hour infusion (eg, patients with underlying cardiopulmonary disease, with sponsor's approval) and for at least 1 hour after the completion of the first home infusion or 2 hours after the

completion of each subsequent home infusion. Any adverse events occurring during home visits will be immediately reported by the healthcare professional caregiver to the Investigator.

First office visit 2 weeks after first home infusion will include assessments normally planned to be performed every 3 months. Neither blood samples nor urine pregnancy tests will be collected/performed during home infusion visits; blood tests scheduled to be performed at 2, or 4 weeks intervals as well urine pregnancy tests in WOCBP (ie, women of child bearing potential) will be performed during office visits every four weeks while the patient is within the home infusion scheme.

In case a scheduled home infusion visit happens to coincide with an every 3 month office visit, rather than this visit occurring at home, the patient should attend the office visit with study drug infused at the office site. The next home infusion visit will then occur 2 weeks later. Subsequently, the bi-weekly alternating office and home visits with drug infusions will then resume as previously planned.

Patients who miss a dose (ie, outside the dosing window or >17 days since last dose, regardless if last dose was given at home or at the site) should return to the site for an unscheduled visit to receive another loading dose prior to their next scheduled visit which will also take place at the office. Such patient may return to the schedule of home visits alternating with regular office visits if eligibility criteria for home infusions are still met.

Patients undergoing home infusions with BIVV009 will return to bi-weekly dosing at the study site if they experience adverse event that in the opinion of the Investigator, may jeopardize patient safety if home infusions are continued. Patients may return to home infusions if this AE is considered by the Investigator to be unrelated to BIVV0009 and once resolved or stabilized. Hypersensitivity or allergic reactions to study drug will always result in study drug discontinuation and withdrawal from the study.

Infusions given in the home setting versus at the investigational site will be captured through the case report form (CRF) for drug administration.

Section 6.2.1 (Drug Supplies and Accountability)

Preparation and accountability procedures for the investigational product, including those associated with home infusions whenever applicable, are described in further detail in the Pharmacy Manual.

Section 6.2.4 (Dose Preparation and Administration)

Patients must be monitored for acute allergic reactions during infusion and for at least 2 hours after the completion of the first administration of study drug at a home infusion visit or 1 hour after the completion of each administration of study drug thereafter at subsequent home infusion visits.

Epinephrine, parenteral diphenhydramine and, for infusions carried at the study site, cardiac resuscitation equipment, must be immediately available in case an allergic reaction or anaphylaxis occurs. Site personnel or professional healthcare caregiver in case of home infusions

(Appendix K), must be qualified to detect allergic reactions and anaphylaxis and treat those reactions under the guidance of the PI. The home infusion personnel will be trained in basic life support (cardiopulmonary resuscitation [CPR]) but will not be required to have equipment to pTerform advanced life support (eg, defibrillators). See Section 6.3.8 for further details regarding additional testing.

Patients should be instructed to report the development of rash, hives, pruritus, flushing, urticaria, etc, that may represent an allergic or hypersensitivity reaction to study drug. If any signs or symptoms of an allergic or anaphylactic reaction are observed during the infusion, administration of study drug must be immediately discontinued and the patient treated as appropriate. In case of home infusion, the investigator has to be notified immediately to guide treatment.

Appendix L HOME INFUSION PATIENT SATISFACTION SURVEY

How satisfied or dissatisfied are you with the overall convenience of being infused at home?

Very satisfied	Satisfied	Dissatisfied	Very dissatisfied
☐ 1	☐ 2	☐ 3	☐ 4

Signature Page for VV-CLIN-0550477 v3.0
bivv009-04-16-1-1-cadenza-protocol-v8

Approve & eSign	Karin Knobe<br>Clinical<br>06-Nov-2020 08:44:14 GMT+0000

Approve & eSign	Corinne Hanotin<br>Clinical<br>07-Nov-2020 11:42:46 GMT+0000
